# CMS pythia  8 colour reconnection tunes based on underlying-event data

**DOI:** 10.1140/epjc/s10052-023-11630-8

**Published:** 2023-07-10

**Authors:** A. Tumasyan, W. Adam, J. W. Andrejkovic, T. Bergauer, S. Chatterjee, K. Damanakis, M. Dragicevic, A. Escalante Del Valle, R. Frühwirth, M. Jeitler, N. Krammer, L. Lechner, D. Liko, I. Mikulec, P. Paulitsch, F. M. Pitters, J. Schieck, R. Schöfbeck, D. Schwarz, S. Templ, W. Waltenberger, C.-E. Wulz, M. R. Darwish, E. A. De Wolf, T. Janssen, T. Kello, A. Lelek, H. Rejeb Sfar, P. Van Mechelen, S. Van Putte, N. Van Remortel, E. S. Bols, J. D’Hondt, M. Delcourt, H. El Faham, S. Lowette, S. Moortgat, A. Morton, D. Müller, A. R. Sahasransu, S. Tavernier, W. Van Doninck, D. Vannerom, D. Beghin, B. Bilin, B. Clerbaux, G. De Lentdecker, L. Favart, A. K. Kalsi, K. Lee, M. Mahdavikhorrami, I. Makarenko, L. Moureaux, S. Paredes, L. Pétré, A. Popov, N. Postiau, E. Starling, L. Thomas, M. Vanden Bemden, C. Vander Velde, P. Vanlaer, T. Cornelis, D. Dobur, J. Knolle, L. Lambrecht, G. Mestdach, M. Niedziela, C. Rendón, C. Roskas, A. Samalan, K. Skovpen, M. Tytgat, B. Vermassen, L. Wezenbeek, A. Benecke, A. Bethani, G. Bruno, F. Bury, C. Caputo, P. David, C. Delaere, I. S. Donertas, A. Giammanco, K. Jaffel, Sa. Jain, V. Lemaitre, K. Mondal, J. Prisciandaro, A. Taliercio, M. Teklishyn, T. T. Tran, P. Vischia, S. Wertz, G. A. Alves, C. Hensel, A. Moraes, P. Rebello Teles, W. L. Aldá Júnior, M. Alves Gallo Pereira, M. Barroso Ferreira Filho, H. Brandao Malbouisson, W. Carvalho, J. Chinellato, E. M. Da Costa, G. G. Da Silveira, D. De Jesus Damiao, V. Dos Santos Sousa, S. Fonseca De Souza, C. Mora Herrera, K. Mota Amarilo, L. Mundim, H. Nogima, A. Santoro, S. M. Silva Do Amaral, A. Sznajder, M. Thiel, F. Torres Da Silva De Araujo, A. Vilela Pereira, C. A. Bernardes, L. Calligaris, T. R. Fernandez Perez Tomei, E. M. Gregores, D. S. Lemos, P. G. Mercadante, S. F. Novaes, Sandra S. Padula, A. Aleksandrov, G. Antchev, R. Hadjiiska, P. Iaydjiev, M. Misheva, M. Rodozov, M. Shopova, G. Sultanov, A. Dimitrov, T. Ivanov, L. Litov, B. Pavlov, P. Petkov, A. Petrov, T. Cheng, T. Javaid, M. Mittal, L. Yuan, M. Ahmad, G. Bauer, C. Dozen, Z. Hu, J. Martins, Y. Wang, K. Yi, E. Chapon, G. M. Chen, H. S. Chen, M. Chen, F. Iemmi, A. Kapoor, D. Leggat, H. Liao, Z.-A. Liu, V. Milosevic, F. Monti, R. Sharma, J. Tao, J. Thomas-Wilsker, J. Wang, H. Zhang, J. Zhao, A. Agapitos, Y. An, Y. Ban, C. Chen, A. Levin, Q. Li, X. Lyu, Y. Mao, S. J. Qian, D. Wang, J. Xiao, H. Yang, M. Lu, Z. You, X. Gao, H. Okawa, Y. Zhang, Z. Lin, M. Xiao, C. Avila, A. Cabrera, C. Florez, J. Fraga, J. Mejia Guisao, F. Ramirez, J. D. Ruiz Alvarez, D. Giljanovic, N. Godinovic, D. Lelas, I. Puljak, Z. Antunovic, M. Kovac, T. Sculac, V. Brigljevic, D. Ferencek, D. Majumder, M. Roguljic, A. Starodumov, T. Susa, A. Attikis, K. Christoforou, A. Ioannou, G. Kole, M. Kolosova, S. Konstantinou, J. Mousa, C. Nicolaou, F. Ptochos, P. A. Razis, H. Rykaczewski, H. Saka, M. Finger, M. Finger, A. Kveton, E. Ayala, E. Carrera Jarrin, A. A. Abdelalim, S. Khalil, M. A. Mahmoud, Y. Mohammed, S. Bhowmik, R. K. Dewanjee, K. Ehataht, M. Kadastik, S. Nandan, C. Nielsen, J. Pata, M. Raidal, L. Tani, C. Veelken, P. Eerola, H. Kirschenmann, K. Osterberg, M. Voutilainen, S. Bharthuar, E. Brücken, F. Garcia, J. Havukainen, M. S. Kim, R. Kinnunen, T. Lampén, K. Lassila-Perini, S. Lehti, T. Lindén, M. Lotti, L. Martikainen, M. Myllymäki, J. Ott, H. Siikonen, E. Tuominen, J. Tuominiemi, P. Luukka, H. Petrow, T. Tuuva, C. Amendola, M. Besancon, F. Couderc, M. Dejardin, D. Denegri, J. L. Faure, F. Ferri, S. Ganjour, P. Gras, G. Hamel de Monchenault, P. Jarry, B. Lenzi, E. Locci, J. Malcles, J. Rander, A. Rosowsky, M.Ö. Sahin, A. Savoy-Navarro, M. Titov, G. B. Yu, S. Ahuja, F. Beaudette, M. Bonanomi, A. Buchot Perraguin, P. Busson, A. Cappati, C. Charlot, O. Davignon, B. Diab, G. Falmagne, S. Ghosh, R. Granier de Cassagnac, A. Hakimi, I. Kucher, J. Motta, M. Nguyen, C. Ochando, P. Paganini, J. Rembser, R. Salerno, U. Sarkar, J. B. Sauvan, Y. Sirois, A. Tarabini, A. Zabi, A. Zghiche, J.-L. Agram, J. Andrea, D. Apparu, D. Bloch, G. Bourgatte, J.-M. Brom, E. C. Chabert, C. Collard, D. Darej, J.-C. Fontaine, U. Goerlach, C. Grimault, A.-C. Le Bihan, E. Nibigira, P. Van Hove, E. Asilar, S. Beauceron, C. Bernet, G. Boudoul, C. Camen, A. Carle, N. Chanon, D. Contardo, P. Depasse, H. El Mamouni, J. Fay, S. Gascon, M. Gouzevitch, B. Ille, I. B. Laktineh, H. Lattaud, A. Lesauvage, M. Lethuillier, L. Mirabito, S. Perries, K. Shchablo, V. Sordini, L. Torterotot, G. Touquet, M. Vander Donckt, S. Viret, I. Lomidze, T. Toriashvili, Z. Tsamalaidze, V. Botta, L. Feld, K. Klein, M. Lipinski, D. Meuser, A. Pauls, N. Röwert, J. Schulz, M. Teroerde, A. Dodonova, D. Eliseev, M. Erdmann, P. Fackeldey, B. Fischer, T. Hebbeker, K. Hoepfner, F. Ivone, L. Mastrolorenzo, M. Merschmeyer, A. Meyer, G. Mocellin, S. Mondal, S. Mukherjee, D. Noll, A. Novak, A. Pozdnyakov, Y. Rath, H. Reithler, A. Schmidt, S. C. Schuler, A. Sharma, L. Vigilante, S. Wiedenbeck, S. Zaleski, C. Dziwok, G. Flügge, W. Haj Ahmad, O. Hlushchenko, T. Kress, A. Nowack, O. Pooth, D. Roy, A. Stahl, T. Ziemons, A. Zotz, H. Aarup Petersen, M. Aldaya Martin, P. Asmuss, S. Baxter, M. Bayatmakou, O. Behnke, A. Bermúdez Martínez, S. Bhattacharya, A. A. Bin Anuar, F. Blekman, K. Borras, D. Brunner, A. Campbell, A. Cardini, C. Cheng, F. Colombina, S. Consuegra Rodríguez, G. Correia Silva, V. Danilov, M. De Silva, L. Didukh, G. Eckerlin, D. Eckstein, L. I. Estevez Banos, O. Filatov, E. Gallo, A. Geiser, A. Giraldi, A. Grohsjean, M. Guthoff, A. Jafari, N. Z. Jomhari, A. Kasem, M. Kasemann, H. Kaveh, C. Kleinwort, R. Kogler, D. Krücker, W. Lange, K. Lipka, W. Lohmann, R. Mankel, I.-A. Melzer-Pellmann, M. Mendizabal Morentin, J. Metwally, A. B. Meyer, M. Meyer, J. Mnich, A. Mussgiller, A. Nürnberg, Y. Otarid, D. Pérez Adán, D. Pitzl, A. Raspereza, B. Ribeiro Lopes, J. Rübenach, A. Saggio, A. Saibel, M. Savitskyi, M. Scham, V. Scheurer, S. Schnake, P. Schütze, C. Schwanenberger, M. Shchedrolosiev, R. E. Sosa Ricardo, D. Stafford, N. Tonon, M. Van De Klundert, F. Vazzoler, R. Walsh, D. Walter, Q. Wang, Y. Wen, K. Wichmann, L. Wiens, C. Wissing, S. Wuchterl, R. Aggleton, S. Albrecht, S. Bein, L. Benato, P. Connor, K. De Leo, M. Eich, F. Feindt, A. Fröhlich, C. Garbers, E. Garutti, P. Gunnellini, M. Hajheidari, J. Haller, A. Hinzmann, G. Kasieczka, R. Klanner, T. Kramer, V. Kutzner, J. Lange, T. Lange, A. Lobanov, A. Malara, A. Mehta, A. Nigamova, K. J. Pena Rodriguez, M. Rieger, O. Rieger, P. Schleper, M. Schröder, J. Schwandt, J. Sonneveld, H. Stadie, G. Steinbrück, A. Tews, I. Zoi, J. Bechtel, S. Brommer, M. Burkart, E. Butz, R. Caspart, T. Chwalek, W. De Boer, A. Dierlamm, A. Droll, K. El Morabit, N. Faltermann, M. Giffels, J. O. Gosewisch, A. Gottmann, F. Hartmann, C. Heidecker, U. Husemann, P. Keicher, R. Koppenhöfer, S. Maier, M. Metzler, S. Mitra, Th. Müller, M. Neukum, G. Quast, K. Rabbertz, J. Rauser, D. Savoiu, M. Schnepf, D. Seith, I. Shvetsov, H. J. Simonis, R. Ulrich, J. Van Der Linden, R. F. Von Cube, M. Wassmer, M. Weber, S. Wieland, R. Wolf, S. Wozniewski, S. Wunsch, G. Anagnostou, G. Daskalakis, A. Kyriakis, A. Stakia, M. Diamantopoulou, D. Karasavvas, P. Kontaxakis, C. K. Koraka, A. Manousakis-Katsikakis, A. Panagiotou, I. Papavergou, N. Saoulidou, K. Theofilatos, E. Tziaferi, K. Vellidis, E. Vourliotis, G. Bakas, K. Kousouris, I. Papakrivopoulos, G. Tsipolitis, A. Zacharopoulou, K. Adamidis, I. Bestintzanos, I. Evangelou, C. Foudas, P. Gianneios, P. Katsoulis, P. Kokkas, N. Manthos, I. Papadopoulos, J. Strologas, M. Csanád, K. Farkas, M. M. A. Gadallah, S. Lökös, P. Major, K. Mandal, G. Pásztor, A. J. Rádl, O. Surányi, G. I. Veres, M. Bartók, G. Bencze, C. Hajdu, D. Horvath, F. Sikler, V. Veszpremi, S. Czellar, D. Fasanella, F. Fienga, J. Karancsi, J. Molnar, Z. Szillasi, D. Teyssier, P. Raics, Z. L. Trocsanyi, B. Ujvari, T. Csorgo, F. Nemes, T. Novak, S. Bansal, S. B. Beri, V. Bhatnagar, G. Chaudhary, S. Chauhan, N. Dhingra, R. Gupta, A. Kaur, H. Kaur, M. Kaur, P. Kumari, M. Meena, K. Sandeep, J. B. Singh, A. K. Virdi, A. Ahmed, A. Bhardwaj, B. C. Choudhary, M. Gola, S. Keshri, A. Kumar, M. Naimuddin, P. Priyanka, K. Ranjan, A. Shah, M. Bharti, R. Bhattacharya, S. Bhattacharya, D. Bhowmik, S. Dutta, S. Dutta, B. Gomber, M. Maity, P. Palit, P. K. Rout, G. Saha, B. Sahu, S. Sarkar, M. Sharan, P. K. Behera, S. C. Behera, P. Kalbhor, J. R. Komaragiri, D. Kumar, A. Muhammad, L. Panwar, R. Pradhan, P. R. Pujahari, A. Sharma, A. K. Sikdar, P. C. Tiwari, K. Naskar, T. Aziz, S. Dugad, M. Kumar, G. B. Mohanty, S. Banerjee, R. Chudasama, M. Guchait, S. Karmakar, S. Kumar, G. Majumder, K. Mazumdar, S. Mukherjee, S. Bahinipati, C. Kar, P. Mal, T. Mishra, V. K. Muraleedharan Nair Bindhu, A. Nayak, P. Saha, N. Sur, S. K. Swain, D. Vats, A. Alpana, S. Dube, B. Kansal, A. Laha, S. Pandey, A. Rastogi, S. Sharma, H. Bakhshiansohi, E. Khazaie, M. Zeinali, S. Chenarani, S. M. Etesami, M. Khakzad, M. Mohammadi Najafabadi, M. Grunewald, M. Abbrescia, R. Aly, C. Aruta, A. Colaleo, D. Creanza, N. De Filippis, M. De Palma, A. Di Florio, A. Di Pilato, W. Elmetenawee, F. Errico, L. Fiore, A. Gelmi, M. Gul, G. Iaselli, M. Ince, S. Lezki, G. Maggi, M. Maggi, I. Margjeka, V. Mastrapasqua, S. My, S. Nuzzo, A. Pellecchia, A. Pompili, G. Pugliese, D. Ramos, A. Ranieri, G. Selvaggi, L. Silvestris, F. M. Simone, Ü. Sözbilir, R. Venditti, P. Verwilligen, G. Abbiendi, C. Battilana, D. Bonacorsi, L. Borgonovi, L. Brigliadori, R. Campanini, P. Capiluppi, A. Castro, F. R. Cavallo, C. Ciocca, M. Cuffiani, G. M. Dallavalle, T. Diotalevi, F. Fabbri, A. Fanfani, P. Giacomelli, L. Giommi, C. Grandi, L. Guiducci, S. Lo Meo, L. Lunerti, S. Marcellini, G. Masetti, F. L. Navarria, A. Perrottas, F. Primavera, A. M. Rossi, T. Rovelli, G. P. Siroli, S. Albergo, S. Costa, A. Di Mattia, R. Potenza, A. Tricomi, C. Tuve, G. Barbagli, A. Cassese, R. Ceccarelli, V. Ciulli, C. Civinini, R. D’Alessandro, E. Focardi, G. Latino, P. Lenzi, M. Lizzo, M. Meschini, S. Paoletti, R. Seidita, G. Sguazzoni, L. Viliani, L. Benussi, S. Bianco, D. Piccolo, M. Bozzo, F. Ferro, R. Mulargia, E. Robutti, S. Tosi, A. Benaglia, G. Boldrini, F. Brivio, F. Cetorelli, F. De Guio, M. E. Dinardo, P. Dini, S. Gennai, A. Ghezzi, P. Govoni, L. Guzzi, M. T. Lucchini, M. Malberti, S. Malvezzi, A. Massironi, D. Menasce, L. Moroni, M. Paganoni, D. Pedrini, B. S. Pinolini, S. Ragazzi, N. Redaelli, T. Tabarelli de Fatis, D. Valsecchi, D. Zuolo, S. Buontempo, F. Carnevali, N. Cavallo, A. De Iorio, F. Fabozzi, A. O. M. Iorio, L. Lista, S. Meola, P. Paolucci, B. Rossi, C. Sciacca, P. Azzi, N. Bacchetta, D. Bisello, P. Bortignon, A. Bragagnolo, R. Carlin, P. Checchia, T. Dorigo, U. Dosselli, F. Gasparini, U. Gasparini, G. Grosso, S. Y. Hoh, L. Layer, E. Lusiani, M. Margoni, A. T. Meneguzzo, J. Pazzini, P. Ronchese, R. Rossin, F. Simonetto, G. Strong, M. Tosi, H. Yarar, M. Zanetti, P. Zotto, A. Zucchetta, G. Zumerle, C. Aimè, A. Braghieri, S. Calzaferri, D. Fiorina, P. Montagna, S. P. Ratti, V. Re, C. Riccardi, P. Salvini, I. Vai, P. Vitulo, P. Asenov, G. M. Bilei, D. Ciangottini, L. Fanò, M. Magherini, G. Mantovani, V. Mariani, M. Menichelli, F. Moscatelli, A. Piccinelli, M. Presilla, A. Rossi, A. Santocchia, D. Spiga, T. Tedeschi, P. Azzurri, G. Bagliesi, V. Bertacchi, L. Bianchini, T. Boccali, E. Bossini, R. Castaldi, M. A. Ciocci, V. D’Amante, R. Dell’Orso, M. R. Di Domenico, S. Donato, A. Giassi, F. Ligabue, E. Manca, G. Mandorli, D. Matos Figueiredo, A. Messineo, M. Musich, F. Palla, S. Parolia, G. Ramirez-Sanchez, A. Rizzi, G. Rolandi, S. Roy Chowdhury, A. Scribano, N. Shafiei, P. Spagnolo, R. Tenchini, G. Tonelli, N. Turini, A. Venturi, P. G. Verdini, P. Barria, M. Campana, F. Cavallari, D. Del Re, E. Di Marco, M. Diemoz, E. Longo, P. Meridiani, G. Organtini, F. Pandolfi, R. Paramatti, C. Quaranta, S. Rahatlou, C. Rovelli, F. Santanastasio, L. Soffi, R. Tramontano, N. Amapane, R. Arcidiacono, S. Argiro, M. Arneodo, N. Bartosik, R. Bellan, A. Bellora, J. Berenguer Antequera, C. Biino, N. Cartiglia, M. Costa, R. Covarelli, N. Demaria, B. Kiani, F. Legger, C. Mariotti, S. Maselli, E. Migliore, E. Monteil, M. Monteno, M. M. Obertino, G. Ortona, L. Pacher, N. Pastrone, M. Pelliccioni, M. Ruspa, K. Shchelina, F. Siviero, V. Sola, A. Solano, D. Soldi, A. Staiano, M. Tornago, D. Trocino, A. Vagnerini, S. Belforte, V. Candelise, M. Casarsa, F. Cossutti, A. Da Rold, G. Della Ricca, G. Sorrentino, S. Dogra, C. Huh, B. Kim, D. H. Kim, G. N. Kim, J. Kim, J. Lee, S. W. Lee, C. S. Moon, Y. D. Oh, S. I. Pak, S. Sekmen, Y. C. Yang, H. Kim, D. H. Moon, B. Francois, T. J. Kim, J. Park, S. Cho, S. Choi, B. Hong, K. Lee, K. S. Lee, J. Lim, J. Park, S. K. Park, J. Yoo, J. Goh, A. Gurtu, H. S. Kim, Y. Kim, J. Almond, J. H. Bhyun, J. Choi, S. Jeon, J. Kim, J. S. Kim, S. Ko, H. Kwon, H. Lee, S. Lee, B. H. Oh, M. Oh, S. B. Oh, H. Seo, U. K. Yang, I. Yoon, W. Jang, D. Y. Kang, Y. Kang, S. Kim, B. Ko, J. S. H. Lee, Y. Lee, J. A. Merlin, I. C. Park, Y. Roh, M. S. Ryu, D. Song, I. J. Watson, S. Yang, S. Ha, H. D. Yoo, M. Choi, H. Lee, Y. Lee, I. Yu, T. Beyrouthy, Y. Maghrbi, K. Dreimanis, V. Veckalns, M. Ambrozas, A. Carvalho Antunes De Oliveira, A. Juodagalvis, A. Rinkevicius, G. Tamulaitis, N. Bin Norjoharuddeen, Z. Zolkapli, J. F. Benitez, A. Castaneda Hernandez, M. León Coello, J. A. Murillo Quijada, A. Sehrawat, L. Valencia Palomo, G. Ayala, H. Castilla-Valdez, E. De La Cruz-Burelo, I. Heredia-De La Cruz, R. Lopez-Fernandez, C. A. Mondragon Herrera, D. A. Perez Navarro, R. Reyes-Almanza, A. Sánchez Hernández, S. Carrillo Moreno, C. Oropeza Barrera, F. Vazquez Valencia, I. Pedraza, H. A. Salazar Ibarguen, C. Uribe Estrada, J. Mijuskovic, N. Raicevic, D. Krofcheck, P. H. Butler, A. Ahmad, M. I. Asghar, A. Awais, M. I. M. Awan, H. R. Hoorani, W. A. Khan, M. A. Shah, M. Shoaib, M. Waqas, V. Avati, L. Grzanka, M. Malawski, H. Bialkowska, M. Bluj, B. Boimska, M. Górski, M. Kazana, M. Szleper, P. Zalewski, K. Bunkowski, K. Doroba, A. Kalinowski, M. Konecki, J. Krolikowski, M. Araujo, P. Bargassa, D. Bastos, A. Boletti, P. Faccioli, M. Gallinaro, J. Hollar, N. Leonardo, T. Niknejad, M. Pisano, J. Seixas, O. Toldaiev, J. Varela, P. Adzic, M. Dordevic, P. Milenovic, J. Milosevic, M. Aguilar-Benitez, J. Alcaraz Maestre, A. Álvarez Fernández, I. Bachiller, M. Barrio Luna, Cristina F. Bedoya, C. A. Carrillo Montoya, M. Cepeda, M. Cerrada, N. Colino, B. De La Cruz, A. Delgado Peris, J. P. Fernández Ramos, J. Flix, M. C. Fouz, O. Gonzalez Lopez, S. Goy Lopez, J. M. Hernandez, M. I. Josa, J. León Holgado, D. Moran, Á. Navarro Tobar, C. Perez Dengra, A. Pérez-Calero Yzquierdo, J. Puerta Pelayo, I. Redondo, L. Romero, S. Sánchez Navas, L. Urda Gómez, C. Willmott, J. F. de Trocóniz, B. Alvarez Gonzalez, J. Cuevas, C. Erice, J. Fernandez Menendez, S. Folgueras, I. Gonzalez Caballero, J. R. González Fernández, E. Palencia Cortezon, C. Ramón Álvarez, V. Rodríguez Bouza, A. Soto Rodríguez, A. Trapote, N. Trevisani, C. Vico Villalba, J. A. Brochero Cifuentes, I. J. Cabrillo, A. Calderon, J. Duarte Campderros, M. Fernandez, C. Fernandez Madrazo, P. J. Fernández Manteca, A. García Alonso, G. Gomez, C. Martinez Rivero, P. Martinez Ruiz del Arbol, F. Matorras, P. Matorras Cuevas, J. Piedra Gomez, C. Prieels, A. Ruiz-Jimeno, L. Scodellaro, I. Vila, J. M. Vizan Garcia, M. K. Jayananda, B. Kailasapathy, D. U. J. Sonnadara, D. D. C. Wickramarathna, W. G. D. Dharmaratna, K. Liyanage, N. Perera, N. Wickramage, T. K. Aarrestad, D. Abbaneo, J. Alimena, E. Auffray, G. Auzinger, J. Baechler, P. Baillon, D. Barney, J. Bendavid, M. Bianco, A. Bocci, C. Caillol, T. Camporesi, M. Capeans Garrido, G. Cerminara, N. Chernyavskaya, S. S. Chhibra, S. Choudhury, M. Cipriani, L. Cristella, D. d’Enterria, A. Dabrowski, A. David, A. De Roeck, M. M. Defranchis, M. Deile, M. Dobson, M. Dünser, N. Dupont, A. Elliott-Peisert, N. Emriskova, F. Fallavollita, A. Florent, L. Forthomme, G. Franzoni, W. Funk, S. Ghosh, S. Giani, D. Gigi, K. Gill, F. Glege, L. Gouskos, M. Haranko, J. Hegeman, V. Innocente, T. James, P. Janot, J. Kaspar, J. Kieseler, M. Komm, N. Kratochwil, C. Lange, S. Laurila, P. Lecoq, A. Lintuluoto, K. Long, C. Lourenço, B. Maier, L. Malgeri, S. Mallios, M. Mannelli, A. C. Marini, F. Meijers, S. Mersi, E. Meschi, F. Moortgat, M. Mulders, S. Orfanelli, L. Orsini, F. Pantaleo, E. Perez, M. Peruzzi, A. Petrilli, G. Petrucciani, A. Pfeiffer, M. Pierini, D. Piparo, M. Pitt, H. Qu, T. Quast, D. Rabady, A. Racz, G. Reales Gutiérrez, M. Rovere, H. Sakulin, J. Salfeld-Nebgen, S. Scarfi, C. Schäfer, C. Schwick, M. Selvaggi, A. Sharma, P. Silva, W. Snoeys, P. Sphicas, S. Summers, K. Tatar, V. R. Tavolaro, D. Treille, P. Tropea, A. Tsirou, J. Wanczyk, K. A. Wozniak, W. D. Zeuner, L. Caminada, A. Ebrahimi, W. Erdmann, R. Horisberger, Q. Ingram, H. C. Kaestli, D. Kotlinski, M. Missiroli, L. Noehte, T. Rohe, K. Androsov, M. Backhaus, P. Berger, A. Calandri, A. De Cosa, G. Dissertori, M. Dittmar, M. Donegà, C. Dorfer, F. Eble, K. Gedia, F. Glessgen, T. A. Gómez Espinosa, C. Grab, D. Hits, W. Lustermann, A.-M. Lyon, R. A. Manzoni, L. Marchese, C. Martin Perez, M. T. Meinhard, F. Nessi-Tedaldi, J. Niedziela, F. Pauss, V. Perovic, S. Pigazzini, M. G. Ratti, M. Reichmann, C. Reissel, T. Reitenspiess, B. Ristic, D. Ruini, D. A. Sanz Becerra, V. Stampf, J. Steggemann, R. Wallny, C. Amsler, P. Bärtschi, C. Botta, D. Brzhechko, M. F. Canelli, K. Cormier, A. De Wit, R. Del Burgo, J. K. Heikkilä, M. Huwiler, W. Jin, A. Jofrehei, B. Kilminster, S. Leontsinis, S. P. Liechti, A. Macchiolo, P. Meiring, V. M. Mikuni, U. Molinatti, I. Neutelings, A. Reimers, P. Robmann, S. Sanchez Cruz, K. Schweiger, M. Senger, Y. Takahashi, C. Adloff, C. M. Kuo, W. Lin, A. Roy, T. Sarkar, S. S. Yu, L. Ceard, Y. Chao, K. F. Chen, P. H. Chen, P. s. Chen, H. Cheng, W.-S. Hou, Y.y. Li, R.-S. Lu, E. Paganis, A. Psallidas, A. Steen, H. y. Wu, E. Yazgan, P. r. Yu, B. Asavapibhop, C. Asawatangtrakuldee, N. Srimanobhas, F. Boran, S. Damarseckin, Z. S. Demiroglu, F. Dolek, I. Dumanoglu, E. Eskut, Y. Guler, E. Gurpinar Guler, C. Isik, O. Kara, A. Kayis Topaksu, U. Kiminsu, G. Onengut, K. Ozdemir, A. Polatoz, A. E. Simsek, B. Tali, U. G. Tok, S. Turkcapar, I. S. Zorbakir, G. Karapinar, K. Ocalan, M. Yalvac, B. Akgun, I. O. Atakisi, E. Gülmez, M. Kaya, O. Kaya, Ö. Özçelik, S. Tekten, E. A. Yetkin, A. Cakir, K. Cankocak, Y. Komurcu, S. Sen, S. Cerci, I. Hos, B. Isildak, B. Kaynak, S. Ozkorucuklu, H. Sert, D. Sunar Cerci, C. Zorbilmez, B. Grynyov, L. Levchuk, D. Anthony, E. Bhal, S. Bologna, J. J. Brooke, A. Bundock, E. Clement, D. Cussans, H. Flacher, J. Goldstein, G. P. Heath, H. F. Heath, L. Kreczko, B. Krikler, S. Paramesvaran, S. Seif El Nasr-Storey, V. J. Smith, N. Stylianou, K. Walkingshaw Pass, R. White, K. W. Bell, A. Belyaev, C. Brew, R. M. Brown, D. J. A. Cockerill, C. Cooke, K. V. Ellis, K. Harder, S. Harper, M.-L. Holmberg, J. Linacre, K. Manolopoulos, D. M. Newbold, E. Olaiya, D. Petyt, T. Reis, T. Schuh, C. H. Shepherd-Themistocleous, I. R. Tomalin, T. Williams, R. Bainbridge, P. Bloch, S. Bonomally, J. Borg, S. Breeze, O. Buchmuller, V. Cepaitis, G. S. Chahal, D. Colling, P. Dauncey, G. Davies, M. Della Negra, S. Fayer, G. Fedi, G. Hall, M. H. Hassanshahi, G. Iles, J. Langford, L. Lyons, A.-M. Magnan, S. Malik, A. Martelli, D. G. Monk, J. Nash, M. Pesaresi, B. C. Radburn-Smith, D. M. Raymond, A. Richards, A. Rose, E. Scott, C. Seez, A. Shtipliyski, A. Tapper, K. Uchida, T. Virdee, M. Vojinovic, N. Wardle, S. N. Webb, D. Winterbottom, K. Coldham, J. E. Cole, A. Khan, P. Kyberd, I. D. Reid, L. Teodorescu, S. Zahid, S. Abdullin, A. Brinkerhoff, B. Caraway, J. Dittmann, K. Hatakeyama, A. R. Kanuganti, B. McMaster, N. Pastika, M. Saunders, S. Sawant, C. Sutantawibul, J. Wilson, R. Bartek, A. Dominguez, R. Uniyal, A. M. Vargas Hernandez, A. Buccilli, S. I. Cooper, D. Di Croce, S. V. Gleyzer, C. Henderson, C. U. Perez, P. Rumerio, C. West, A. Akpinar, A. Albert, D. Arcaro, C. Cosby, Z. Demiragli, E. Fontanesi, D. Gastler, S. May, J. Rohlf, K. Salyer, D. Sperka, D. Spitzbart, I. Suarez, A. Tsatsos, S. Yuan, D. Zou, G. Benelli, B. Burkle, X. Coubez, D. Cutts, M. Hadley, U. Heintz, J. M. Hogan, T. Kwon, G. Landsberg, K. T. Lau, D. Li, M. Lukasik, J. Luo, M. Narain, N. Pervan, S. Sagir, F. Simpson, E. Usai, W. Y. Wong, X. Yan, D. Yu, W. Zhang, J. Bonilla, C. Brainerd, R. Breedon, M. Calderon De La Barca Sanchez, M. Chertok, J. Conway, P. T. Cox, R. Erbacher, G. Haza, F. Jensen, O. Kukral, R. Lander, M. Mulhearn, D. Pellett, B. Regnery, D. Taylor, Y. Yao, F. Zhang, M. Bachtis, R. Cousins, A. Datta, D. Hamilton, J. Hauser, M. Ignatenko, M. A. Iqbal, T. Lam, W. A. Nash, S. Regnard, D. Saltzberg, B. Stone, V. Valuev, Y. Chen, R. Clare, J. W. Gary, M. Gordon, G. Hanson, G. Karapostoli, O. R. Long, N. Manganelli, W. Si, S. Wimpenny, Y. Zhang, J. G. Branson, P. Chang, S. Cittolin, S. Cooperstein, N. Deelen, D. Diaz, J. Duarte, R. Gerosa, L. Giannini, J. Guiang, R. Kansal, V. Krutelyov, R. Lee, J. Letts, M. Masciovecchio, F. Mokhtar, M. Pieri, B. V. Sathia Narayanan, V. Sharma, M. Tadel, F. Würthwein, Y. Xiang, A. Yagil, N. Amin, C. Campagnari, M. Citron, A. Dorsett, V. Dutta, J. Incandela, M. Kilpatrick, J. Kim, B. Marsh, H. Mei, M. Oshiro, M. Quinnan, J. Richman, U. Sarica, F. Setti, J. Sheplock, P. Siddireddy, D. Stuart, S. Wang, A. Bornheim, O. Cerri, I. Dutta, J. M. Lawhorn, N. Lu, J. Mao, H. B. Newman, T. Q. Nguyen, M. Spiropulu, J. R. Vlimant, C. Wang, S. Xie, Z. Zhang, R. Y. Zhu, J. Alison, S. An, M. B. Andrews, P. Bryant, T. Ferguson, A. Harilal, C. Liu, T. Mudholkar, M. Paulini, A. Sanchez, W. Terrill, J. P. Cumalat, W. T. Ford, A. Hassani, G. Karathanasis, E. MacDonald, R. Patel, A. Perloff, C. Savard, N. Schonbeck, K. Stenson, K. A. Ulmer, S. R. Wagner, N. Zipper, J. Alexander, S. Bright-Thonney, X. Chen, Y. Cheng, D. J. Cranshaw, S. Hogan, J. Monroy, J. R. Patterson, D. Quach, J. Reichert, M. Reid, A. Ryd, W. Sun, J. Thom, P. Wittich, R. Zou, M. Albrow, M. Alyari, G. Apollinari, A. Apresyan, A. Apyan, L. A. T. Bauerdick, D. Berry, J. Berryhill, P. C. Bhat, K. Burkett, J. N. Butler, A. Canepa, G. B. Cerati, H. W. K. Cheung, F. Chlebana, K. F. Di Petrillo, V. D. Elvira, Y. Feng, J. Freeman, Z. Gecse, L. Gray, D. Green, S. Grünendahl, O. Gutsche, R. M. Harris, R. Heller, T. C. Herwig, J. Hirschauer, B. Jayatilaka, S. Jindariani, M. Johnson, U. Joshi, T. Klijnsma, B. Klima, K. H. M. Kwok, S. Lammel, D. Lincoln, R. Lipton, T. Liu, C. Madrid, K. Maeshima, C. Mantilla, D. Mason, P. McBride, P. Merkel, S. Mrenna, S. Nahn, J. Ngadiuba, V. Papadimitriou, K. Pedro, C. Pena, F. Ravera, A. Reinsvold Hall, L. Ristori, E. Sexton-Kennedy, N. Smith, A. Soha, L. Spiegel, J. Strait, L. Taylor, S. Tkaczyk, N. V. Tran, L. Uplegger, E. W. Vaandering, H. A. Weber, P. Avery, D. Bourilkov, L. Cadamuro, V. Cherepanov, R. D. Field, D. Guerrero, B. M. Joshi, M. Kim, E. Koenig, J. Konigsberg, A. Korytov, K. H. Lo, K. Matchev, N. Menendez, G. Mitselmakher, A. Muthirakalayil Madhu, N. Rawal, D. Rosenzweig, S. Rosenzweig, K. Shi, J. Wang, Z. Wu, E. Yigitbasi, X. Zuo, T. Adams, A. Askew, R. Habibullah, V. Hagopian, K. F. Johnson, R. Khurana, T. Kolberg, G. Martinez, H. Prosper, C. Schiber, O. Viazlo, R. Yohay, J. Zhang, M. M. Baarmand, S. Butalla, T. Elkafrawy, M. Hohlmann, R. Kumar Verma, D. Noonan, M. Rahmani, F. Yumiceva, M. R. Adams, H. Becerril Gonzalez, R. Cavanaugh, S. Dittmer, O. Evdokimov, C. E. Gerber, D. J. Hofman, A. H. Merrit, C. Mills, G. Oh, T. Roy, S. Rudrabhatla, M. B. Tonjes, N. Varelas, J. Viinikainen, X. Wang, Z. Ye, M. Alhusseini, K. Dilsiz, L. Emediato, R. P. Gandrajula, O. K. Köseyan, J.-P. Merlo, A. Mestvirishvili, J. Nachtman, H. Ogul, Y. Onel, A. Penzo, C. Snyder, E. Tiras, O. Amram, B. Blumenfeld, L. Corcodilos, J. Davis, A. V. Gritsan, S. Kyriacou, P. Maksimovic, J. Roskes, M. Swartz, T.Á. Vámi, A. Abreu, J. Anguiano, C. Baldenegro Barrera, P. Baringer, A. Bean, A. Bylinkin, Z. Flowers, T. Isidori, S. Khalil, J. King, G. Krintiras, A. Kropivnitskaya, M. Lazarovits, C. Le Mahieu, C. Lindsey, J. Marquez, N. Minafra, M. Murray, M. Nickel, C. Rogan, C. Royon, R. Salvatico, S. Sanders, E. Schmitz, C. Smith, Q. Wang, Z. Warner, J. Williams, G. Wilson, S. Duric, A. Ivanov, K. Kaadze, D. Kim, Y. Maravin, T. Mitchell, A. Modak, K. Nam, F. Rebassoo, D. Wright, E. Adams, A. Baden, O. Baron, A. Belloni, S. C. Eno, N. J. Hadley, S. Jabeen, R. G. Kellogg, T. Koeth, Y. Lai, S. Lascio, A. C. Mignerey, S. Nabili, C. Palmer, M. Seidel, A. Skuja, L. Wang, K. Wong, D. Abercrombie, G. Andreassi, R. Bi, W. Busza, I. A. Cali, Y. Chen, M. D’Alfonso, J. Eysermans, C. Freer, G. Gomez-Ceballos, M. Goncharov, P. Harris, M. Hu, M. Klute, D. Kovalskyi, J. Krupa, Y.-J. Lee, C. Mironov, C. Paus, D. Rankin, C. Roland, G. Roland, Z. Shi, G. S. F. Stephans, J. Wang, Z. Wang, B. Wyslouch, R. M. Chatterjee, A. Evans, J. Hiltbrand, Sh. Jain, M. Krohn, Y. Kubota, J. Mans, M. Revering, R. Rusack, R. Saradhy, N. Schroeder, N. Strobbe, M. A. Wadud, K. Bloom, M. Bryson, S. Chauhan, D. R. Claes, C. Fangmeier, L. Finco, F. Golf, C. Joo, I. Kravchenko, I. Reed, J. E. Siado, G. R. Snow, W. Tabb, A. Wightman, F. Yan, A. G. Zecchinelli, G. Agarwal, H. Bandyopadhyay, L. Hay, I. Iashvili, A. Kharchilava, C. McLean, D. Nguyen, J. Pekkanen, S. Rappoccio, A. Williams, G. Alverson, E. Barberis, Y. Haddad, Y. Han, A. Hortiangtham, A. Krishna, J. Li, J. Lidrych, G. Madigan, B. Marzocchi, D. M. Morse, V. Nguyen, T. Orimoto, A. Parker, L. Skinnari, A. Tishelman-Charny, T. Wamorkar, B. Wang, A. Wisecarver, D. Wood, S. Bhattacharya, J. Bueghly, Z. Chen, A. Gilbert, T. Gunter, K. A. Hahn, Y. Liu, N. Odell, M. H. Schmitt, M. Velasco, R. Band, R. Bucci, M. Cremonesi, A. Das, N. Dev, R. Goldouzian, M. Hildreth, K. Hurtado Anampa, C. Jessop, K. Lannon, J. Lawrence, N. Loukas, L. Lutton, J. Mariano, N. Marinelli, I. Mcalister, T. McCauley, C. Mcgrady, K. Mohrman, C. Moore, Y. Musienko, R. Ruchti, A. Townsend, M. Wayne, M. Zarucki, L. Zygala, B. Bylsma, L. S. Durkin, B. Francis, C. Hill, M. Nunez Ornelas, K. Wei, B. L. Winer, B. R. Yates, F. M. Addesa, B. Bonham, P. Das, G. Dezoort, P. Elmer, A. Frankenthal, B. Greenberg, N. Haubrich, S. Higginbotham, A. Kalogeropoulos, G. Kopp, S. Kwan, D. Lange, D. Marlow, K. Mei, I. Ojalvo, J. Olsen, D. Stickland, C. Tully, S. Malik, S. Norberg, A. S. Bakshi, V. E. Barnes, R. Chawla, S. Das, L. Gutay, M. Jones, A. W. Jung, D. Kondratyev, A. M. Koshy, M. Liu, G. Negro, N. Neumeister, G. Paspalaki, S. Piperov, A. Purohit, J. F. Schulte, M. Stojanovic, J. Thieman, F. Wang, R. Xiao, W. Xie, J. Dolen, N. Parashar, D. Acosta, A. Baty, T. Carnahan, M. Decaro, S. Dildick, K. M. Ecklund, S. Freed, P. Gardner, F. J. M. Geurts, A. Kumar, W. Li, B. P. Padley, R. Redjimi, J. Rotter, W. Shi, A. G. Stahl Leiton, S. Yang, L. Zhang, Y. Zhang, A. Bodek, P. de Barbaro, R. Demina, J. L. Dulemba, C. Fallon, T. Ferbel, M. Galanti, A. Garcia-Bellido, O. Hindrichs, A. Khukhunaishvili, E. Ranken, R. Taus, G. P. Van Onsem, B. Chiarito, J. P. Chou, A. Gandrakota, Y. Gershtein, E. Halkiadakis, A. Hart, M. Heindl, O. Karacheban, I. Laflotte, A. Lath, R. Montalvo, K. Nash, M. Osherson, S. Salur, S. Schnetzer, S. Somalwar, R. Stone, S. A. Thayil, S. Thomas, H. Wang, H. Acharya, A. G. Delannoy, S. Fiorendi, S. Spanier, O. Bouhali, M. Dalchenko, A. Delgado, R. Eusebi, J. Gilmore, T. Huang, T. Kamon, H. Kim, S. Luo, S. Malhotra, R. Mueller, D. Overton, D. Rathjens, A. Safonov, N. Akchurin, J. Damgov, V. Hegde, S. Kunori, K. Lamichhane, S. W. Lee, T. Mengke, S. Muthumuni, T. Peltola, I. Volobouev, Z. Wang, A. Whitbeck, E. Appelt, S. Greene, A. Gurrola, W. Johns, A. Melo, K. Padeken, F. Romeo, P. Sheldon, S. Tuo, J. Velkovska, M. W. Arenton, B. Cardwell, B. Cox, G. Cummings, J. Hakala, R. Hirosky, M. Joyce, A. Ledovskoy, A. Li, C. Neu, C. E. Perez Lara, B. Tannenwald, S. White, N. Poudyal, S. Banerjee, K. Black, T. Bose, S. Dasu, I. De Bruyn, P. Everaerts, C. Galloni, H. He, M. Herndon, A. Hervé, U. Hussain, A. Lanaro, A. Loeliger, R. Loveless, J. Madhusudanan Sreekala, A. Mallampalli, A. Mohammadi, D. Pinna, A. Savin, V. Shang, V. Sharma, W. H. Smith, D. Teague, S. Trembath-Reichert, W. Vetens, S. Afanasiev, V. Andreev, Yu. Andreev, T. Aushev, M. Azarkin, A. Babaev, A. Belyaev, V. Blinov, E. Boos, V. Borshch, D. Budkouski, M. Chadeeva, V. Chekhovsky, A. Dermenev, T. Dimova, I. Dremin, M. Dubinin, L. Dudko, V. Epshteyn, A. Ershov, G. Gavrilov, V. Gavrilov, S. Gninenko, V. Golovtcov, N. Golubev, I. Golutvin, I. Gorbunov, A. Gribushin, V. Ivanchenko, Y. Ivanov, V. Kachanov, L. Kardapoltsev, V. Karjavine, A. Karneyeu, V. Kim, M. Kirakosyan, D. Kirpichnikov, M. Kirsanov, V. Klyukhin, O. Kodolova, D. Konstantinov, V. Korenkov, A. Kozyrev, N. Krasnikov, E. Kuznetsova, A. Lanev, A. Litomin, N. Lychkovskaya, V. Makarenko, A. Malakhov, V. Matveev, V. Murzin, A. Nikitenko, S. Obraztsov, V. Okhotnikov, V. Oreshkin, A. Oskin, I. Ovtin, V. Palichik, P. Parygin, A. Pashenkov, V. Perelygin, S. Petrushanko, G. Pivovarov, S. Polikarpov, V. Popov, O. Radchenko, M. Savina, V. Savrin, D. Selivanova, V. Shalaev, S. Shmatov, S. Shulha, Y. Skovpen, S. Slabospitskii, I. Smirnov, V. Smirnov, A. Snigirev, D. Sosnov, A. Stepennov, V. Sulimov, E. Tcherniaev, A. Terkulov, O. Teryaev, M. Toms, A. Toropin, L. Uvarov, A. Uzunian, E. Vlasov, S. Volkov, A. Vorobyev, N. Voytishin, B. S. Yuldashev, A. Zarubin, E. Zhemchugov, I. Zhizhin, A. Zhokin, CMS Collaboration

**Affiliations:** 1 Yerevan Physics Institute, Yerevan, Armenia; 2 Institut für Hochenergiephysik, Vienna, Austria; 3 Universiteit Antwerpen, Antwerpen, Belgium; 4 Vrije Universiteit Brussel, Brussels, Belgium; 5 Université Libre de Bruxelles, Brussels, Belgium; 6 Ghent University, Ghent, Belgium; 7 Université Catholique de Louvain, Louvain-la-Neuve, Belgium; 8 Centro Brasileiro de Pesquisas Fisicas, Rio de Janeiro, Brazil; 9 Universidade do Estado do Rio de Janeiro, Rio de Janeiro, Brazil; 10 Universidade Estadual Paulista, Universidade Federal do ABC, São Paulo, Brazil; 11 Institute for Nuclear Research and Nuclear Energy, Bulgarian Academy of Sciences, Sofia, Bulgaria; 12 University of Sofia, Sofia, Bulgaria; 13 Beihang University, Beijing, China; 14 Department of Physics, Tsinghua University, Beijing, China; 15 Institute of High Energy Physics, Beijing, China; 16 State Key Laboratory of Nuclear Physics and Technology, Peking University, Beijing, China; 17 Sun Yat-Sen University, Guangzhou, China; 18 Institute of Modern Physics and Key Laboratory of Nuclear Physics and Ion-beam Application (MOE), Fudan University, Shanghai, China; 19 Zhejiang University, Hangzhou, Zhejiang China; 20 Universidad de Los Andes, Bogota, Colombia; 21 Universidad de Antioquia, Medellin, Colombia; 22 Faculty of Electrical Engineering, Mechanical Engineering and Naval Architecture, University of Split, Split, Croatia; 23 Faculty of Science, University of Split, Split, Croatia; 24 Institute Rudjer Boskovic, Zagreb, Croatia; 25 University of Cyprus, Nicosia, Cyprus; 26 Charles University, Prague, Czech Republic; 27 Escuela Politecnica Nacional, Quito, Ecuador; 28 Universidad San Francisco de Quito, Quito, Ecuador; 29 Academy of Scientific Research and Technology of the Arab Republic of Egypt, Egyptian Network of High Energy Physics, Cairo, Egypt; 30 Center for High Energy Physics (CHEP-FU), Fayoum University, El-Fayoum, Egypt; 31 National Institute of Chemical Physics and Biophysics, Tallinn, Estonia; 32 Department of Physics, University of Helsinki, Helsinki, Finland; 33 Helsinki Institute of Physics, Helsinki, Finland; 34 Lappeenranta-Lahti University of Technology, Lappeenranta, Finland; 35 IRFU, CEA, Université Paris-Saclay, Gif-sur-Yvette, France; 36 Laboratoire Leprince-Ringuet, CNRS/IN2P3, Ecole Polytechnique, Institut Polytechnique de Paris, Palaiseau, France; 37 Université de Strasbourg, CNRS, IPHC UMR 7178, Strasbourg, France; 38 Institut de Physique des 2 Infinis de Lyon (IP2I ), Villeurbanne, France; 39 Georgian Technical University, Tbilisi, Georgia; 40 I. Physikalisches Institut, RWTH Aachen University, Aachen, Germany; 41 III. Physikalisches Institut A, RWTH Aachen University, Aachen, Germany; 42 III. Physikalisches Institut B, RWTH Aachen University, Aachen, Germany; 43 Deutsches Elektronen-Synchrotron, Hamburg, Germany; 44 University of Hamburg, Hamburg, Germany; 45 Karlsruher Institut fuer Technologie, Karlsruhe, Germany; 46 Institute of Nuclear and Particle Physics (INPP), NCSR Demokritos, Aghia Paraskevi, Greece; 47 National and Kapodistrian University of Athens, Athens, Greece; 48 National Technical University of Athens, Athens, Greece; 49 University of Ioánnina, Ioánnina, Greece; 50 MTA-ELTE Lendület CMS Particle and Nuclear Physics Group, Eötvös Loránd University, Budapest, Hungary; 51 Wigner Research Centre for Physics, Budapest, Hungary; 52 Institute of Nuclear Research ATOMKI, Debrecen, Hungary; 53 Institute of Physics, University of Debrecen, Debrecen, Hungary; 54 Karoly Robert Campus, MATE Institute of Technology, Gyongyos, Hungary; 55 Panjab University, Chandigarh, India; 56 University of Delhi, Delhi, India; 57 Saha Institute of Nuclear Physics, HBNI, Kolkata, India; 58 Indian Institute of Technology Madras, Madras, India; 59 Bhabha Atomic Research Centre, Mumbai, India; 60 Tata Institute of Fundamental Research-A, Mumbai, India; 61 Tata Institute of Fundamental Research-B, Mumbai, India; 62 National Institute of Science Education and Research, An OCC of Homi Bhabha National Institute, Bhubaneswar, Odisha India; 63 Indian Institute of Science Education and Research (IISER), Pune, India; 64 Isfahan University of Technology, Isfahan, Iran; 65 Institute for Research in Fundamental Sciences (IPM), Tehran, Iran; 66 University College Dublin, Dublin, Ireland; 67 INFN Sezione di Bari, Università di Bari, Politecnico di Bari, Bari, Italy; 68 INFN Sezione di Bologna, Università di Bologna, Bologna, Italy; 69 INFN Sezione di Catania, Università di Catania, Catania, Italy; 70 INFN Sezione di Firenze, Università di Firenze, Florence, Italy; 71 INFN Laboratori Nazionali di Frascati, Frascati, Italy; 72 INFN Sezione di Genova, Università di Genova, Genoa, Italy; 73 INFN Sezione di Milano-Bicocca, Università di Milano-Bicocca, Milan, Italy; 74 INFN Sezione di Napoli, Università di Napoli ’Federico II’, Napoli, Italy; Università della Basilicata, Potenza, Italy, Università G. Marconi, Rome, Italy; 75 INFN Sezione di Padova, Università di Padova, Padova, Italy; Università di Trento, Trento, Italy; 76 INFN Sezione di Pavia, Università di Pavia, Pavia, Italy; 77 INFN Sezione di Perugia, Università di Perugia, Perugia, Italy; 78 INFN Sezione di Pisa, Università di Pisa, Scuola Normale Superiore di Pisa, Pisa, Italy, Università di Siena, Siena, Italy; 79 INFN Sezione di Roma, Sapienza Università di Roma, Rome, Italy; 80 INFN Sezione di Torino, Università di Torino, Torino, Università del Piemonte Orientale, Novara, Italy; 81 INFN Sezione di Trieste, Università di Trieste, Trieste, Italy; 82 Kyungpook National University, Daegu, Korea; 83 Chonnam National University, Institute for Universe and Elementary Particles, Kwangju, Korea; 84 Hanyang University, Seoul, Korea; 85 Korea University, Seoul, Korea; 86 Department of Physics, Kyung Hee University, Seoul, Korea; 87 Sejong University, Seoul, Korea; 88 Seoul National University, Seoul, Korea; 89 University of Seoul, Seoul, Korea; 90 Department of Physics, Yonsei University, Seoul, Korea; 91 Sungkyunkwan University, Suwon, Korea; 92 College of Engineering and Technology, American University of the Middle East (AUM), Dasman, Kuwait; 93 Riga Technical University, Riga, Latvia; 94 Vilnius University, Vilnius, Lithuania; 95 National Centre for Particle Physics, Universiti Malaya, Kuala Lumpur, Malaysia; 96 Universidad de Sonora (UNISON), Hermosillo, Mexico; 97 Centro de Investigacion y de Estudios Avanzados del IPN, Mexico City, Mexico; 98 Universidad Iberoamericana, Mexico City, Mexico; 99 Benemerita Universidad Autonoma de Puebla, Puebla, Mexico; 100 University of Montenegro, Podgorica, Montenegro; 101 University of Auckland, Auckland, New Zealand; 102 University of Canterbury, Christchurch, New Zealand; 103 National Centre for Physics, Quaid-I-Azam University, Islamabad, Pakistan; 104 Faculty of Computer Science, Electronics and Telecommunications, AGH University of Science and Technology, Krakow, Poland; 105 National Centre for Nuclear Research, Swierk, Poland; 106 Institute of Experimental Physics, Faculty of Physics, University of Warsaw, Warsaw, Poland; 107 Laboratório de Instrumentação e Física Experimental de Partículas, Lisbon, Portugal; 108 VINCA Institute of Nuclear Sciences, University of Belgrade, Belgrade, Serbia; 109 Centro de Investigaciones Energéticas Medioambientales y Tecnológicas (CIEMAT), Madrid, Spain; 110 Universidad Autónoma de Madrid, Madrid, Spain; 111 Instituto Universitario de Ciencias y Tecnologías Espaciales de Asturias (ICTEA), Universidad de Oviedo, Oviedo, Spain; 112 Instituto de Física de Cantabria (IFCA), CSIC-Universidad de Cantabria, Santander, Spain; 113 University of Colombo, Colombo, Sri Lanka; 114 Department of Physics, University of Ruhuna, Matara, Sri Lanka; 115 CERN, European Organization for Nuclear Research, Geneva, Switzerland; 116 Paul Scherrer Institut, Villigen, Switzerland; 117 ETH Zurich-Institute for Particle Physics and Astrophysics (IPA), Zurich, Switzerland; 118 Universität Zürich, Zurich, Switzerland; 119 National Central University, Chung-Li, Taiwan; 120 National Taiwan University (NTU), Taipei, Taiwan; 121 Department of Physics, Faculty of Science, Chulalongkorn University, Bangkok, Thailand; 122 Physics Department, Science and Art Faculty, Çukurova University, Adana, Turkey; 123 Physics Department, Middle East Technical University, Ankara, Turkey; 124 Bogazici University, Istanbul, Turkey; 125 Istanbul Technical University, Istanbul, Turkey; 126 Istanbul University, Istanbul, Turkey; 127 Institute for Scintillation Materials of National Academy of Science of Ukraine, Kharkiv, Ukraine; 128 National Science Centre, Kharkiv Institute of Physics and Technology, Kharkiv, Ukraine; 129 University of Bristol, Bristol, UK; 130 Rutherford Appleton Laboratory, Didcot, UK; 131 Imperial College, London, UK; 132 Brunel University, Uxbridge, UK; 133 Baylor University, Waco, TX USA; 134 Catholic University of America, Washington, DC USA; 135 The University of Alabama, Tuscaloosa, AL USA; 136 Boston University, Boston, MA USA; 137 Brown University, Providence, RI USA; 138 University of California, Davis, Davis, CA USA; 139 University of California, Los Angeles, CA USA; 140 University of California, Riverside, Riverside, CA USA; 141 University of California, San Diego, La Jolla, CA USA; 142 Department of Physics, University of California, Santa Barbara, Santa Barbara, CA USA; 143 California Institute of Technology, Pasadena, CA USA; 144 Carnegie Mellon University, Pittsburgh, PA USA; 145 University of Colorado Boulder, Boulder, CO USA; 146 Cornell University, Ithaca, NY USA; 147 Fermi National Accelerator Laboratory, Batavia, IL USA; 148 University of Florida, Gainesville, FL USA; 149 Florida State University, Tallahassee, FL USA; 150 Florida Institute of Technology, Melbourne, FL USA; 151 University of Illinois at Chicago (UIC), Chicago, IL USA; 152 The University of Iowa, Iowa City, IA USA; 153 Johns Hopkins University, Baltimore, MD USA; 154 The University of Kansas, Lawrence, KS USA; 155 Kansas State University, Manhattan, KS USA; 156 Lawrence Livermore National Laboratory, Livermore, CA USA; 157 University of Maryland, College Park, MD USA; 158 Massachusetts Institute of Technology, Cambridge, MA USA; 159 University of Minnesota, Minneapolis, MN USA; 160 University of Nebraska-Lincoln, Lincoln, NE USA; 161 State University of New York at Buffalo, Buffalo, NY USA; 162 Northeastern University, Boston, MA USA; 163 Northwestern University, Evanston, IL USA; 164 University of Notre Dame, Notre Dame, IN USA; 165 The Ohio State University, Columbus, OH USA; 166 Princeton University, Princeton, NJ USA; 167 University of Puerto Rico, Mayaguez, PR USA; 168 Purdue University, West Lafayette, IN USA; 169 Purdue University Northwest, Hammond, IN USA; 170 Rice University, Houston, TX USA; 171 University of Rochester, Rochester, NY USA; 172 Rutgers, The State University of New Jersey, Piscataway, NJ USA; 173 University of Tennessee, Knoxville, TN USA; 174 Texas A &M University, College Station, TX USA; 175 Texas Tech University, Lubbock, Texas USA; 176 Vanderbilt University, Nashville, TN USA; 177 University of Virginia, Charlottesville, VI USA; 178 Wayne State University, Detroit, MI USA; 179 University of Wisconsin-Madison, Madison, WI USA; 180 Authors affiliated with an institute or an international laboratory covered by a cooperation agreement with CERN, Geneva, Switzerland; 181 Yerevan State University, Yerevan, Armenia; 182 TU Wien, Vienna, Austria; 183 Institute of Basic and Applied Sciences, Faculty of Engineering, Arab Academy for Science, Technology and Maritime Transport, Alexandria, Egypt; 184 Université Libre de Bruxelles, Brussels, Belgium; 185 Universidade Estadual de Campinas, Campinas, Brazil; 186 Federal University of Rio Grande do Sul, Porto Alegre, Brazil; 187 The University of the State of Amazonas, Manaus, Brazil; 188 University of Chinese Academy of Sciences, Beijing, China; 189 UFMS, Nova Andradina, Brazil; 190 Nanjing Normal University Department of Physics, Nanjing, China; 191 The University of Iowa, Iowa City, IA USA; 192 University of Chinese Academy of Sciences, Beijing, China; 193 an institute or an international laboratory covered by a cooperation agreement with CERN, Geneva, Switzerland; 194 Helwan University, Cairo, Egypt; 195 Zewail City of Science and Technology, Zewail, Egypt; 196 Purdue University, West Lafayette, IN USA; 197 Université de Haute Alsace, Mulhouse, France; 198 Tbilisi State University, Tbilisi, Georgia; 199 Erzincan Binali Yildirim University, Erzincan, Turkey; 200 CERN, European Organization for Nuclear Research, Geneva, Switzerland; 201 III. Physikalisches Institut A, RWTH Aachen University, Aachen, Germany; 202 University of Hamburg, Hamburg, Germany; 203 Isfahan University of Technology, Isfahan, Iran; 204 Brandenburg University of Technology, Cottbus, Germany; 205 Forschungszentrum Jülich, Jülich, Germany; 206 Physics Department, Faculty of Science, Assiut University, Assiut, Egypt; 207 Karoly Robert Campus, MATE Institute of Technology, Gyongyos, Hungary; 208 Institute of Physics, University of Debrecen, Debrecen, Hungary; 209 Institute of Nuclear Research ATOMKI, Debrecen, Hungary; 210 Universitatea Babes-Bolyai-Facultatea de Fizica, Cluj-Napoca, Romania; 211 MTA-ELTE Lendület CMS Particle and Nuclear Physics Group, Eötvös Loránd University, Budapest, Hungary; 212 Wigner Research Centre for Physics, Budapest, Hungary; 213 Punjab Agricultural University, Ludhiana, India; 214 Shoolini University, Solan, India; 215 University of Hyderabad, Hyderabad, India; 216 University of Visva-Bharati, Santiniketan, India; 217 Indian Institute of Science (IISc), Bangalore, India; 218 Indian Institute of Technology (IIT), Mumbai, India; 219 IIT Bhubaneswar, Bhubaneswar, India; 220 Institute of Physics, Bhubaneswar, India; 221 Deutsches Elektronen-Synchrotron, Hamburg, Germany; 222 Sharif University of Technology, Tehran, Iran; 223 Department of Physics, University of Science and Technology of Mazandaran, Behshahr, Iran; 224 Italian National Agency for New Technologies, Energy and Sustainable Economic Development, Bologna, Italy; 225 Centro Siciliano di Fisica Nucleare e di Struttura Della Materia, Catania, Italy; 226 Scuola Superiore Meridionale, Università di Napoli ’Federico II’, Naples, Italy; 227 Università di Napoli ’Federico II’, Naples, Italy; 228 Consiglio Nazionale delle Ricerche-Istituto Officina dei Materiali, Perugia, Italy; 229 Consejo Nacional de Ciencia y Tecnología, Mexico City, Mexico; 230 IRFU, CEA, Université Paris-Saclay, Gif-sur-Yvette, France; 231 Faculty of Physics, University of Belgrade, Belgrade, Serbia; 232 Trincomalee Campus, Eastern University, Sri Lanka, Nilaveli, Sri Lanka; 233 INFN Sezione di Pavia, Università di Pavia, Pavia, Italy; 234 National and Kapodistrian University of Athens, Athens, Greece; 235 Ecole Polytechnique Fédérale Lausanne, Lausanne, Switzerland; 236 Universität Zürich, Zurich, Switzerland; 237 Stefan Meyer Institute for Subatomic Physics, Vienna, Austria; 238 Laboratoire d’Annecy-le-Vieux de Physique des Particules, IN2P3-CNRS, Annecy-le-Vieux, France; 239 Şırnak University, Sirnak, Turkey; 240 Near East University, Research Center of Experimental Health Science, Mersin, Turkey; 241 Konya Technical University, Konya, Turkey; 242 Izmir Bakircay University, Izmir, Turkey; 243 Adiyaman University, Adiyaman, Turkey; 244 Necmettin Erbakan University, Konya, Turkey; 245 Bozok Universitetesi Rektörlügü, Yozgat, Turkey; 246 Marmara University, Istanbul, Turkey; 247 Milli Savunma University, Istanbul, Turkey; 248 Kafkas University, Kars, Turkey; 249 Istanbul Bilgi University, Istanbul, Turkey; 250 Hacettepe University, Ankara, Turkey; 251 Faculty of Engineering, Istanbul University-Cerrahpasa, Istanbul, Turkey; 252 Yildiz Technical University, Istanbul, Turkey; 253 Vrije Universiteit Brussel, Brussels, Belgium; 254 School of Physics and Astronomy, University of Southampton, Southampton, UK; 255 IPPP Durham University, Durham, UK; 256 Faculty of Science, Monash University, Clayton, Australia; 257 Università di Torino, Turin, Italy; 258 Bethel University, St. Paul, MN USA; 259 Karamanoğlu Mehmetbey University, Karaman, Turkey; 260 California Institute of Technology, Pasadena, CA USA; 261 United States Naval Academy, Annapolis, MD USA; 262 Ain Shams University, Cairo, Egypt; 263 Bingol University, Bingol, Turkey; 264 Georgian Technical University, Tbilisi, Georgia; 265 Sinop University, Sinop, Turkey; 266 Erciyes University, Kayseri, Turkey; 267 Institute of Modern Physics and Key Laboratory of Nuclear Physics and Ion-beam Application (MOE), Fudan University, Shanghai, China; 268 Texas A &M University at Qatar, Doha, Qatar; 269 Kyungpook National University, Daegu, Korea; 270 another institute or international laboratory covered by a cooperation agreement with CERN, Geneva, Switzerland; 271 Yerevan Physics Institute, Yerevan, Armenia; 272 University of Florida, Gainesville, FL USA; 273 Imperial College, London, UK; 274 Institute of Nuclear Physics of the Uzbekistan Academy of Sciences, Tashkent, Uzbekistan; 275 CERN, 1211 Geneva 23, Switzerland

## Abstract

New sets of parameter tunes for two of the colour reconnection models, quantum chromodynamics-inspired and gluon-move, implemented in the pythia  8 event generator, are obtained based on the default CMS pythia  8 underlying-event tune, CP5. Measurements sensitive to the underlying event performed by the CMS experiment at centre-of-mass energies $\sqrt{s}=7$ and 13$\,\text {Te\hspace{-.08em}V}$, and by the CDF experiment at 1.96$\,\text {Te\hspace{-.08em}V}$ are used to constrain the parameters of colour reconnection models and multiple-parton interactions simultaneously. The new colour reconnection tunes are compared with various measurements at 1.96, 7, 8, and 13$\,\text {Te\hspace{-.08em}V}$ including measurements of the underlying-event, strange-particle multiplicities, jet substructure observables, jet shapes, and colour flow in top quark pair (${{\text {t}} {}{\bar{\text {t}}}}$) events. The new tunes are also used to estimate the uncertainty related to colour reconnection modelling in the top quark mass measurement using the decay products of ${{\text {t}} {}{\bar{\text {t}}}}$ events in the semileptonic channel at 13$\,\text {Te\hspace{-.08em}V}$.

## Introduction

Monte Carlo (MC) event generators, such as pythia  8  [1], are indispensable tools for measurements at the LHC proton–proton (pp) collider. To provide an accurate description of high-energy collisions, both the hard scattering and the so-called underlying event (UE) are computed for each simulated event. In the hard scattering process, two initial partons interact with a large exchange of transverse momentum, $p_{\textrm{T}} > \mathcal {O}(\text {Ge\hspace{-.08em}V} {})$ (we use natural units with $c=1$ throughout the paper). The UE represents additional activity occurring at lower energy scales that accompany the hard scattering. It consists of multiple-parton interactions (MPIs), initial- and final-state radiation (ISR and FSR), and beam-beam remnants (BBR). According to Quantum Chromodynamics (QCD), strong interactions are affected by colour charges that are carried by quarks and gluons. All of the coloured partons produced by these components are finally combined to form colourless hadrons through the hadronisation process.

Particularly relevant for the characterisation of the UE are the MPIs, which consist of additional 2-to-2 parton–parton interactions occurring within the single collision event. With increasing collision energy, the interaction probability for partons with small longitudinal momentum fractions also increases, which enhances MPI contributions.

**Fig. 1:**
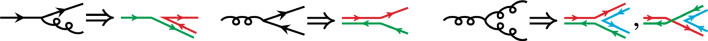
Rules for colour flow for quark-gluon vertices. Figure is taken from Ref. [5]. Quark-gluon vertices are shown in black with Feynman diagrams and colour connection lines are shown with coloured lines

The pythia  8 generator regularises the cross sections of the primary hard scattering processes and MPIs with respect to the perturbative 2-to-2 parton–parton differential cross section through an energy-dependent dampening parameter $p_\textrm{T0}$, which depends on the centre-of-mass energy $\sqrt{s}$. The energy dependence of the $p_\textrm{T0}$ parameter in pythia  8 is described with a power law function of the form

1 $$\begin{aligned} p_\textrm{T0} (\sqrt{s})= p_\textrm{T0} ^\text {ref} \left( \frac{\sqrt{s}}{\sqrt{s_0}}\right) ^\epsilon , \end{aligned}$$

where $p_\textrm{T0} ^{\text {ref}}$ is the value of $p_\textrm{T0}$ at a reference energy $\sqrt{s_0}$, and $\epsilon$ is a tunable parameter that determines the energy dependence. At a given $\sqrt{s}$, the mean number of additional interactions from MPI depends on $p_\textrm{T0}$, the parton distribution functions (PDFs), and the overlap of the matter distributions of the two colliding hadrons [2].

To track the colour information during the development of the parton shower, partons are represented and also connected by colour lines. Quarks and antiquarks are represented by colour lines with arrows pointing in the direction of the colour flow, and gluons are represented by a pair of colour lines with opposite arrows. Rules for colour propagation are shown in Fig. 1. Because each MPI system adds coloured partons to the final state, a dense net of colour lines that overlap with the coloured parton fields of the hard scattering and with each other is created. Parton shower algorithms, in general, use the leading colour (LC) approximation [3, 4] in which each successively emitted parton is colour connected only to its parent emitters in the limit of infinite number of colours. Colour reconnection (CR) models allow colour lines to be formed between partons also from different interactions and thus allow different colour topologies compared with a simple LC approach.

The CR was first included in minimum-bias (MB) simulations (see Sect. 4.1) to reproduce the increase of average transverse momentum $\langle p_{\textrm{T}} \rangle$ of charged particles as a function of the measured multiplicity of the charged particles, $N_{\textrm{ch}}$, and also to describe the $\text{ d }N_{\textrm{ch}}/\text{d }\eta$ distribution [6, 7]. The pseudorapidity is defined as $\eta =-\ln [\tan (\theta /2)]$, where the polar angle $\theta$ is defined with respect to the anticlockwise-beam direction. Introducing correlations between partons, including those also resulting from MPIs, generally changes the number of charged particles in an event and allows a more realistic simulation of $N_{\textrm{ch}}$, and $\langle p_{\textrm{T}} \rangle$ vs $N_{\textrm{ch}}$ distributions than in an event scenario without CR [7].

The CR effects are also important for processes occurring at larger scales in pp collisions. For example, in ${{\text {t}} {}{\bar{\text {t}}}}$ events, the inclusion of CR effects can lead to a significant improvement in the description of UE variables [8]. The effects of CR may become more prominent in precision measurements, such as the top quark mass $m_{{\text {t}}}$. Uncertainties in $m_{{\text {t}}}$ related to CR are usually estimated from comparing the prediction of a given model with and without CR, which might underestimate their effect [9]. A better way to approach the uncertainty estimation would be to consider a variety of CR models and variations of their parameters [10] that probe the effects of the underlying soft physics of pp collisions on the relevant observable.

Various phenomenological models for CR have been developed and are included in pythia  8. In these models, the general idea is to determine the partonic configuration that reproduces the minimal total string length. In the Lund string fragmentation model [11] used in pythia  8, the confining colour field between two partons is approximated by a one-dimensional string stretched between the partons according to the colour flow. The fragmentation of a string with a probability given by the fragmentation function produces a set of hadrons. Thus, the colour flow of an event determines the string configuration and therefore hadronic production.

None of the MPI processes or the CR models are completely determined from first principles, and they all include free parameters. A specified set of such parameters that is adjusted to better fit some aspects of the data is referred to as a “tune”. It is possible to derive a tune that describes the data at a particular $\sqrt{s}$. However, such a model, without energy dependence, will be biased and cannot provide any reliable information about other $\sqrt{s}$. Thus, whenever the collision energy ($\sqrt{s}$) has changed, additional constraints on the models must be applied using the information obtained from the new measurements. This is not a straightforward procedure since no single tune can describe all the data with the same precision. The default CMS pythia  8 tune CUETP8M1 for 7$\,\text {Te\hspace{-.08em}V}$  [12] was derived using the inputs from the 0.9, 1.96 and 7$\,\text {Te\hspace{-.08em}V}$ measurements, and it describes the data at 7$\,\text {Te\hspace{-.08em}V}$ quite well. The default CMS pythia  8 tune CP5, where CP stands for “CMS pythia  8 ” for 13$\,\text {Te\hspace{-.08em}V}$  [13] was derived using the inputs from the 1.96, 7 and 13$\,\text {Te\hspace{-.08em}V}$ measurements. The CUETP8M1 describes data at 7$\,\text {Te\hspace{-.08em}V}$ better than CP5, but the overall performance of CP5 is much better than CUETP8M1 when 13$\,\text {Te\hspace{-.08em}V}$ data are also included.

This paper presents results from two tunes, which make use of the QCD-inspired [14] and the gluon-move [9] CR models. The new CR tunes presented are based on the default CMS pythia  8 tune CP5. Along with the CP5 tune, which is derived from the MPI-based CR model, the performance of the new CR tunes (CP5-CR1 and CP5-CR2 defined below) is studied using several observables. These tunes can be used for the evaluation of the uncertainties due to CR effects, and deepening the understanding of the CR mechanism.

The paper is organised as follows. In Sect. 2, the different colour reconnection models implemented in pythia  8 and used in this study are introduced. In Sect. 3, the tuning strategy is explained in detail and the parameters of the new tunes are presented. Section 4 shows a selection of validation plots related to observables measured at $\sqrt{s}=1.96$, 7, 8, and 13$\,\text {Te\hspace{-.08em}V}$ by various experiments compared with the predictions of the new tunes. In Sect. 5 a study of the uncertainty in the top quark mass $m_{{\text {t}}}$ measurement because of the CR modelling is presented before summarising the results in Sect. 6.

## Colour reconnection models

The MPI-based CR model was the only CR model implemented in pythia  8 until pythia 8.2, which was released with two additional CR models. The models implemented in pythia 8.2, referred to as the “MPI-based”, “QCD-inspired”, and “gluon-move” CR models, are briefly described in the following:

- ***MPI-based model (CP5):*** The simplest model [6, 15] implemented in MC event generators introduces only one tunable parameter. In this model, the partons are classified according to the MPI system to which they belong. Each parton interaction is originally a $2\rightarrow 2$ scattering. For an MPI system with a hardness scale $p_{\textrm{T}}$ of the $2\rightarrow 2$ interaction, a CR probability is defined as:
  2 $$\begin{aligned} P = \frac{p_{\textrm{T}_{\textrm{Rec}}} ^2}{(p_{\textrm{T}_{\textrm{Rec}}} ^2+p_{\textrm{T}} ^2)}, \end{aligned}$$
  with $p_{\textrm{T}_{\textrm{Rec}}} = r p_\textrm{T0}$, where *r* is a tunable parameter and $p_\textrm{T0}$ is the energy-dependent dampening parameter defined in Eq. (1). The parameter $p_\textrm{T0}$ avoids a divergence of the partonic cross section at low $p_{\textrm{T}}$. According to Eq. (2), MPI systems at high $p_{\textrm{T}}$ would tend to escape from the interaction point, without being colour reconnected to the hard scattering system. Colour fields originating from a low-$p_{\textrm{T}}$ MPI system would instead more likely exchange colour. Once the systems to be connected are determined, partons of low-$p_{\textrm{T}}$ systems are added to strings defined by the highest $p_{\textrm{T}}$ system to achieve a minimal total string length.
- ***QCD-inspired model (CP5-CR1):*** The QCD-inspired model [14] implemented in pythia  8 adds the QCD colour rules on top of the minimisation of the string length. The model constructs all pairs of QCD dipoles allowed to be reconnected by QCD colour rules that determine the colour compatibility of two strings. This is done iteratively until none of the allowed reconnection possibilities result in a shortening of the total string length. It uses a simple picture to causally connect the produced strings in spacetime through a string length measure $\lambda$ to determine favoured reconnections. The default parametrisation for $\lambda$ is
  3 $$\begin{aligned} \lambda = \ln \left( 1+\sqrt{2}\frac{E_1}{m_0}\right) + \ln \left( 1+\sqrt{2}\frac{E_2}{m_0}\right) , \end{aligned}$$
  where $E_1$ and $E_2$ represent the energies of the coloured partons in the rest frame of the QCD dipole, and $m_0$ is a constant with the dimension of energy [14]. In addition, the QCD-inspired model allows us to create junction structures. A junction is a topological structure and is formed when three colour lines meet at a single point. The presence of junctions reduces the number of colour lines that need to be connected to the beam remnant, which in turn can affect the number of particles produced in a collision. Since the QCD-inspired CR model allows for different color topologies beyond LC, it can successfully describe the baryon production measured at the CMS experiment [14, 16], which is not the case for previously available pythia  8 tunes.
- ***Gluon-move model (CP5-CR2):*** In this scheme [9], final-state gluons are identified along with all the colour-connected pairs of partons. Then an iterative process starts. The difference between string lengths when a final-state gluon belonging to two connected partons is moved to another connected two-parton system is calculated. The gluon is moved to the string for which the move gives the largest reduction in total string length. This procedure can be repeated for all or a fraction of the gluons in the final state, which is controlled by the pythia  8 parameter ColourReconnection:fracGluon. In this scheme, quarks would not be reconnected, i.e. they would remain in the same position without any colour exchange. To improve this picture, the flip mechanism of the gluon-move model can be included. The flip mechanism basically allows reconnection of two different string systems, i.e. a quark can connect to a different antiquark. Junctions (Y-shaped three-quark configurations) are allowed to take part in the flip step as well, but no considerable differences are expected due to the limitation of the junction formation in this model. The flip mechanism has not been extensively studied and its effect on diffractive events is not known. For this reason the flip mechanism is switched off in pythia  8 and not used in this paper. The main free parameters of the gluon-move model account for the lower limit of the string length allowed for colour reconnection, the fraction of gluons allowed to move, and the lower limit of the allowed reduction of the string lengths.

In addition to these models, the effects of early resonance decay (ERD) [9] in top quark decays are also studied. With this option, top quark decay products are allowed to participate directly in CR. Normally the ERD option is switched off in pythia  8 but in Sect. 4.5 we investigate the ERD effects.

Usually, MPI and CR effects are investigated and constrained using fits to measurements sensitive to the UE in hadron collisions. The UE measurements have been performed at various collision energies by ATLAS, CMS, and CDF Collaborations [17–21]. The measurements are typically performed by studying the multiplicity and the scalar $p_{\textrm{T}}$ sum of the charged particles ($p_{\textrm{T}} ^{\text {sum}}$), measured as a function of the $p_{\textrm{T}}$ of the leading charged particle in the event, $p_{\textrm{T}} ^{\text {max}}$.

**Fig. 2:**
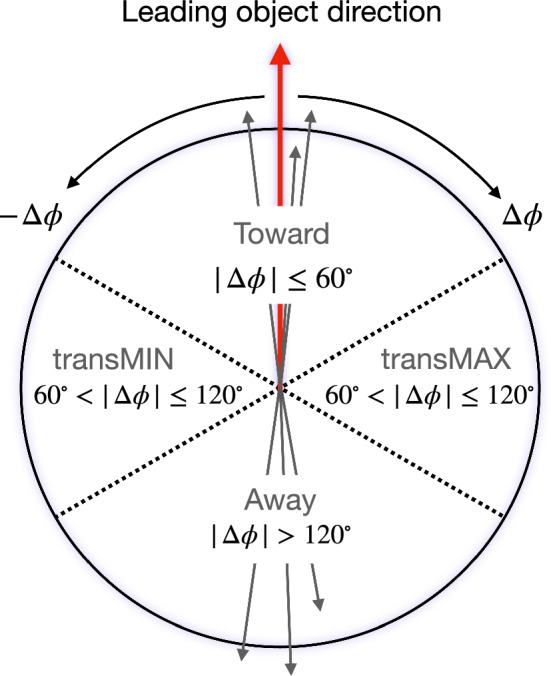
The schematic description of the result of a typical hadron-hadron collision. The “toward” region contains the “toward-side” jet, whereas the “away” region may contain an “away-side” jet

Different regions of the plane transverse to the direction of the beams are defined by the direction of the leading charged particle. A sketch of the different regions is shown in Fig. 2. A “toward” region includes mainly the products of the hard scattering, whereas the “away” region includes the recoiling objects belonging to the hard scattering. The two “transverse” regions contain the products of MPIs and are affected by contributions from ISR and FSR.

In Refs. [17, 18, 21], the transverse region is further subdivided into “transMIN” and “transMAX”, defined to be the regions with the minimum and maximum number of particles between the two transverse regions. This is done to disentangle contributions from MPI, ISR, and FSR. For events with large ISR or FSR, the transMAX region contains at least one “transverse-side” jet, whereas both the transMAX and transMIN regions contain particles from the MPI and BBR. Thus, the transMIN region is sensitive to MPI and BBR, whereas the difference between transMAX and transMIN (referred to as the transDIFF region) is sensitive to ISR and FSR.

**Fig. 3:**
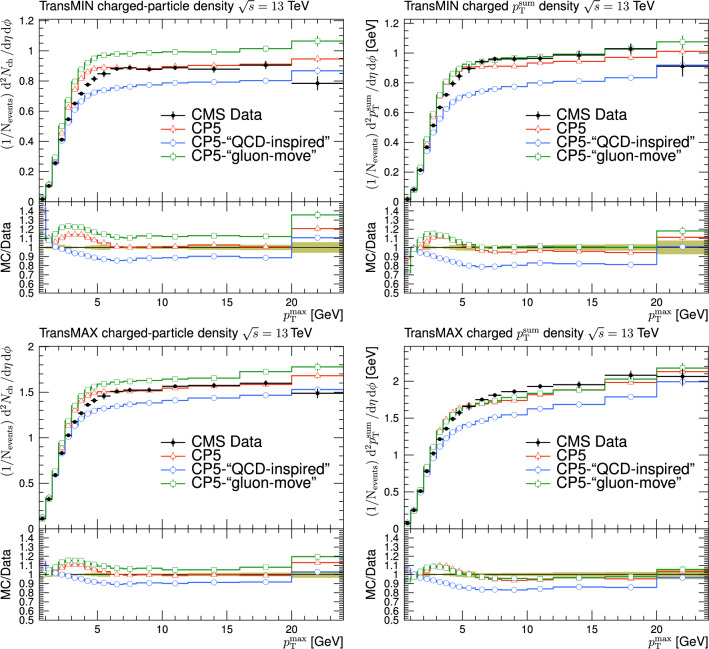
The charged-particle (left) and $p_{\textrm{T}} ^{\text {sum}}$ densities (right) in the transMIN (upper) and transMAX (lower) regions as functions of the $p_{\textrm{T}}$ of the leading charged particle, $p_{\textrm{T}} ^{\text {max}}$, measured by the CMS experiment at $\sqrt{s}=13\,\text {Te\hspace{-.08em}V}$ [17]. The predictions of the tunes CP5, CP5-“QCD-inspired”, and CP5-“gluon-move” using their default parameter settings in Refs. [9, 14], are compared with data. The coloured band and error bars on the data points represent the total experimental uncertainty in the data where the model uncertainty is also included. The comparisons show that the models do not describe the data and need to be retuned

The CMS Collaboration showed that a consistent description of the $N_{\textrm{ch}}$ and the $p_{\textrm{T}} ^{\text {sum}}$ distributions is not possible using only the pythia  8 hadronisation model without taking into account the CR effects [12]. In general, the largest difference between the predictions from tunes and the data is observed in the soft region ($p_{\textrm{T}} \sim 2\text {--}5\,\text {Ge\hspace{-.08em}V}$), where CR effects are expected to be more relevant.

**Fig. 4:**
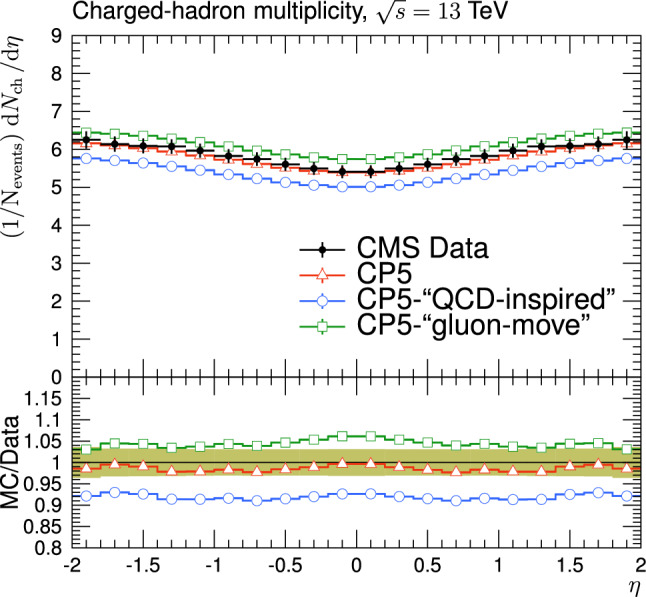
The pseudorapidity of charged hadrons, $\text{ d }N_{\textrm{ch}}/\text{d }\eta$, measured in $|\eta |<2$ by the CMS experiment at $\sqrt{s}=13\,\text {Te\hspace{-.08em}V}$ [23]. The predictions of the tunes CP5, CP5-“QCD-inspired”, and CP5-“gluon-move” using their default parameter settings in Refs. [9, 14], are compared with data. The coloured band and error bars on the data points represent the total experimental uncertainty in the data where model uncertainty is also included. The comparisons show that the models need to be retuned in order to have a better agreement with the data

The new CR models, QCD-inspired and gluon-move, were implemented in pythia 8.226 after tuning the model parameters to the existing data at $\sqrt{s}=7\,\text {Te\hspace{-.08em}V}$ and at lower centre-of-mass energies [9, 14]. The models were tuned to different data sets starting from different baseline tune settings. The model predictions, with their default parameter settings in pythia 8.226 and CP5, are given in Fig. 3 for $N_{\textrm{ch}}$ and $p_{\textrm{T}} ^{\text {sum}}$ densities measured by the CMS experiment at 13$\,\text {Te\hspace{-.08em}V}$  [17] in the transMIN and transMAX regions, and in Fig. 4 for the $\text{ d }N_{\textrm{ch}}/\text{d }\eta$ distribution measured by CMS at 13$\,\text {Te\hspace{-.08em}V}$  [22]. In these figures, the data points, shown in black, are well described by CP5. The predictions for CP5-“QCD-inspired” and CP5-“gluon-move” were obtained by replacing the MPI-based CR model in CP5 with the QCD-inspired and gluon-move CR model, respectively. As mentioned earlier, these models were tuned to data at 7$\,\text {Te\hspace{-.08em}V}$ and at lower centre-of-mass energies. The comparisons show that the models must be retuned to describe the underlying soft physics of pp collisions at 13$\,\text {Te\hspace{-.08em}V}$.

## The new CMS colour reconnection tunes

A new set of event tunes, based on UE data from the CMS and CDF experiments, are derived using the QCD-inspired and the gluon-move CR models, as implemented in the pythia 8.226 event generator. Having tunes for different CR models allows a consistent way of evaluating systematic uncertainties because of colour reconnection effects in specific measurements. The Rivet  2.4.0 [24] routines used as inputs to the fits, as well as the centre-of-mass energy values and the names of the Rivet distributions, the *x*-axis ranges (fit ranges), and the relative importance (*R*) of the distributions are displayed in Table 1 for the tunes CP5-CR1 and CP5-CR2. The CP5 tune is used as a baseline for the CR tuning since it is the default pythia  8 tune for most of the new CMS analyses using data at $\sqrt{s}=13\,\text {Te\hspace{-.08em}V}$ published since 2017, and it has explicitly been tested against a large number of different final states (MB, QCD, top quark, and vector boson + jets) and observables [13].

The parameters and their ranges in the fits are shown in Table 2. The minimum and maximum values of the parameters are first taken from pythia  8, then the ranges of the values are further limited using the Professor  1.4.0 software [25]. The ranges are chosen such that the sampled MC space does not destroy the definition of a particular observable in the fits.

**Table 1:** List of input Rivet routines, centre-of-mass energy values, $\eta$ ranges, names of distributions, fit ranges, and relative importance of the distributions used in the fits to derive the tunes CP5-CR1 and CP5-CR2

Rivet routine	$\sqrt{s}$ [$\text {Te\hspace{-.08em}V}$ ]	$|\eta |$	Distribution	CP5-CR1 Fit range [$\text {Ge\hspace{-.08em}V}$ ]	*R*	CP5-CR2 Fit range [$\text {Ge\hspace{-.08em}V}$ ]	*R*
CMS_2015_I1384119 [23]	13	${<}$2.0	$N_{\textrm{ch}}$ versus $\eta$		1		1
CMS_2015_PAS_FSQ_15_007 [17]	13	${<}$2.0	TransMIN $p_{\textrm{T}} ^{\text {sum}}$	2–28	1	3–36	0.5
			TransMAX $p_{\textrm{T}} ^{\text {sum}}$	2–28	1	3–36	0.5
			TransMIN $N_{\textrm{ch}}$	2–28	1	3–36	0.1
			TransMAX $N_{\textrm{ch}}$	2–28	1	3–36	0.1
CMS_2012_PAS_FSQ_12_020 [18]	7	${<}$0.8	TransMAX $N_{\textrm{ch}}$	3–20	1	3–20	0.1
			TransMIN $N_{\textrm{ch}}$	3–20	1	3–20	0.1
			TransMAX $p_{\textrm{T}} ^{\text {sum}}$	3–20	1	3–20	0.1
			TransMIN $p_{\textrm{T}} ^{\text {sum}}$	3–20	1	3–20	0.1
CDF_2015_I1388868 [21]	2	${<}$0.8	TransMIN $N_{\textrm{ch}}$	2–15	1	2–15	0.1
			TransMAX $N_{\textrm{ch}}$	2–15	1	2–15	0.1
			TransMIN $p_{\textrm{T}} ^{\text {sum}}$	2–15	1	2–15	0.1
			TransMAX $p_{\textrm{T}} ^{\text {sum}}$	2–15	1	2–15	0.1

Tune CP5 uses the next-to-next-to-leading order (NNLO) NNPDF31_nnlo_as_0118 [26] PDF set, the strong coupling parameter $\alpha _\textrm{S}$ value of 0.118 for ISR, FSR, and MPI, and the MPI-based CR model. It also uses a double-Gaussian functional form with two tunable parameters, coreRadius and coreFraction, to model the overlap of the matter distribution of the two colliding protons [6]. The tune parameters are documented in Ref. [13] and displayed in Table 3. Also in Figs. 11 and 12 in Ref. [13], predictions of the CP5 tune are compared with event shape observables measured at LEP. The results show that a value of $\alpha _\textrm{S} ^\textrm{FSR}(m_\text {Z})$ $\sim$0.120 better describes the data compared with higher values of $\alpha _\textrm{S} ^\textrm{FSR}(m_\text {Z})$ which generally overestimates the number of final-state partons. As concluded in the Ref. [13], LEP event shape observables are well described by MadGraph 5_amc@nlo  + pythia  8 with CP5.

The new tunes are obtained by constraining simultaneously the parameters controlling the contributions of the MPI and of each of the CR models. The strategy followed to obtain the CP5-CR1 and CP5-CR2 tunes is similar to that used for the CP5 tune, i.e. the same observables sensitive to MPI are considered to constrain the parameters. These are the $N_{\textrm{ch}}$ and average $p_{\textrm{T}} ^{\text {sum}}$ as functions of the leading charged particle transverse momentum $p_{\textrm{T}} ^{\text {max}}$, measured in the transMIN and transMAX regions by the CMS experiment at $\sqrt{s}=13\,\text {Te\hspace{-.08em}V}$ [17] and 7$\,\text {Te\hspace{-.08em}V}$  [19] and by the CDF experiment at 1.96$\,\text {Te\hspace{-.08em}V}$  [21]. The $N_{\textrm{ch}}$ as a function of $\eta$, measured by CMS at $\sqrt{s}=13\,\text {Te\hspace{-.08em}V}$ [22] is also used in the fit. In Ref. [17], the transMIN and transMAX regions are defined with respect to both the leading charged particles and the leading charged-particle jets as reference objects. The uncertainty in measurements using leading charged particles as reference objects is lower than the uncertainty in measurements using leading charged-particle jets as reference objects. This is one of the reasons why we choose to use leading charged particle observables instead of leading charged-particle jet observables in the fits. Another reason is that we want to use the same observables that were used to derive CP5, and CP5 was derived using leading charged particle observables in the fits. As a cross-check, we also derived another version of the CP5-CR1 tune using leading charged-particle jet observables, such as $N_{\textrm{ch}}$ and average $p_{\textrm{T}} ^{\text {sum}}$, as functions of the transverse momentum of the leading charged-particle jets in the fits. The results showed that the use of leading charged-particle jet observables in the fits makes a very small difference, which is negligible when tune uncertainties are taken into account. As for CP5, the region with $p_{\textrm{T}} ^{\text {max}}$ between 0.5 and 2.0 or 3.0$\,\text {Ge\hspace{-.08em}V}$ is excluded depending on the distribution from the fit, since this region is affected by diffractive processes whose free parameters are not considered in the tuning procedure.

The MPI-related parameters that are kept free in both the CP5-CR1 and CP5-CR2 tunes are:

- MultipartonInteractions:pT0Ref, the parameter $p_\textrm{T0} ^{\text {ref}}$ included in the regularisation of the partonic QCD cross section as described in Eq. (1). It sets the lower cutoff scale for MPIs;
- MultipartonInteractions:ecmPow, the exponent $\epsilon$ of the $\sqrt{s}$ dependence as shown in Eq. (1);
- MultipartonInteractions:coreRadius, the width of the core when a double-Gaussian matter profile is assumed for the overlap distribution between the two colliding protons [6]. A double-Gaussian form identifies an inner, dense part, which is called core, and an outer, less dense part;
- MultipartonInteractions:coreFraction, the fraction of quarks and gluons contained in the core when a double-Gaussian matter profile is assumed.

**Table 2:** The MPI and CR parameter ranges used in the tuning procedure

pythia 8 parameter	Min–Max
MPI parameters	
MultipartonInteractions:pT0Ref	1.0–3.0
MultipartonInteractions:ecmPow	0.0–0.3
MultipartonInteractions:coreRadius	0.2–0.8
MultipartonInteractions:coreFraction	0.2–0.8
QCD-inspired model	
ColourReconnection:m0	0.1–4.0
ColourReconnection:junctionCorrection	0.01–10
ColourReconnection:timeDilationPar	0–60
Gluon-move model	
ColourReconnection:m2lambda	0.2–8.0
ColourReconnection:fracGluon	0.8–1.0

The tunable CR parameters in CP5-CR1 that are considered in the fit are:

- ColourReconnection:m0, the variable that determines whether a possible reconnection is actually favoured in the $\lambda$ measure in Eq. (3);
- ColourReconnection:junctionCorrection, the multiplicative correction for junction formation, applied to the m0 parameter;
- ColourReconnection:timeDilationPar, the parameter controlling the time dilation that forbids colour reconnection between strings that are not in causal contact.

More details on these parameters are reported in Ref. [1]. For the CP5-CR1 tune, the parameters related to the hadronisation, StringZ:aLund, StringZ:bLund, StringFlav:probQQtoQ, and StringFlav:probStoUD, proposed in Ref. [14], are also used as fixed inputs to the tune. The first two of these parameters govern the longitudinal fragmentation function used in the Lund string model in pythia  8, whereas the latter two are the probability of diquark over quark fragmentation, and the ratio of strange to light quark production, respectively.

For the optimisation of CP5-CR2, the following parameters are considered:

- ColourReconnection:m2lambda, an approximate hadronic mass-square scale and the parameter used in the calculation of $\lambda$;
- ColourReconnection:fracGluon, the probability that a given gluon will be moved. It thus gives the average fraction of gluons being considered.

**Table 3:** The parameters obtained in the fits of the CP5-CR1 and CP5-CR2 tunes, compared with that of the CP5 tune. The upper part of the table displays the fixed input parameters of the tune, whereas the lower part shows the fitted tune parameters. The number of degrees of freedom ($N_\textrm{dof}$) and the goodness of fit divided by $N_\textrm{dof}$ are also shown

pythia 8 parameter	CP5 [13]	CP5-CR1	CP5-CR2
PDF set	NNPDF3.1 NNLO	NNPDF3.1 NNLO	NNPDF3.1 NNLO
$\alpha _\textrm{S} (m_\text {Z})$	0.118	0.118	0.118
SpaceShower:rapidityOrder	On	On	On
MultipartonInteractions:ecmRef [$\text {Ge\hspace{-.08em}V}$ ]	7000	7000	7000
$\alpha _\textrm{S} ^\textrm{ISR}(m_\text {Z})$ value/order	0.118/NLO	0.118/NLO	0.118/NLO
$\alpha _\textrm{S} ^\textrm{FSR}(m_\text {Z})$ value/order	0.118/NLO	0.118/NLO	0.118/NLO
$\alpha _\textrm{S} ^\textrm{MPI}(m_\text {Z})$ value/order	0.118/NLO	0.118/NLO	0.118/NLO
$\alpha _\textrm{S} ^\textrm{ME}(m_\text {Z})$ value/order	0.118/NLO	0.118/NLO	0.118/NLO
StringZ:aLund	–	0.38	–
StringZ:bLund	–	0.64	–
StringFlav:probQQtoQ	–	0.078	–
StringFlav:probStoUD	–	0.2	–
SigmaTotal:zeroAXB	Off	Off	Off
BeamRemnants:remnantMode	–	1	–
ColourReconnection:mode	–	1	2
MultipartonInteractions:pT0Ref [$\text {Ge\hspace{-.08em}V}$ ]	1.410	1.375	1.454
MultipartonInteractions:ecmPow	0.033	0.033	0.054
MultipartonInteractions:coreRadius	0.763	0.605	0.649
MultipartonInteractions:coreFraction	0.630	0.445	0.489
ColourReconnection:range	5.176	–	–
ColourReconnection:junctionCorrection	–	0.238	–
ColourReconnection:timeDilationPar	–	8.580	–
ColourReconnection:m0	–	1.721	–
ColourReconnection:m2lambda	–	–	4.917
ColourReconnection:fracGluon	–	–	0.993
$N_\textrm{dof}$	183	157	158
$\chi ^{*2} / N_\textrm{dof}$	1.04	2.37	0.89

**Fig. 5:**
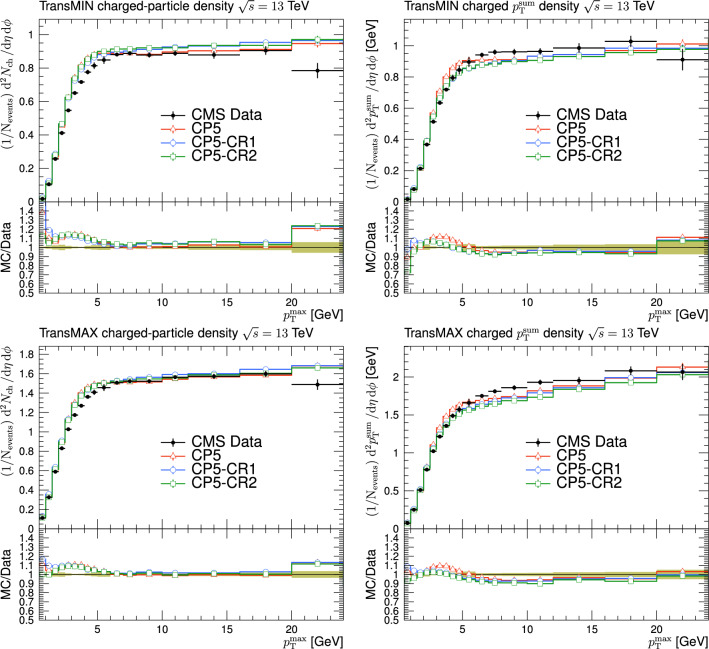
The charged-particle (left) and $p_{\textrm{T}} ^{\text {sum}}$ (right) densities in the transMIN (upper) and transMAX (lower) regions, as functions of the $p_{\textrm{T}}$ of the leading charged particle, $p_{\textrm{T}} ^{\text {max}}$, measured by the CMS experiment at $\sqrt{s}=13\,\text {Te\hspace{-.08em}V}$ [17]. The predictions of the CP5 and CP5-CR tunes are compared with data. The coloured band and error bars on the data points represent the total experimental uncertainty in the data

The remaining parameters of pythia  8 are kept the same as in the CP5 tune.

The fits are performed using the Professor  1.4.0 software, which takes random values for each parameter in the defined multidimensional parameter space, and Rivet, which provides the data points and uncertainties, and produces the individual generator predictions for the considered observables. About 200 different choices of parameters are considered to build a random grid in the parameter space. For each choice of parameters, one million pp inelastic scattering events, including contributions from single-diffractive dissociation (SD), double-diffractive dissociation (DD), central diffraction (CD), and nondiffractive (ND) processes, are generated. The bin-by-bin envelopes of the different MC predictions are checked. After building the grid in the parameter space, Professor performs an interpolation of the bin values for the observables in the parameter space using a third-order polynomial function. We verified that the degree of the polynomial used for the interpolation does not affect the tune results significantly. The function $f^{b}(\textbf{p})$ models the MC response of each bin *b* of the observable *O* as a function of the parameter vector $\textbf{p}$. The final step is the minimisation of the $\chi ^{*2}$ function given by:

4 $$\begin{aligned} \chi ^{*2}(\textbf{p})=\sum _{O}\sum _{{b}\in O}\frac{(f^{b}(\textbf{p})-\mathcal {R}_{b})^2}{\varDelta _{b}^2}, \end{aligned}$$

where $\mathcal {R}_{b}$ is the data value for each bin *b*, and $\varDelta _{b}^2$ expresses the total bin uncertainty of the data.

The $\chi ^{*2}$ is not a true $\chi ^2$ function as explained in the following. Treating equally all distributions that are used as inputs to the fit for the CP5-CR2 tune results in a tune that describes the data poorly; in particular, it underestimates the $\text{ d }N_{\textrm{ch}}/\text{d }\eta$ distribution measured in data at $\sqrt{s}=13\,\text {Te\hspace{-.08em}V}$ by about 30%. This is because the $\chi ^2$ definition treats all bins equally and the importance of $\text{ d }N_{\textrm{ch}}/\text{d }\eta$ may be lost because of its relatively low precision with respect to other observables. The $\text{ d }N_{\textrm{ch}}/\text{d }\eta$ distribution is one of the key observables that is sensitive to a number of processes and, therefore, increasing the importance of this observable in the fit is reasonable.

In Professor, this is done by using weights with a nonstandard $\chi ^2$ definition. To keep the standard properties of a $\chi ^2$ fit, we increase the total uncertainties of the other distributions. The total uncertainty in each bin is scaled up by $1/\sqrt{R}$ with *R* (relative importance) values displayed in Table 1. Therefore, the total uncertainty of each bin of $p_{\textrm{T}} ^{\text {sum}}$ in the transMIN and transMAX regions at $\sqrt{s}=13\,\text {Te\hspace{-.08em}V}$ is scaled up by $\sqrt{2}$ and that of all other distributions by $\sqrt{10}$. These scale factors ensure that the distributions are well described after the tuning. For the CP5-CR1 model, a good description of the input observables is obtained without scaling, meaning that all distributions are considered equally important.

The experimental uncertainties used in the fit, in general, have bin-to-bin correlations. However, some of the bins of the UE distributions used in the fit, e.g. $p_{\textrm{T}} ^{\text {max}} > 10\,\text {Ge\hspace{-.08em}V}$, are dominated by statistical uncertainties, which are uncorrelated between bins. In the minimisation procedure, because the correlations between bins are not available for the input measurements, the experimental uncertainties are assumed to be uncorrelated between data points.

The parameters obtained from the CP5-CR1 and CP5-CR2 fits, as well as the value of the goodness of the fit are shown in Table 3. Uncertainties in the parameters of these tunes are discussed in Appendix B. In Ref. [13], the number of degrees of freedom ($N_\textrm{dof}$), defined as the sum of the number of bins of fit observables minus the number of fit parameters, for the tune CP5 is given as 63. However, this value of $N_\textrm{dof}$ corresponds to the case when only 13$\,\text {Te\hspace{-.08em}V}$ distributions are used. The value of $N_\textrm{dof}$ for CP5 consistent with our calculation in this paper is 183. The tune CP5 was derived using two additional distributions in the fits; $\text{ d }N_{\textrm{ch}}/\text{d }\eta$ at 13$\,\text {Te\hspace{-.08em}V}$ with NSD-enhanced selection and SD-enhanced selection. Since these two observables depend on modelling of single diffraction dissociation, which is not well understood, they are not included in the fits for CP5-CR tunes. Therefore, the $N_\textrm{dof}$ values for CP5-CR tunes are lower than the $N_\textrm{dof}$ of CP5. The slight difference in the $N_\textrm{dof}$ values between the CP5-CR1 and CP5-CR2 tunes is due to the difference in the number of fit parameters used in each tune, which are 7 and 6 respectively. Although the fit ranges for the CP5-CR tunes differ slightly, as shown in Table 1, the sum of number of bins of fit observables is the same for both tunes.

**Fig. 6:**
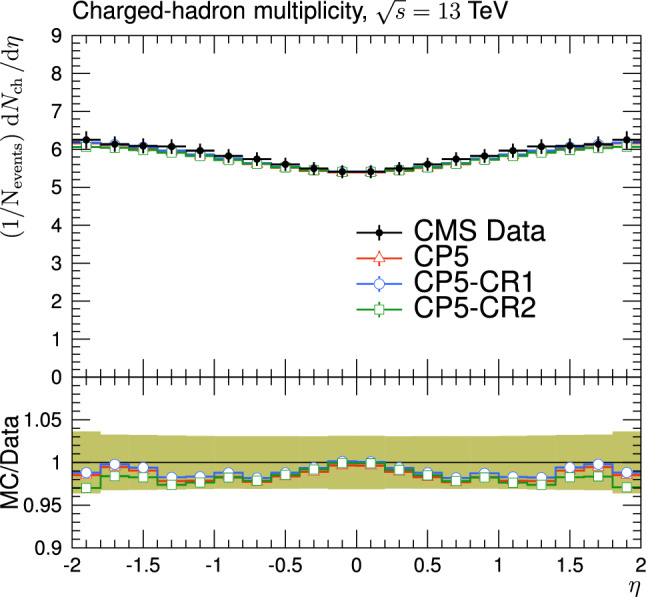
The pseudorapidity of charged hadrons, $\text{ d }N_{\textrm{ch}}/\text{d }\eta$, measured by the CMS experiment at $\sqrt{s}=13\,\text {Te\hspace{-.08em}V}$ [22]. The predictions of the CP5 and CP5-CR tunes are compared with data. The coloured band and error bars on the data points represent the total experimental uncertainty in the data

**Fig. 7:**
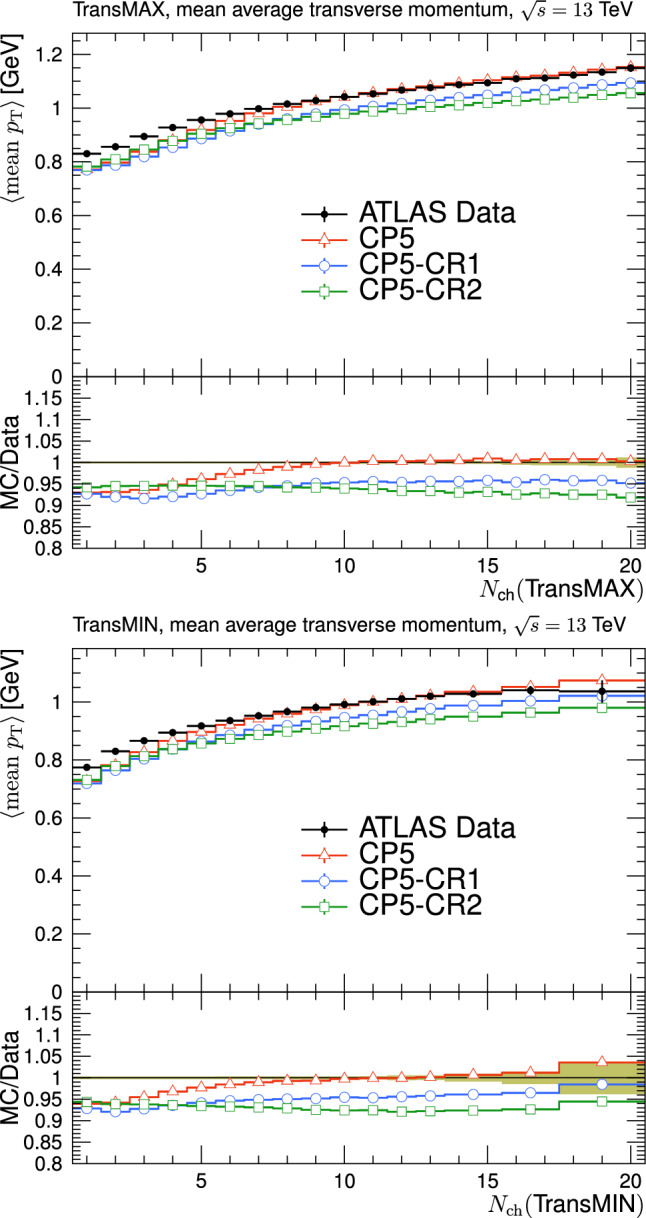
The mean charged-particle average transverse momentum as functions of charged-particle multiplicity in the transMAX (upper) and transMIN (lower) regions, measured by the ATLAS experiment at $\sqrt{s}=13\,\text {Te\hspace{-.08em}V}$ [18]. The predictions of the CP5 and CP5-CR tunes are compared with data. The coloured band and error bars on the data points represent the total experimental uncertainty in the data

A preliminary version of the CP5-CR2 tune was derived including several jet substructure observables [27–29] in the fits. This tune, called CP5-CR2-j, has been used in the MC production in the CMS experiment. The CP5-CR2 and CP5-CR2-j tunes have very similar predictions in all final states discussed in this paper, because the tunes differ slightly only in the following parameters, where the listed values are for CP5-CR2-j:

- MultipartonInteractions:ecmPow = 0.056,
- MultipartonInteractions:coreRadius = 0.653,
- MultipartonInteractions:coreFraction = 0.439,
- ColourReconnection:m2lambda = 4.395,
- MultipartonInteractions:fracGluon = 0.990.

The CP1 and CP2 are the two tunes in the CPX (X = 1–5) tune family [13] that use an LO PDF set [26]. We also derive CR tunes based on the CP1 and CP2 settings to study the effect of using a leading order (LO) PDF set with alternative CR models, although they are not used in precision measurements. We find that the predictions of the CR tunes based on CP1 and CP2 for the MB and UE observables are similar to the predictions of CR tunes based on CP5. However, CP1-CR1 (i.e. CP1 with the QCD-inspired colour reconnection model) has a different trend in particle multiplicity distributions compared with the predictions of other tunes discussed in this study. This different trend of CP1-CR1 cannot be attributed to the use of LO PDF set, because both CP1 and CP2 use the same LO PDF set and we do not see a different trend with CP2-CR1. The different trend observed with CP1-CR1 in the particle multiplicity distributions may become a collective effect rather than a single parameter effect, and could be an input for further tuning and development of the QCD-inspired model. Therefore, in Appendix A of this paper, we present the tune settings of the CR tunes based on CP1 and CP2, along with their predictions in the particle multiplicity distributions.

## Performance of the tunes

In Figs. 5, 6, 7, 8, 9, 10, 11, 12, 13, 14, 15, 16, 17, 18 we show the observables measured at centre-of-mass energies of 1.96, 7, 8, and 13$\,\text {Te\hspace{-.08em}V}$. The data points are shown in black, and are compared with simulations obtained from the pythia  8 event generator with the tunes CP5 (red), CP5-CR1 (blue), and CP5-CR2 (green). For simplicity, the tunes CP5-CR1 and CP5-CR2 will be referred to as CP5-CR when convenient. The lower panels show the ratios between each MC prediction and the data.

**Fig. 8:**
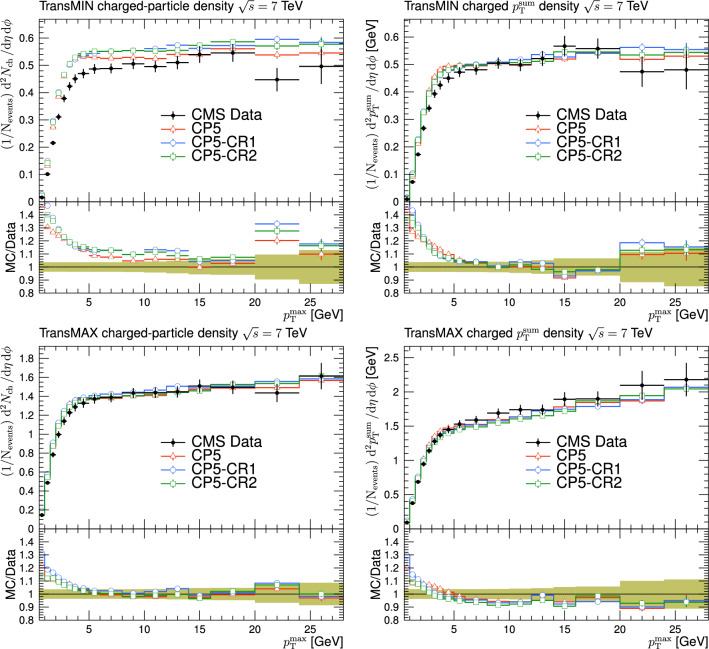
The charged-particle (left) and $p_{\textrm{T}} ^{\text {sum}}$ (right) densities in the transMIN (upper) and transMAX (lower) regions, as functions of the $p_{\textrm{T}}$ of the leading charged particle, $p_{\textrm{T}} ^{\text {max}}$, measured by the CMS experiment at $\sqrt{s}=7\,\text {Te\hspace{-.08em}V}$ [19]. The predictions of the CP5 and CP5-CR tunes are compared with data. The coloured band and error bars on the data points represent the total experimental uncertainty in the data

**Fig. 9:**
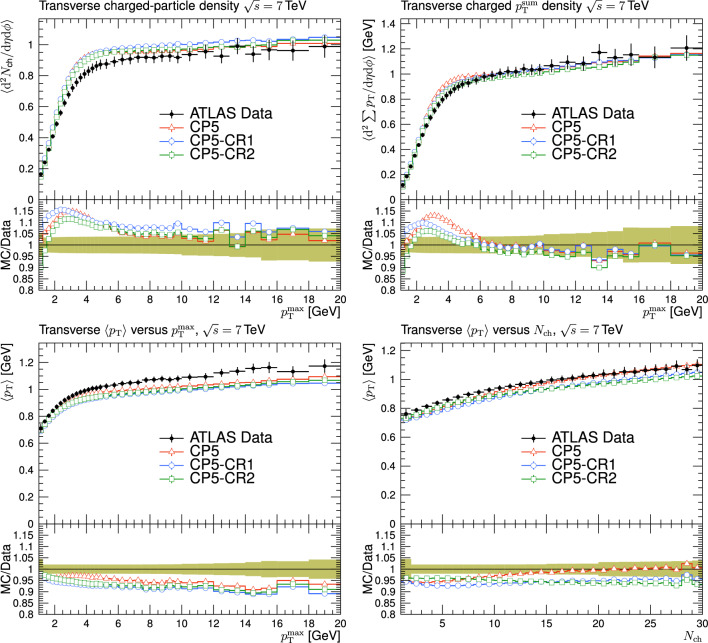
The charged-particle (upper left) and $p_{\textrm{T}} ^{\text {sum}}$ densities (upper right) in the transverse region, as functions of the $p_{\textrm{T}}$ of the leading charged particle, and average transverse momentum in the transverse region as functions of the leading charged particle $p_{\textrm{T}}$ (lower left) and of the charged particle multiplicity (lower right), measured by the ATLAS experiment at $\sqrt{s}=7\,\text {Te\hspace{-.08em}V}$ [20]. The predictions of the CP5 and CP5-CR tunes are compared with data. The coloured band and error bars on the data points represent the total experimental uncertainty in the data

**Fig. 10:**
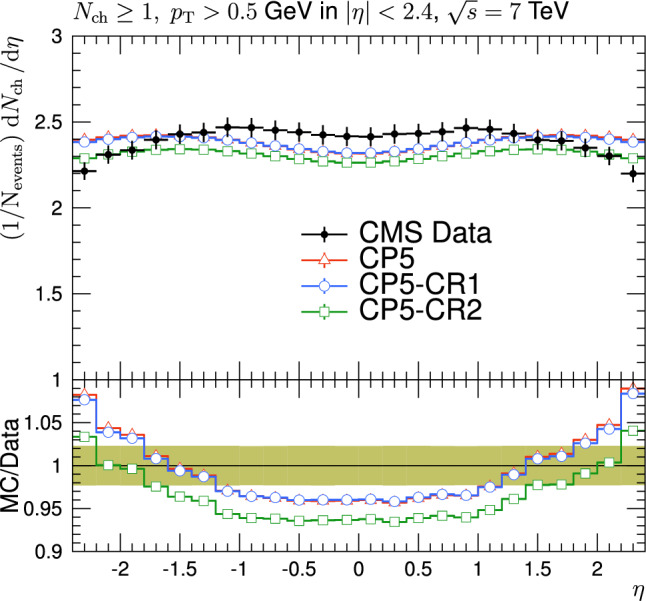
The pseudorapidity of charged particles, $\text{ d }N_{\textrm{ch}}/\text{d }\eta$, with at least one charged particle in $|\eta |<2.4$, measured by the CMS experiment at $\sqrt{s}=7\,\text {Te\hspace{-.08em}V}$ [31]. The predictions of the CP5 and CP5-CR tunes are compared with data. The coloured band and error bars on the data points represent the total experimental uncertainty in the data

**Fig. 11:**
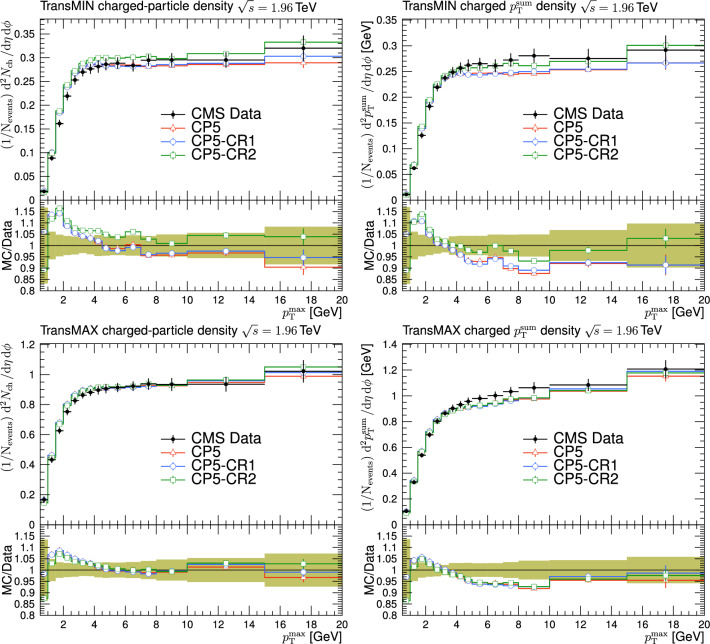
The charged-particle (left) and $p_{\textrm{T}} ^{\text {sum}}$ densities (right) in the transMIN (upper) and transMAX (lower) regions, as functions of the $p_{\textrm{T}}$ of the leading charged particle, $p_{\textrm{T}} ^{\text {max}}$, measured by the CDF experiment at $\sqrt{s}=1.96\,\text {Te\hspace{-.08em}V}$ [21]. The predictions of the CP5 and CP5-CR tunes are compared with data. The coloured band and error bars on the data points represent the total experimental uncertainty in the data

**Fig. 12:**
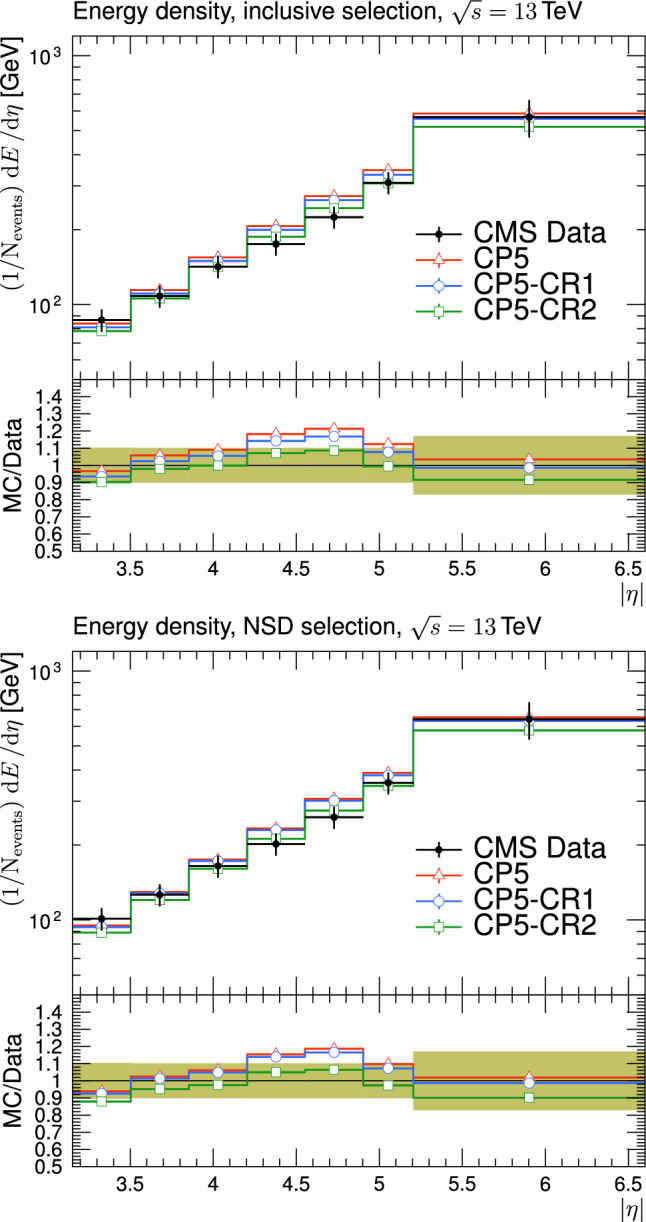
The energy density as a function of pseudorapidity, in two different selections, in MB events (upper) and in events with a presence of a hard dijet system (lower), measured by the CMS experiment at $\sqrt{s}=13\,\text {Te\hspace{-.08em}V}$ [34]. The predictions of the CP5 and CP5-CR tunes are compared with data. The coloured band and error bars on the data points represent the total experimental uncertainty in the data

**Fig. 13:**
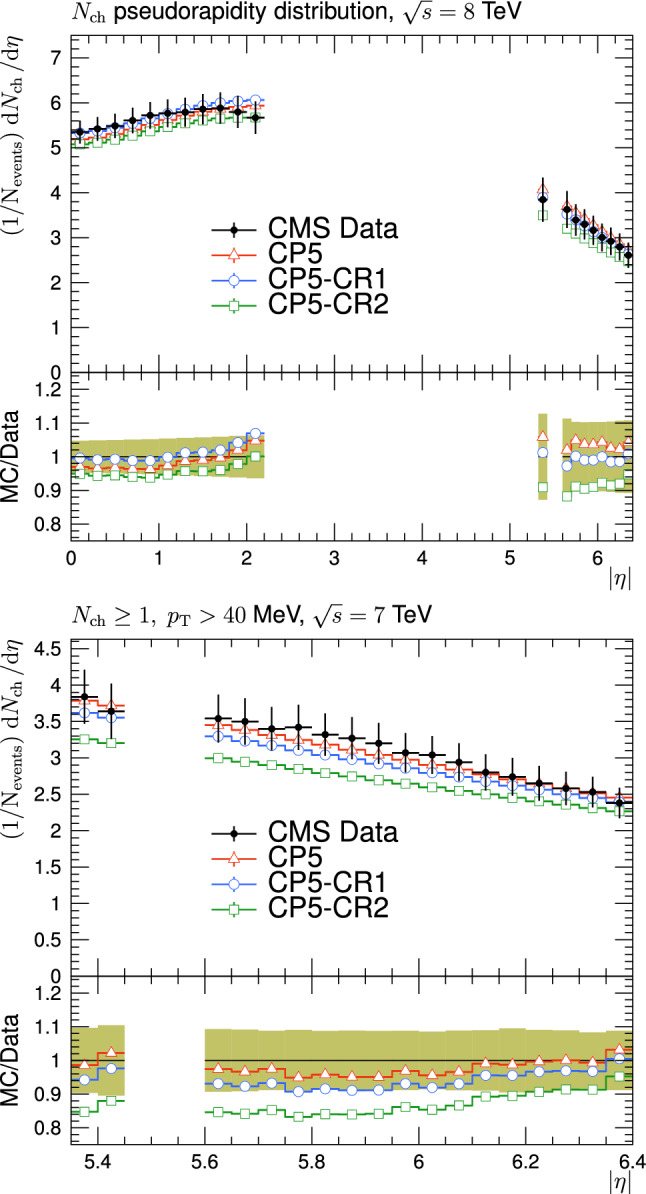
The pseudorapidity of charged particles, $\text{ d }N_{\textrm{ch}}/\text{d }\eta$, measured by the CMS and TOTEM collaborations at $\sqrt{s}=8\,\text {Te\hspace{-.08em}V}$ [32] (upper) and measured by the TOTEM collaboration at $\sqrt{s}=7\,\text {Te\hspace{-.08em}V}$ [33] (lower). The predictions of the CP5 and CP5-CR tunes are compared with data. The coloured band and error bars on the data points represent the total experimental uncertainty in the data. For the CMS-TOTEM measurement, at least one charged particle with $p_{\textrm{T}} >0$ is required in $5.3<\eta <6.5$ or $-6.5<\eta <-5.3$. For the TOTEM measurement, at least one charged particle with $p_{\textrm{T}} >40\,\text {Me\hspace{-.08em}V}$ is required in $5.3<|\eta |<6.4$

**Fig. 14:**
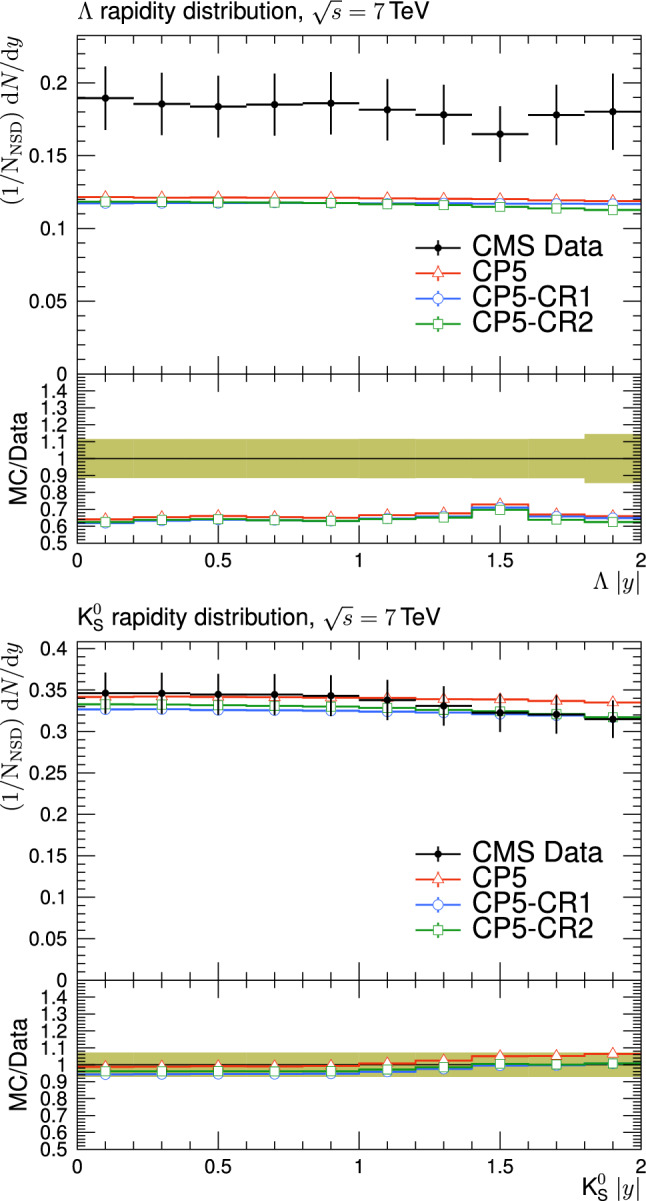
The strange particle production, $\varLambda$ baryons (upper) and $\text {K}_{\textrm{S}}^{0}$ mesons (lower), as a function of rapidity, measured by the CMS experiment at $\sqrt{s}=7\,\text {Te\hspace{-.08em}V}$ [16]. The predictions of the CP5 and CP5-CR tunes are compared with data. The coloured band and error bars on the data points represent the total experimental uncertainty in the data

For the plots presented in Figs. 5, 6, 7, 8, 9, 10, 11, 12, 13, 14 in Sects. 4.1 and 4.2, inelastic events (i.e. ND, SD, DD, and CD) are simulated with pythia 8.226 and compared with data at different centre-of-mass energies. The rest of the plots are produced with pythia 8.235. An update to the description of the elastic scattering component in pythia 8.235 led to a slight decrease in the default ND cross section. The default ND cross section in pythia 8.226, which is 55.5$\text {\,mb}$ at $\sqrt{s}=13\,\text {Te\hspace{-.08em}V}$, is lowered to 55.1$\text {\,mb}$ in pythia 8.235. Hence, to reproduce the conditions of pythia 8.226 in pythia 8.235 or in a newer version, one should set the ND cross section manually.

### Underlying-event and minimum-bias observables

MB is a generic term used to describe events collected with a loose selection process that are dominated by relatively soft particles. Although these events generally correspond to inelastic scattering, including ND and SD+DD+CD processes, these contributions may vary depending on the trigger requirements used in the experiments. For example, a sample of non-single-diffractive-enhanced (NSD-enhanced) events is selected by suppressing the SD contribution at the trigger level.

The UE observables measured by the CMS experiment at $\sqrt{s}=13\,\text {Te\hspace{-.08em}V}$ [17], namely $N_{\textrm{ch}}$ density and the average $p_{\textrm{T}} ^{\text {sum}}$ in the transMIN and transMAX regions are well described by all tunes in the plateau region as shown in Fig. 5. The region up to ${\approx }5$ $\,\text {Ge\hspace{-.08em}V}$ of $p_{\textrm{T}} ^{\text {max}}$ is highly sensitive to diffractive contributions [30]. There is a lack of measurements in this region where the tunes, in general, do not perform well. Although the optimisation of these components is beyond the scope of this study, we have extended the fit range to ${\approx }$2–3$\,\text {Ge\hspace{-.08em}V}$ as long as the data are well described. The rising part of the spectrum excluding the region up to $\approx$5$\,\text {Ge\hspace{-.08em}V}$ of the $N_{\textrm{ch}}$ density distributions is similarly described by all tunes, whereas in the $p_{\textrm{T}} ^{\text {sum}}$ density distributions the predictions of CP5 differ slightly from the predictions of the CR tunes. These show that the CP5 tune has a harder $p_{\textrm{T}}$ spectrum at low $p_{\textrm{T}} ^{\text {max}}$ values. Through tuning the $N_{\textrm{ch}}$ and average $p_{\textrm{T}} ^{\text {sum}}$ density in the transMIN and transMAX regions, a satisfactory agreement is obtained for the same observables in the transDIFF region as well. Figure 6 shows the pseudorapidity distribution of charged hadrons in inelastic pp collisions measured by the CMS experiment at $\sqrt{s}=13\,\text {Te\hspace{-.08em}V}$ [22]. This observable is sensitive to the softer part of the MPI spectrum and well described by all tunes.

A crucial test for the performance of UE tunes, and of the CR simulation in particular, is the description of the average $p_{\textrm{T}}$ of the charged particles as a function of $N_{\textrm{ch}}$. Comparisons of the mean average $p_{\textrm{T}}$ to the measurements by the ATLAS Collaboration at $\sqrt{s}=13\,\text {Te\hspace{-.08em}V}$ in the transMAX and transMIN regions [18] are displayed in Fig. 7. The tune CP5 describes the central values of the data perfectly for $N_{\textrm{ch}} >7$, whereas the CR tunes show an almost constant discrepancy of 5–10% because of the harder $p_{\textrm{T}}$ spectrum predicted by the tune CP5 for low-$p_{\textrm{T}}$ particles. All CR tunes show a reasonable agreement with the data, confirming the accuracy of the parameters obtained for the new CR models. The improvement in the tuned CR models and their success in describing the data is seen by comparing Fig. 5 with Fig. 3, and Fig. 6 with Fig. 4. In these figures, CP5 tune predictions are also shown for easier comparison of CR tunes predictions with CP5.

In Fig. 8, charged-particle and $p_{\textrm{T}} ^{\text {sum}}$ densities measured by the CMS experiment at $\sqrt{s}=7\,\text {Te\hspace{-.08em}V}$ [19] in the transMIN and transMAX regions, as functions of $p_{\textrm{T}} ^{\text {max}}$, are compared with predictions from the tunes CP5 and CP5-CR. The data are reasonably well described for $p_{\textrm{T}} ^{\text {max}} >5\,\text {Ge\hspace{-.08em}V}$.

In Fig. 9, charged particle and $p_{\textrm{T}} ^{\text {sum}}$ densities in the transverse region, as functions of $p_{\textrm{T}} ^{\text {max}}$, and the average $p_{\textrm{T}}$ in the transverse region as functions of $p_{\textrm{T}} ^{\text {max}}$ and of the $N_{\textrm{ch}}$, measured by the ATLAS experiment at $\sqrt{s}=7\,\text {Te\hspace{-.08em}V}$ [20], are compared with the predictions from the tunes CP5 and CP5-CR. The central values of the average $p_{\textrm{T}}$ in bins of the leading charged particle $p_{\textrm{T}}$ and of the $N_{\textrm{ch}}$ are consistent with the data points within 10%. A similar level of agreement as observed at 13$\,\text {Te\hspace{-.08em}V}$ is achieved by the new tunes at 7$\,\text {Te\hspace{-.08em}V}$.

The performance of the new tunes is also checked at 7$\,\text {Te\hspace{-.08em}V}$ using inclusive measurements of charged-particle pseudorapidity distributions. In Fig. 10, the CMS measurements for $\text{ d }N_{\textrm{ch}}/\text{d }\eta$ at 7$\,\text {Te\hspace{-.08em}V}$  [31] with at least one charged particle in $|\eta |<2.4$ are compared with predictions from the tunes CP5 and CP5-CR. The CP5 and CP5-CR1 have similar predictions, while CP5-CR2 predicts about 4% less charged particles than the first two tunes in all $\eta$ bins of the measurement. Although all tunes provide a reasonable description of $\text{ d }N_{\textrm{ch}}/\text{d }\eta$ with deviations up to $\approx$10%, the data and MC simulation show different trends for $|\eta |>1.2$, where the trend for the data is not described well by the tunes. In the more central region, i.e. $|\eta |<1.2$, the shape of the predictions agrees well with the data but there is a difference in normalisation. For example, CP5 and CP5-CR1 predict 3–4% and CP5-CR2 predicts about 7% fewer charged particles in all bins for $|\eta |<1.2$ compared with the data.

In Fig. 11, charged-particle and $p_{\textrm{T}} ^{\text {sum}}$ densities measured as functions of $p_{\textrm{T}} ^{\text {max}}$ at $\sqrt{s}=1.96\,\text {Te\hspace{-.08em}V}$ by the CDF experiment [21] in the transMIN and transMAX regions are compared with predictions from the tunes CP5 and CP5-CR, respectively. All predictions reproduce the UE observables within $\approx$10% at $\sqrt{s}=1.96$, 7, and 13$\,\text {Te\hspace{-.08em}V}$.

We compare the new CMS tunes also with MB and UE data measured at forward pseudorapidities. The energy density, $\text{ d }E/\text{d }\eta$, measured in MB events and in NSD events by the CMS experiment at $\sqrt{s}=13\,\text {Te\hspace{-.08em}V}$, is shown in Fig. 12. The data are well described by CP5-CR2 within uncertainties and for all measured $|\eta |$ bins. The predictions of CP5 and CP5-CR1 overestimate the data in $4.2<|\eta |<4.9$.

The pseudorapidity of charged particles, $\text{ d }N_{\textrm{ch}}/\text{d }\eta$, in the ranges $|\eta |<2.2$ and $5.3<|\eta |<6.4$ measured by the CMS and TOTEM experiments at $\sqrt{s}=8\,\text {Te\hspace{-.08em}V}$ [32] is presented in Fig. 13. The events are required to have at least one charged particle in $5.3<\eta <6.5$ or $-6.5<\eta <-5.3$ with $p_{\textrm{T}} >0$. All tunes describe the data within the uncertainties. Additionally, Fig. 13 shows the pseudorapidity of charged particles, $\text{ d }N_{\textrm{ch}}/\text{d }\eta$, in $5.3<|\eta |<6.4$ in events with at least one charged particle with $p_{\textrm{T}} >40\,\text {Me\hspace{-.08em}V}$, measured by the TOTEM experiment at $\sqrt{s}=7\,\text {Te\hspace{-.08em}V}$ [33]. Both CP5 and CP5-CR1 describe the data within the uncertainties, whereas CP5-CR2 underestimates the data by 15%.

### Particle multiplicities

Figure 14 shows the strange particle production for $\varLambda$ baryons and $\text {K}_{\textrm{S}}^{0}$ mesons as a function of rapidity (*y*) measured by the CMS experiment [16] in NSD events at $\sqrt{s}=7\,\text {Te\hspace{-.08em}V}$. The rapidity is defined as $y=\frac{1}{2}\ln {\frac{E+p_L}{E-p_L}}$, where *E* is the particle energy and $p_L$ is the particle momentum along the anticlockwise-beam direction. It is shown in Ref. [14] that the new CR models might be beneficial for describing the ratios of strange particle multiplicities, for example $\varLambda /$ $\text {K}_{\textrm{S}}^{0}$ in pp collisions. We observe that all CP5 tunes, regardless of the CR model, describe particle production for $\text {K}_{\textrm{S}}^{0}$ mesons as a function of rapidity very well. However, they underestimate particle production for $\varLambda$ versus rapidity by about 30%. Therefore, the ratio $\varLambda /$ $\text {K}_{\textrm{S}}^{0}$ is not perfectly described but this could be improved by different hadronisation models [35, 36]. Including these observables, as well as the recent measurements of baryon production from the ALICE and LHCb experiments [37, 38], could be beneficial in future tune derivations. This is discussed in Appendix C.

**Fig. 15:**
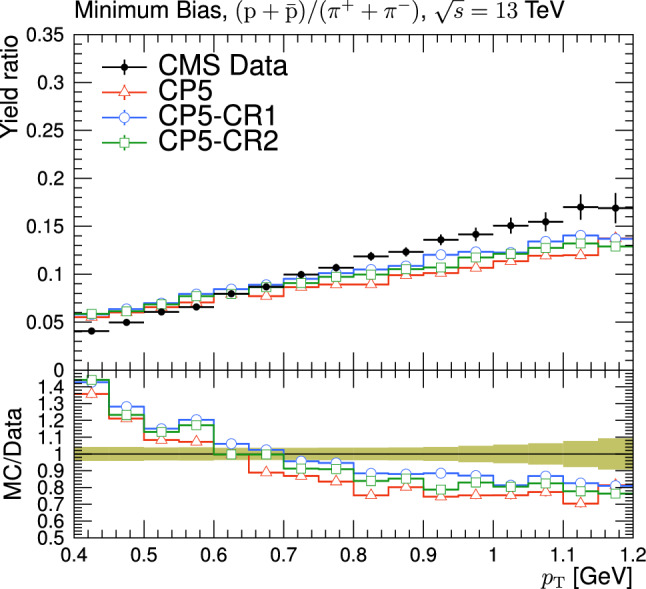
Ratios of particle yields, $\text {p}/\pi$, as a function of transverse momentum in minimum-bias events, measured by the CMS experiment at $\sqrt{s}=13\,\text {Te\hspace{-.08em}V}$ [39]. The predictions of the CP5 and CP5-CR tunes are compared with CMS data. The coloured band and error bars on the data points represent the total experimental uncertainty in the data

**Fig. 16:**
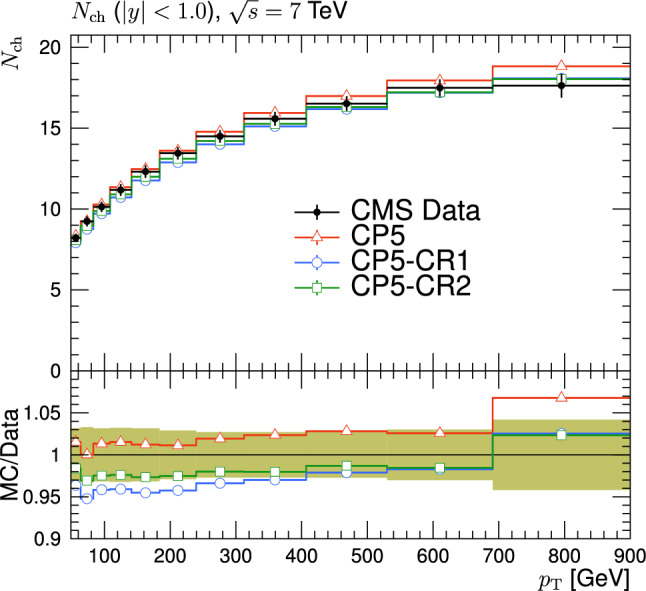
Average charged-hadron multiplicity, as a function of the jet $p_{\textrm{T}}$, for jets with rapidity $|y |<1$, measured by the CMS experiment at $\sqrt{s}=7\,\text {Te\hspace{-.08em}V}$ [27]. The predictions of the CP5 and CP5-CR tunes are compared with data. The coloured band and error bars on the data points represent the total experimental uncertainty in the data

**Fig. 17:**
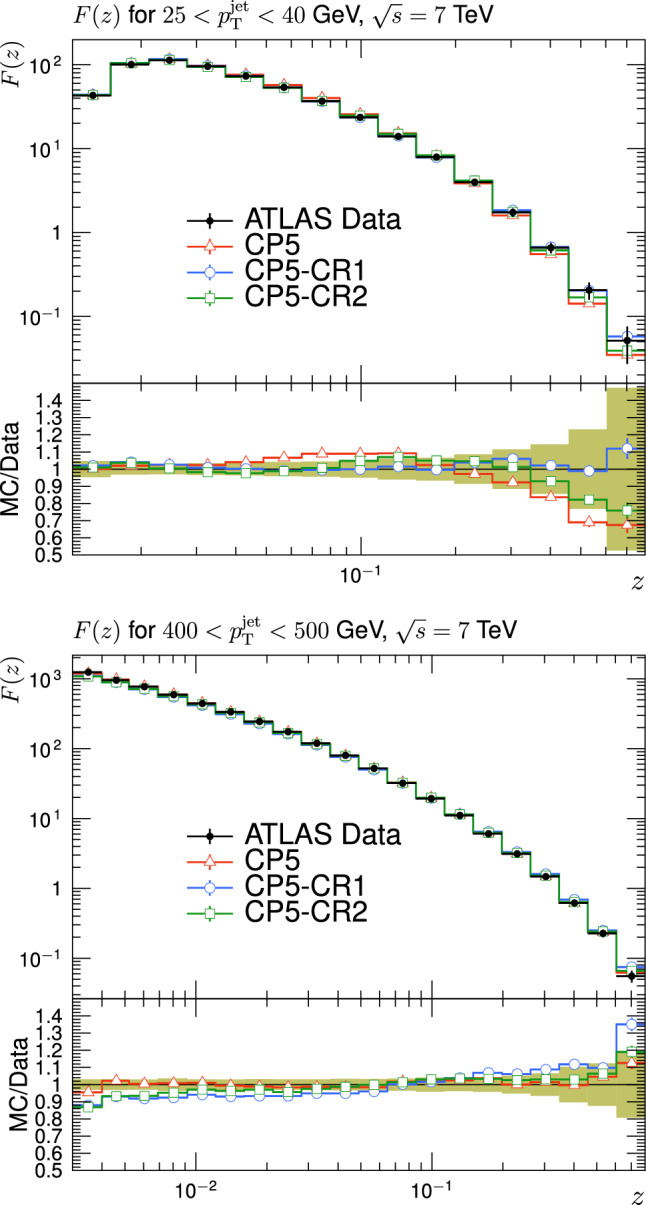
Distributions of *F*(*z*) for $25<p_{\textrm{T}} ^{\text {jet}} <40\,\text {Ge\hspace{-.08em}V}$ (upper) and $400<p_{\textrm{T}} ^{\text {jet}} <500\,\text {Ge\hspace{-.08em}V}$ (lower) for jets with pseudorapidity $|\eta _\text {jet} |<1.2$, measured by the ATLAS experiment at $\sqrt{s}=7\,\text {Te\hspace{-.08em}V}$ [28]. The predictions of the CP5 and CP5-CR tunes are compared with data. The coloured band and error bars on the data points represent the total experimental uncertainty in the data

The multiplicities of identified particles are also investigated in simulated MB events (ND+SD+DD+CD). Figure 15 shows the ratio of proton over pion production, as a function of particle $p_{\textrm{T}}$  [39]. All the tunes predict a similar trend, showing that the new CR models do not lead to a significant improvement in the description of the ratio of proton to pion production. However, it is known that this observable is strongly correlated with event particle multiplicity [39–41] and not only CR, since also hadronisation and MPI play a key role in describing the ratios of particle yields.

### Jet substructure observables

The number of charged particles contained in jets is an important observable that makes it possible to distinguish quark-initiated jets from gluon-initiated jets. The average number of charged hadrons with $p_{\textrm{T}} >500$ $\,\text {Me\hspace{-.08em}V}$ inside the jets measured by the CMS experiment as a function of the jet $p_{\textrm{T}}$ is shown in Fig. 16 [27]. The predictions of the CR tunes are comparable, and produce roughly 5% fewer charged particles than the CP5 tune. All predictions show a reasonable description of the data.

Figure 17 presents the distributions of $F(z)=(1/N_\text {jet})(\text{ d } N_{\textrm{ch}}/\text{d } z)$, where *z* is the longitudinal momentum fraction, and $N_{\textrm{ch}}$ is the charged-particle multiplicity in the jet, measured by the ATLAS experiment at $\sqrt{s}=7\,\text {Te\hspace{-.08em}V}$ [28]. The *F*(*z*) parameter is related to the fragmentation function and is presented for $p_{\textrm{T}} ^{\text {jet}} =25\text {--}40$ $\,\text {Ge\hspace{-.08em}V}$ and $p_{\textrm{T}} ^{\text {jet}} =400\text {--}500$ $\,\text {Ge\hspace{-.08em}V}$. The CR tunes describe low-$p_{\textrm{T}} ^{\text {jet}}$ data better than CP5, and their predictions reasonably agree with the high-$p_{\textrm{T}} ^{\text {jet}}$ data, except for the last bin. The high-$p_{\textrm{T}} ^{\text {jet}}$ data are well described by the CP5 tune within the uncertainties, and its central values agree better with the predictions of the CP5 tune than with those of the CP5-CR tunes.

### Drell–Yan events

**Fig. 18:**
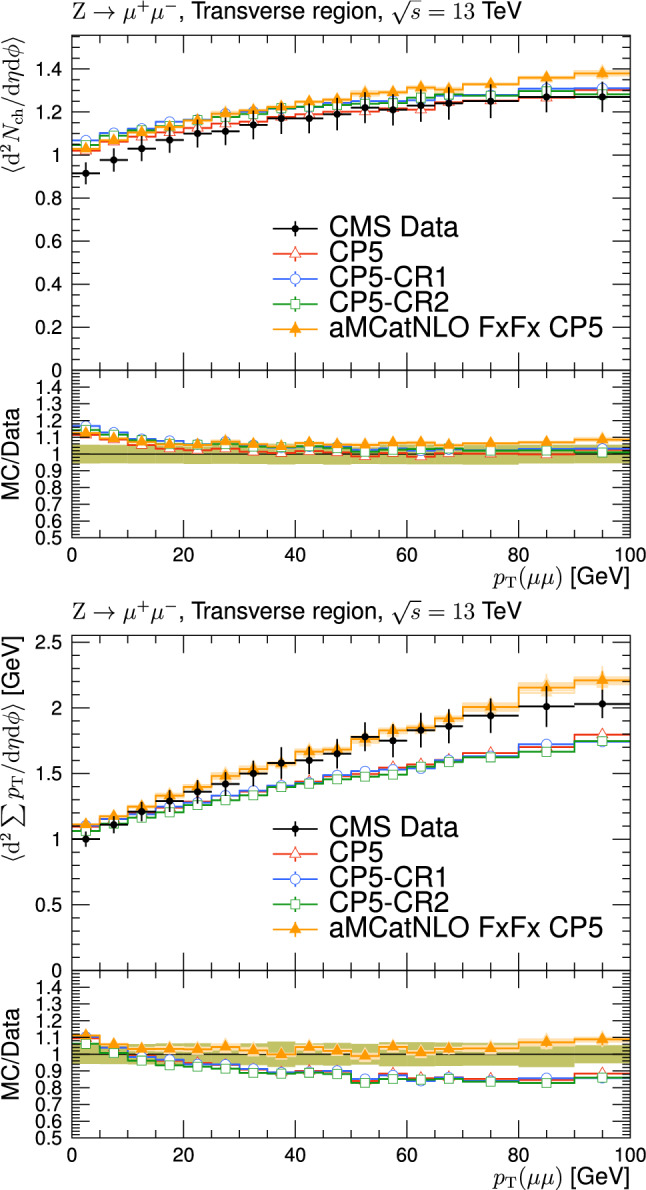
Number of charged particles and $p_{\textrm{T}}$ flow in the transverse region of DY events, measured by the CMS experiment at $\sqrt{s}=13\,\text {Te\hspace{-.08em}V}$ in bins of Z boson $p_{\textrm{T}}$  [44]. The plots show the predictions of pythia  8 with the CP5 and CP5-CR tunes, as well as MadGraph 5_amc@nlo with the CP5 tune compared with data. The coloured band and error bars on the data points represent the total experimental uncertainty in the data

Drell–Yan (DY) events [42, 43] with the Z boson decaying to ${{\upmu }}^{+} {{\upmu }}^{-}$ were generated with pythia  8 and compared with CMS data at $\sqrt{s}=13\,\text {Te\hspace{-.08em}V}$. Figure 18 shows the $N_{\textrm{ch}}$ and $p_{\textrm{T}}$ flow as a function of the Z boson $p_{\textrm{T}}$ (in the invariant ${{\upmu }}^{+} {{\upmu }}^{-}$ mass window of 81–101$\,\text {Ge\hspace{-.08em}V}$) in the region transverse to the boson momentum [44], which is expected to be dominated by the UE.

The CP5 tunes predict up to 15% too many charged particles at low Z boson $p_{\textrm{T}}$, where additional effects, such as the intrinsic transverse momentum of the interacting partons (i.e. primordial $k_{\textrm{T}}$) are expected to play a role. Higher-order corrections, as implemented in MadGraph 5_amc@nlo v2.4.2 [45] with FxFx merging [46], are necessary to describe the total $p_{\textrm{T}}$ flow. The impact of the different CR models is negligible in DY events.

### Top quark observables

#### Jet substructure in ${{\text {t}} {}{\bar{\text {t}}}}$ events

**Fig. 19:**
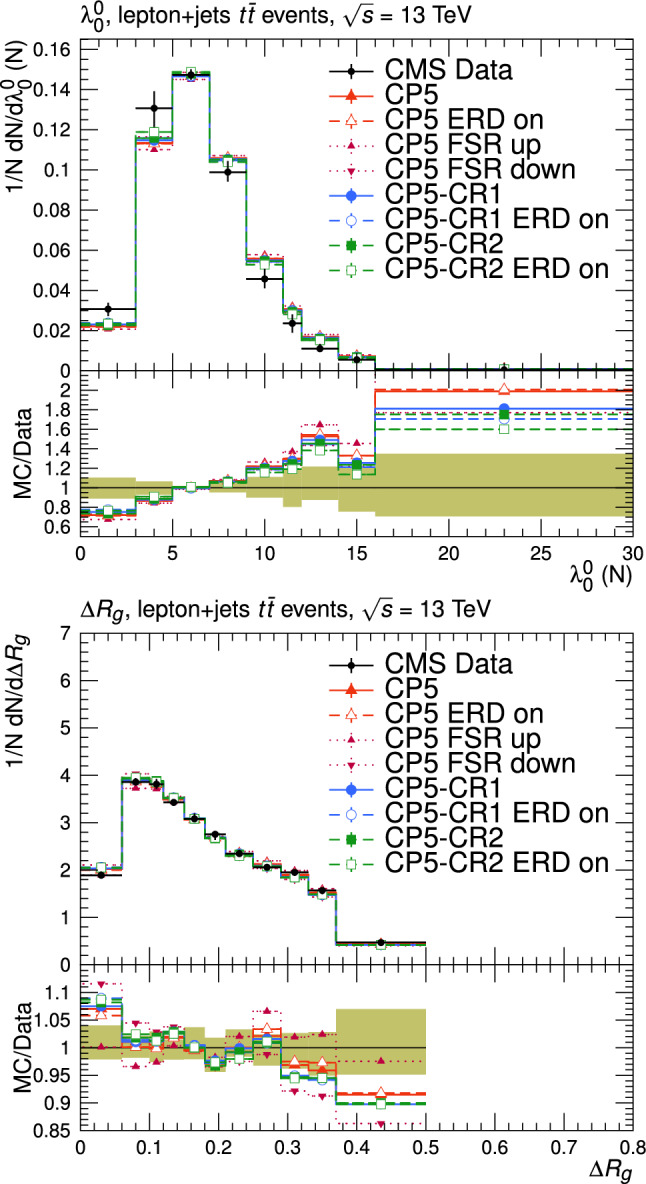
Distributions of the particle multiplicity in gluon jets (upper) and the angle $\varDelta R_g$ between two groomed subjets in inclusive jets (lower) measured by the CMS experiment in ${{\text {t}} {}{\bar{\text {t}}}}$ events at $\sqrt{s}=13\,\text {Te\hspace{-.08em}V}$ [53]. The coloured band and error bars on the data points represent the total experimental uncertainty in the data

A study of the UE in ${{\text {t}} {}{\bar{\text {t}}}}$ events [8] also estimated the effects of the CR on the top quark decay products by investigating the differences between predictions using pythia  8 with the ERD off and on options. In Ref.  [8], in addition to the QCD-inspired and gluon-move models, predictions of the rope hadronisation model [47, 48] are also compared with the data. In the rope hadronisation model, overlapping strings are treated to act coherently as a “rope”. The interactions between overlapping strings are described by an interaction potential inspired by the phenomenology of superconductors [35, 36, 47–52]. The ERD off and on options allow the CR to take place before or after the top quark decay, respectively. In particular, the ERD option allows the top quark decay products to be colour reconnected with the partons from MPI systems. Ref. [8] showed that these different models and options produce similar predictions for UE observables in ${{\text {t}} {}{\bar{\text {t}}}}$ events. However, some jet-shape distributions in ${{\text {t}} {}{\bar{\text {t}}}}$ events display a more significant effect [53], e.g. in the number of charged particles in jets. In the following, we investigate how the pythia  8 CR tunes describe the CMS ${{\text {t}} {}{\bar{\text {t}}}}$ jet substructure data [53]. In the CMS measurement, jets reconstructed using the anti-$k_{\textrm{T}}$ algorithm [54] with a distance parameter of $R=0.4$ as implemented in FASTJET 3.1 [55] are used. Jets with $p_{\textrm{T}} >30\,\text {Ge\hspace{-.08em}V}$ within $|\eta |<2$ are selected. Jet pairs ($\mathrm {j_1}$ and $\mathrm {j_2}$) are required to be far from each other in $\eta$-$\phi$ space, $\varDelta R(\mathrm {j_1},\mathrm {j_2}) = \sqrt{\smash [b]{(\eta _\mathrm {j_1}-\eta _\mathrm {j_2})^2 - (\phi _\mathrm {j_1}-\phi \mathrm {j_2})^2}}>0.8$. Jet substructure observables are calculated from jet constituents with $p_{\textrm{T}} >1\,\text {Ge\hspace{-.08em}V}$, e.g. in the plateau region of high track finding efficiency and low misidentification rate. Here we focus on two variables, (i) $\lambda _0^0(N)$, which is the number of charged particles with $p_{\textrm{T}} > 1\,\text {Ge\hspace{-.08em}V}$ in the jet, and (ii) the separation between two groomed subjets, $\varDelta R_g$, that are shown in Fig. 19 for gluon jets and inclusive jets, respectively. A “groomed jet” refers to a jet with soft and wide-angle radiation removed by a dedicated grooming algorithm [56, 57].

The compatibility of data and MC predictions is evaluated using a measure defined as $\chi ^2 = \varDelta ^T C^{-1} \varDelta$, where $\varDelta$ is the difference vector between measured and predicted values, and *C* is the total covariance matrix of the measurement. Since the measured distribution is normalised to unity, its covariance matrix is singular, i.e. not invertible. To render *C* invertible, the vector entry and matrix row/column corresponding to one measured bin need to be discarded; we choose to remove the last bin. The results are displayed in Table 4 for all jets inclusively as well as for each jet flavour separately. We observe that none of the tunes describe the $\lambda _0^0(N)$ data well for all jet flavours. As concluded in Ref. [53], flavour-dependent improvements in the nonperturbative physics modelling may be required for a better description of the data. The angle between the groomed subjets, on the other hand, is infrared and collinear safe and can be described very well by an increase in the $\alpha _\textrm{S} ^\textrm{FSR}(m_\text {Z})$, which corresponds to a decrease in the FSR renormalisation scale $\mu _R^\textrm{FSR}$. Table 4 shows the results obtained by varying $\mu _R^\textrm{FSR}$ by factors of 0.5 and 2.

**Table 4:** The $\chi ^2$ values and the numbers of degrees of freedom ($N_\textrm{dof}$) for the comparison of ${{\text {t}} {}{\bar{\text {t}}}}$ data with the predictions of the different pythia  8 tunes, for the distributions of the charged-particle multiplicity $\lambda _0^0$, the angle between the groomed subjets $\varDelta R_g$ at $\sqrt{s}$ = 13$\,\text {Te\hspace{-.08em}V}$  [53], and the pull angle measured in the ATLAS analysis of the colour flow at 8$\,\text {Te\hspace{-.08em}V}$  [58]. The FSR up and down entries denote variations of the renormalisation scale in the $\alpha _\textrm{S} ^\textrm{FSR}(m_\text {Z})$ by factors of 0.5 and 2, respectively

	Charged-particle multiplicity $\lambda _0^0$					Angle between groomed subjets $\varDelta R_g$					Pull angle $\phi (j_1,j_2)$	
	$\chi ^2$				$N_\textrm{dof}$	$\chi ^2$				$N_\textrm{dof}$	$\chi ^2$	$N_\textrm{dof}$
Tune	Incl.	Bottom	Light	Gluon		Incl.	Bottom	Light	Gluon			
CP5	18.4	26.6	30.7	11.8	8	28.2	18.3	10.6	8.1	10	4.7	3
CP5 ERD	19.6	28.7	32.2	12.2	8	26.9	15.0	10.7	8.7	10	2.4	3
CP5 FSR up	28.4	43.7	33.0	14.6	8	13.3	4.2	5.8	5.7	10	5.9	3
CP5 FSR down	15.0	19.7	44.0	11.6	8	59.6	39.2	33.1	22.6	10	4.1	3
CP5-CR1	14.3	28.4	29.5	4.1	8	34.6	13.4	24.4	23.6	10	3.9	3
CP5-CR1 ERD	11.7	24.4	27.8	3.8	8	32.7	13.4	21.1	27.0	10	1.4	3
CP5-CR2	14.1	23.8	38.3	8.1	8	34.3	22.3	21.3	11.7	10	5.2	3
CP5-CR2 ERD	11.0	16.9	38.6	7.1	8	35.3	24.8	16.1	13.1	10	9.3	3

#### Pull angle in ${{\text {t}} {}{\bar{\text {t}}}}$ events

**Fig. 20:**
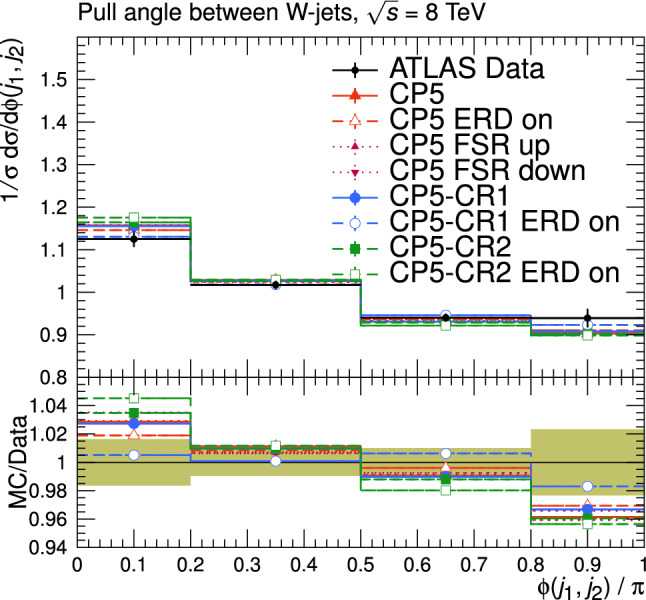
Normalised ${{\text {t}} {}{\bar{\text {t}}}}$ differential cross section for the pull angle between jets from the W boson in top quark decays, calculated from the charged constituents of the jets, measured by the ATLAS experiment using $\sqrt{s}=8\,\text {Te\hspace{-.08em}V}$ data [58] to investigate colour flow. The coloured band and error bars on the data points represent the total experimental uncertainty in the data

Figure 20 displays the normalised ${{\text {t}} {}{\bar{\text {t}}}}$ differential cross section for the jet pull angle [59] defined using the jets originating from the decay of a W boson in ${{\text {t}} {}{\bar{\text {t}}}}$ events, as measured by the ATLAS experiment [58]. The observable is shown for the case where only the charged constituents of the jet are used in the calculation. The data are compared with predictions from POWHEG v2 [60]+pythia  8 using the CP5 tunes or the corresponding CR tunes. The $\chi ^2$ values are calculated as described in Sect. 4.5.1 and are shown in the last column of Table 4. The pull angle is particularly sensitive to the setting of the ERD option. With ERD turned off, the decay products of the W boson in ${{\text {t}} {}{\bar{\text {t}}}}$ events are not included in CR, and the predictions using the tunes with the various CR models are similar to each other. With ERD enabled, CR now modifies the pull angle between the two jets, which is observed in Fig. 20. The predictions of each tune also show significant changes when ERD is enabled. For both the nominal and CP5-CR1 (QCD-inspired) tunes, the prediction with ERD improves the description of the data, and the difference between the predictions with or without ERD is larger for the CP5-CR1-based tune. We observe the opposite for the CP5-CR2-based (gluon-move) tunes, for which the choice without ERD is preferable. This picture might be different if the flip mechanism had been added in the tuning of the gluon-move model. The move step in the gluon-move model is more restrictive because it allows only gluons to move between the string end-points. The inclusion of the flip mechanism would also allow the string end-points to be mixed with each other and, therefore, could further reduce the total string length in an event. However, as indicated earlier, the effect of the flip mechanism on diffractive events is not well understood and, therefore, this mechanism is not used in this paper.

**Table 5:** The top quark mass ($m_{{\text {t}}}$) and W mass ($m_{\text {W}}$) extracted by a fit to the predictions of the different pythia  8 tunes, along with the differences from the nominal $m_{{\text {t}}}$ value ($\varDelta m_{{\text {t}}}$), $m_{\text {W}}$ value ($\varDelta m_{\text {W}}$), and $\varDelta m_{{\text {t}}} ^\text {hyb}$ which represents an estimation of the $m_{{\text {t}}}$ uncertainty considering the shift in $m_{\text {W}}$ included with a weight of 0.5. The uncertainties in the $m_{{\text {t}}}$ and $m_{\text {W}}$ values correspond to the uncertainty in the fitted $m_{{\text {t}}}$ and $m_{\text {W}}$

Tune	$m_{{\text {t}}}$ [$\text {Ge\hspace{-.08em}V}$ ]	$\varDelta m_{{\text {t}}}$ [$\text {Ge\hspace{-.08em}V}$ ]	$m_{\text {W}}$ [$\text {Ge\hspace{-.08em}V}$ ]	$\varDelta m_{\text {W}}$ [$\text {Ge\hspace{-.08em}V}$ ]	$\varDelta m_{{\text {t}}} ^\text {hyb}$ [$\text {Ge\hspace{-.08em}V}$ ]
CP5	$171.93\pm 0.02$	–	$79.76\pm 0.02$	–	–
CP5 ERD	$172.18\pm 0.03$	0.25	$80.15\pm 0.02$	0.40	0.05
CP5-CR1	$171.97\pm 0.02$	0.04	$79.74\pm 0.02$	$-0.02$	0.05
CP5-CR1 ERD	$172.01\pm 0.03$	0.08	$79.98\pm 0.02$	0.23	$-0.04$
CP5-CR2	$171.91\pm 0.02$	$-0.02$	$79.85\pm 0.02$	0.10	$-0.07$
CP5-CR2 ERD	$172.32\pm 0.03$	0.39	$79.90\pm 0.02$	0.14	0.32

Overall, the QCD-inspired model with ERD provides the best description of the jet pull angle. The differences between the predictions using the different tunes observed here indicate that the inclusion of observables, such as the jet pull angle and other jet substructure observables, could be beneficial in future tune derivations.

**Fig. 21:**
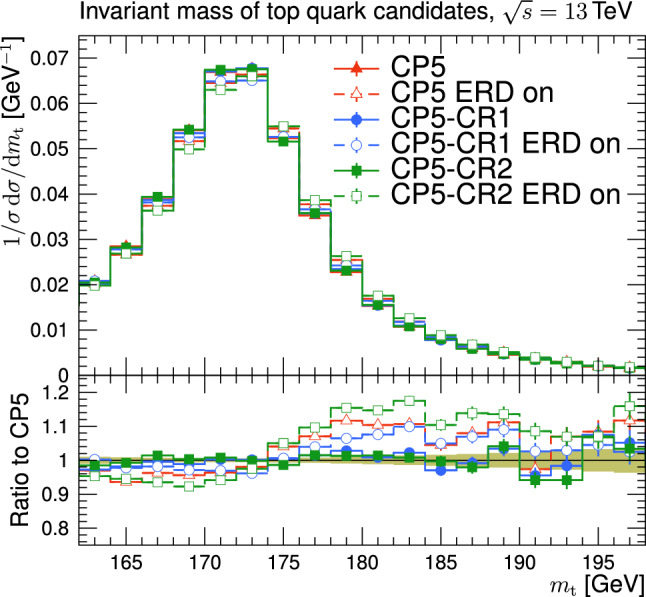
The invariant mass of hadronically decaying top quark candidates for different tune configurations. The coloured band and vertical bars represent the statistical uncertainty in the predictions

## Uncertainty in the top quark mass due to colour reconnection

The top quark mass has been measured with high precision using the 7, 8, and 13$\,\text {Te\hspace{-.08em}V}$  ${{\text {t}} {}{\bar{\text {t}}}}$ data at the LHC  [10, 61–73]. The most precise value of $m_{{\text {t}}} =172.44\pm 0.13\,\text {(stat)} \pm 0.47\,\text {(syst)}$ $\,\text {Ge\hspace{-.08em}V}$ was measured by the CMS Collaboration combining 7 and 8$\,\text {Te\hspace{-.08em}V}$ data [67]. To further improve the precision of $m_{{\text {t}}}$ measurements, a complete analysis of the systematic uncertainties in the measurement is crucial. One of the dominant systematic uncertainties is due to the modelling of CR in top quark decays [67]. The procedure for estimating this uncertainty used for the LHC Run 1 (years 2009–2013) analyses at $\sqrt{s}=7$ and 8$\,\text {Te\hspace{-.08em}V}$ was based on a comparison of two values of $m_{{\text {t}}}$, calculated by using predictions with the same UE tune with and without CR effects. In Ref. [67], this is done using the tune “Perugia 2011” with and without CR effects included. The “Perugia 2011” tunes family is the updated version of the “Perugia (Tevatron)” tunes family and also takes into account lessons learned from LHC MB and UE data at 0.9 and 7$\,\text {Te\hspace{-.08em}V}$  [74]. The new CMS tunes, presented in Sect. 2, which use different CR models, can be used to give a better evaluation of the CR uncertainty. In particular, the uncertainty is now calculated by comparing results for $m_{{\text {t}}}$ values obtained from different realistic CR models, such as CP5, CP5-CR1, and CP5-CR2.

Additionally, one can also estimate the effects of the CR on the top quark decay products by investigating the differences between predictions using pythia  8 with the option ERD off and on, which was done for the UE observables [8].

A determination of $m_{{\text {t}}}$ using a kinematic reconstruction of the decay products in semileptonic ${{\text {t}} {}{\bar{\text {t}}}}$ events at $\sqrt{s}=13\,\text {Te\hspace{-.08em}V}$ is reported in Ref. [10]. In these events, one of the W bosons from the top quark decays into a muon or electron and a neutrino, and the other into a quark–antiquark pair. In this analysis, $m_{{\text {t}}}$ and the jet energy scale factor were determined simultaneously through a joint-likelihood fit to the selected events. The results with the QCD-inspired and gluon-move models were also compared. The pythia  8 CUETP8M2T4 [75] UE tune was used, and the parameters of the CR models were tuned to UE and MB data at $\sqrt{s}=13\,\text {Te\hspace{-.08em}V}$ [10]. They found that the gluon-move model results in a 0.31$\,\text {Ge\hspace{-.08em}V}$ shift from the $m_{{\text {t}}}$ value obtained with the default simulation. This shift, which is larger than the shifts caused by the other CR models, is assumed to be the uncertainty due to the modelling of CR in the measured $m_{{\text {t}}}$. It is much larger than the shift, 0.01$\,\text {Ge\hspace{-.08em}V}$, due to the CR modelling in the Run 1 measurement [67]. This is the largest source of uncertainty in the measured $m_{{\text {t}}}$, where the total uncertainty is 0.62$\,\text {Ge\hspace{-.08em}V}$  [10]. Similar studies using single top quark final states are reported in Refs. [71, 76].

We compare the $m_{{\text {t}}}$ and W boson mass values obtained with different tune configurations based on our new tunes in Table 5. Top quark candidates are constructed by a Rivet routine in a sample of simulated semileptonic ${{\text {t}} {}{\bar{\text {t}}}}$ events. Events must contain exactly one lepton with $p_{\textrm{T}} >30\,\text {Ge\hspace{-.08em}V}$ and $|\eta |<2.1$. Leptons are “dressed” with the surrounding photons within a cone of $\varDelta R = 0.1$ and are required to yield an invariant mass window of 5$\,\text {Ge\hspace{-.08em}V}$ centred at 80.4$\,\text {Ge\hspace{-.08em}V}$, when combined with a neutrino in the event. The events must also contain at least four jets, reconstructed with the anti-$k_{\textrm{T}}$ algorithm, with $p_{\textrm{T}} >30\,\text {Ge\hspace{-.08em}V}$ within $|\eta |<2.4$. At least two of the jets are required to originate from the fragmentation of bottom quarks, and at least two other jets, referred to as light-quark jets, must not originate from bottom quarks. One jet originating from a bottom quark is combined with the lepton and neutrino to form a leptonically decaying top quark candidate, whereas the other jet originating from a bottom quark is combined with two other jets to form a hadronically decaying top quark candidate. The difference in the invariant mass window of the two top quark candidates is required to be less than 20$\,\text {Ge\hspace{-.08em}V}$, and the invariant mass of the two light-quark jets is within a window of 10$\,\text {Ge\hspace{-.08em}V}$ centred at 80.4$\,\text {Ge\hspace{-.08em}V}$. If more than one combination of jets satisfy these criteria when combined with the lepton and neutrino, then only one combination is chosen based on how similar the invariant masses of the two top quark candidates are to each other and on how close the invariant mass of the light-quark jets is to $80.4\,\text {Ge\hspace{-.08em}V}$. The invariant mass of the hadronically decaying top quark candidates constructed in this way for each of the different tune configurations is shown in Fig. 21. The top quark and W boson mass values are obtained from these hadronically decaying top quark candidates by fitting a Gaussian function within an 8$\,\text {Ge\hspace{-.08em}V}$ mass window around the corresponding mass peak. Table 5 also contains the differences from the nominal $m_{{\text {t}}}$ and $m_{\text {W}}$ values ($\varDelta m_{{\text {t}}}$, and $\varDelta m_{\text {W}}$) and the difference in $\varDelta m_{{\text {t}}} ^\text {hyb}$, a quantity that was introduced in Ref. [67] to incorporate both an in situ jet scale factor determined from the reconstructed $m_{\text {W}}$ as well as prior knowledge about the jet energy scale in a hybrid approach to extract $m_{{\text {t}}}$. Here, $\varDelta m_{{\text {t}}} ^\text {hyb}$ is approximated as $\varDelta m_{{\text {t}}}-0.5\varDelta m_{\text {W}}$. From Table 5, we observe that the largest deviation from the predictions of CP5 is CP5-CR2 ERD (0.32$\,\text {Ge\hspace{-.08em}V}$) similar to the largest shift found in Ref. [10] using CUETP8M2T4. However, CP5-CR2 ERD is not able to describe the available colour flow data, and can therefore be excluded from the list of modelling uncertainties.

## Summary and conclusion

New sets of parameters for two of the colour reconnection (CR) models implemented in the pythia  8 event generator, QCD-inspired and gluon-move, are obtained, based on the default CMS pythia  8 tune CP5. Measurements sensitive to underlying-event (UE) contributions performed at hadron-colliders at $\sqrt{s}=1.96$, 7, and 13$\,\text {Te\hspace{-.08em}V}$ are used to constrain the parameters for the CR and for the multiple-parton interactions simultaneously. Various measurements at 1.96, 7, 8, and 13$\,\text {Te\hspace{-.08em}V}$ are used to evaluate the performance of the new tunes. The central values predicted by the new CR tunes for the UE and minimum-bias events describe the data significantly better than the CR models with their default parameters before tuning. The predictions of the new tunes achieve a reasonable agreement in many UE observables, including the ones measured at forward pseudorapidities. However, the models after tuning do not generally perform better than the CP5 tune for the observables presented in this study. Although the new CR tunes presented in this work are not intended to improve the description of the measurements of strange particle multiplicities for $\varLambda$ baryons and $\text {K}_{\textrm{S}}^{0}$ mesons, we test the new tunes against them. We find that the new CR models, when tuned using only measurements that are sensitive to the UE, do not provide a better description of the distribution of strange particle production as a function of rapidity for $\varLambda$ baryons. However, we observe that all CP5 tunes, irrespective of the CR model, describe particle production for $\text {K}_{\textrm{S}}^{0}$ as a function of rapidity well. Including these observables in the fits, along with the latest measurements of baryon/meson production, could be beneficial for future tune derivations.

The predictions of the new tunes for jet shapes and colour flow measurements done with top quark pair events are also compared with data. All tunes give similar predictions, but none of the tunes describe the jet shape distributions well. Some differences are also observed with respect to the colour flow data, which is particularly sensitive to the early resonance decay option in the CR models. The differences between the predictions using the different tunes observed here indicate that the inclusion of observables, such as the jet pull angle and other jet substructure observables, could be beneficial in tuning studies. A study of the uncertainty in the top quark mass measurement due to CR effects is also presented. The new CR tunes will play a role in the evaluation of systematic uncertainties associated with the modelling of colour reconnection.

**Table 6:** List of input Rivet routines, centre-of-mass energy values, $\eta$ ranges, names of distributions, fit ranges, and relative importance of the distributions used in the fits to derive the tunes CP1-CR1 and CP1-CR2

Rivet routine	$\sqrt{s}$ [$\text {Te\hspace{-.08em}V}$ ]	$|\eta |$	Distribution	CP1-CR1 Fit range [$\text {Ge\hspace{-.08em}V}$ ]	*R*	CP1-CR2 Fit range [$\text {Ge\hspace{-.08em}V}$ ]	*R*
CMS_2015_I1384119	13	${<}2.0$	$N_{\textrm{ch}}$ versus $\eta$		1		1
CMS_2015_PAS_FSQ_15_007	13	${<}2.0$	TransMIN $p_{\textrm{T}} ^{\text {sum}}$	3–36	1	4–36	0.20
			TransMAX $p_{\textrm{T}} ^{\text {sum}}$	3–36	1	4–36	0.20
			TransMIN $N_{\textrm{ch}}$	3–36	1	4–36	0.20
			TransMAX $N_{\textrm{ch}}$	3–36	1	4–36	0.20
CMS_2012_PAS_FSQ_12_020	7	${<}0.8$	TransMAX $N_{\textrm{ch}}$	3–20	1	3–20	0.10
			TransMIN $N_{\textrm{ch}}$	3–20	1	3–20	0.10
			TransMAX $p_{\textrm{T}} ^{\text {sum}}$	3–20	1	3–20	0.10
			TransMIN $p_{\textrm{T}} ^{\text {sum}}$	3–20	1	3–20	0.10
CDF_2015_I1388868	2	${<}0.8$	TransMIN $N_{\textrm{ch}}$	2–15	1	2–15	0.10
			TransMAX $N_{\textrm{ch}}$	2–15	1	2–15	0.10
			TransMIN $p_{\textrm{T}} ^{\text {sum}}$	2–15	1	2–15	0.10
			TransMAX $p_{\textrm{T}} ^{\text {sum}}$	2–15	1	2–15	0.10

**Table 7:** List of input Rivet routines, centre-of-mass energy values, $\eta$ ranges, names of distributions, fit ranges, and relative importance of the distributions used in the fits to derive the tunes CP2-CR1 and CP2-CR2

Rivet routine	$\sqrt{s}$ [$\text {Te\hspace{-.08em}V}$ ]	$|\eta |$	Distribution	CP2-CR1 Fit range [$\text {Ge\hspace{-.08em}V}$ ]	*R*	CP2-CR2 Fit range [$\text {Ge\hspace{-.08em}V}$ ]	*R*
CMS_2015_I1384119	13	${<}2.0$	$N_{\textrm{ch}}$ versus $\eta$		0.03		0.05
CMS_2015_PAS_FSQ_15_007	13	${<}2.0$	TransMIN $p_{\textrm{T}} ^{\text {sum}}$	5–24	1	5–24	1
			TransMAX $p_{\textrm{T}} ^{\text {sum}}$	5–24	0.17	5–24	0.25
			TransMIN $N_{\textrm{ch}}$	5–24	1	5–24	1
			TransMAX $N_{\textrm{ch}}$	5–24	0.17	5–24	0.25
CMS_2012_PAS_FSQ_12_020	7	${<}0.8$	TransMAX $N_{\textrm{ch}}$	5–20	0.07	5–20	0.25
			TransMIN $N_{\textrm{ch}}$	5–20	1	5–20	1
			TransMAX $p_{\textrm{T}} ^{\text {sum}}$	5–20	0.07	5–20	0.25
			TransMIN $p_{\textrm{T}} ^{\text {sum}}$	5–20	1	5–20	1
CDF_2015_I1388868	2	${<}0.8$	TransMIN $N_{\textrm{ch}}$	2–15	0.03	2–15	0.05
			TransMAX $N_{\textrm{ch}}$	2–15	0.03	2–15	0.05
			TransMIN $p_{\textrm{T}} ^{\text {sum}}$	2–15	0.03	2–15	0.05
			TransMAX $p_{\textrm{T}} ^{\text {sum}}$	2–15	0.03	2–15	0.05

**Table 8:** The parameters obtained in the fits of the CP1-CR1 and CP1-CR2 tunes, compared with the ones of the tune CP1. The upper part of the table displays the fixed input parameters of the tune, while the lower part shows the fitted tune parameters. The number of degrees of freedom ($N_\textrm{dof}$) and the goodness of fit divided by the number of degrees of freedom are also shown

pythia 8 parameter	CP1 [13]	CP1-CR1	CP1-CR2
PDF set	NNPDF3.1 LO	NNPDF3.1 LO	NNPDF3.1 LO
$\alpha _\textrm{S} (m_\text {Z})$	0.130	0.130	0.130
SpaceShower:rapidityOrder	Off	Off	Off
MultipartonInteractions:ecmRef [$\text {Ge\hspace{-.08em}V}$ ]	7000	7000	7000
$\alpha _\textrm{S} ^\textrm{ISR}(m_\text {Z})$ value/order	0.1365/LO	0.1365/LO	0.1365/LO
$\alpha _\textrm{S} ^\textrm{FSR}(m_\text {Z})$ value/order	0.1365/LO	0.1365/LO	0.1365/LO
$\alpha _\textrm{S} ^\textrm{MPI}(m_\text {Z})$ value/order	0.130/LO	0.130/LO	0.130/LO
$\alpha _\textrm{S} ^\textrm{ME}(m_\text {Z})$ value/order	0.130/LO	0.130/LO	0.130/LO
StringZ:aLund	–	0.38	–
StringZ:bLund	–	0.64	–
StringFlav:probQQtoQ	–	0.078	–
StringFlav:probStoUD	–	0.2	–
SigmaTotal:zeroAXB	Off	Off	Off
BeamRemnants:remnantMode	–	1	–
MultipartonInteractions:bProfile	2	2	2
ColourReconnection:mode	–	1	2
MultipartonInteractions:pT0Ref [$\text {Ge\hspace{-.08em}V}$ ]	2.400	1.984	2.385
MultipartonInteractions:ecmPow	0.154	0.113	0.165
MultipartonInteractions:coreRadius	0.544	0.746	0.587
MultipartonInteractions:coreFraction	0.684	0.569	0.533
ColourReconnection:range	2.633	–	–
ColourReconnection:junctionCorrection	–	8.382	–
ColourReconnection:timeDilationPar	–	31.070	–
ColourReconnection:m0	–	1.845	–
ColourReconnection:m2lambda	–	–	2.769
ColourReconnection:fracGluon	–	–	0.979
$N_\textrm{dof}$	183	157	150
$\chi ^{*2} / N_\textrm{dof}$	0.89	0.73	0.20

**Table 9:** The parameters obtained in the fits of the CP2-CR1 and CP2-CR2 tunes, compared with the ones of the tune CP2. The upper part of the table displays the fixed input parameters of the tune, while the lower part shows the fitted tune parameters. The number of degrees of freedom ($N_\textrm{dof}$) and the goodness of fit divided by the number of degrees of freedom are also shown

pythia 8 parameter	CP2 [13]	CP2-CR1	CP2-CR2
PDF set	NNPDF3.1 LO	NNPDF3.1 LO	NNPDF3.1 LO
$\alpha _\textrm{S} (m_\text {Z})$	0.130	0.130	0.130
SpaceShower:rapidityOrder	Off	Off	Off
MultipartonInteractions:ecmRef [$\text {Ge\hspace{-.08em}V}$ ]	7000	7000	7000
$\alpha _\textrm{S} ^\textrm{ISR}(m_\text {Z})$ value/order	0.130/LO	0.130/LO	0.130/LO
$\alpha _\textrm{S} ^\textrm{FSR}(m_\text {Z})$ value/order	0.130/LO	0.130/LO	0.130/LO
$\alpha _\textrm{S} ^\textrm{MPI}(m_\text {Z})$ value/order	0.130/LO	0.130/LO	0.130/LO
$\alpha _\textrm{S} ^\textrm{ME}(m_\text {Z})$ value/order	0.130/LO	0.130/LO	0.130/LO
StringZ:aLund	–	0.38	–
StringZ:bLund	–	0.64	–
StringFlav:probQQtoQ	–	0.078	–
StringFlav:probStoUD	–	0.2	–
SigmaTotal:zeroAXB	Off	Off	Off
BeamRemnants:remnantMode	–	1	–
MultipartonInteractions:bProfile	2	2	2
ColourReconnection:mode	–	1	2
MultipartonInteractions:pT0Ref [$\text {Ge\hspace{-.08em}V}$ ]	2.306	2.154	2.287
MultipartonInteractions:ecmPow	0.139	0.119	0.146
MultipartonInteractions:coreRadius	0.376	0.538	0.514
MultipartonInteractions:coreFraction	0.327	0.599	0.525
ColourReconnection:range	2.323	–	–
ColourReconnection:junctionCorrection	–	0.761	–
ColourReconnection:timeDilationPar	–	13.080	–
ColourReconnection:m0	–	1.546	–
ColourReconnection:m2lambda	–	–	6.186
ColourReconnection:fracGluon	–	–	0.978
$N_\textrm{dof}$	183	117	118
$\chi ^{*2} / N_\textrm{dof}$	0.54	0.21	0.22

## Figures and Tables

**Fig. 22:**
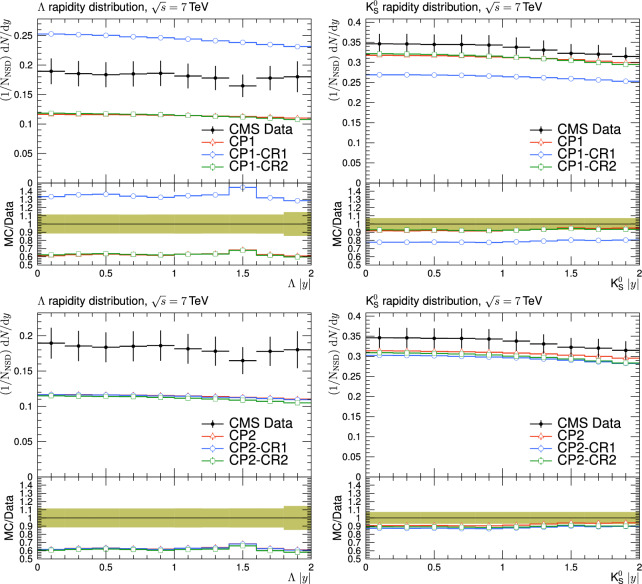
The strange particle production, $\varLambda$ baryons (left) and $\text {K}_{\textrm{S}}^{0}$ mesons (right), as a function of rapidity, measured by the CMS experiment at $\sqrt{s}=7\,\text {Te\hspace{-.08em}V}$ [16]. The predictions of the CP1 and CP1-CR tunes (upper) and CP2 and CP2-CR tunes (lower) are compared with data. The coloured band and error bars on the data points represent the total experimental uncertainty in the data

**Fig. 23:**
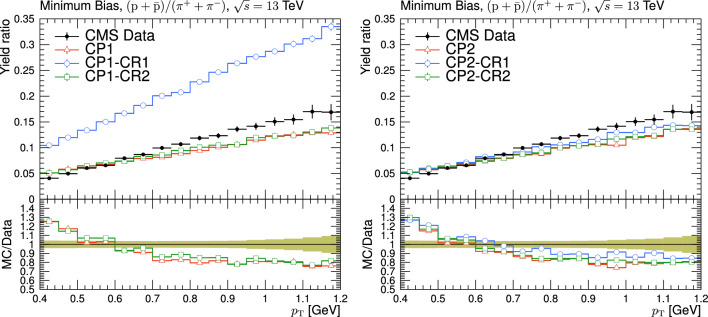
Ratios of particle yields, $p/\pi$, as a function of transverse momentum in MB events, measured by the CMS experiment at $\sqrt{s}=13\,\text {Te\hspace{-.08em}V}$ [39]. The predictions of the CP1 and CP1-CR tunes (left) and CP2 and CP2-CR tunes (right) are compared with data. The coloured band and error bars on the data points represent the total experimental uncertainty in the data

**Fig. 24:**
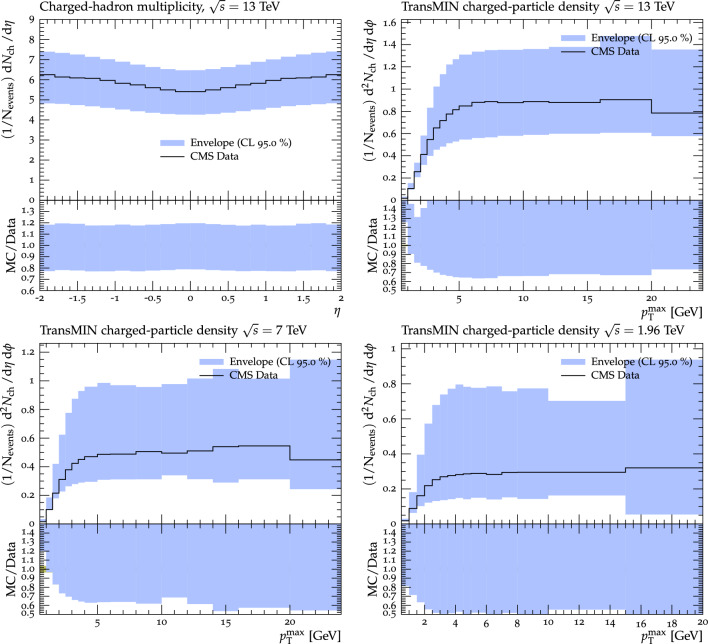
Sample histograms showing the range of variation available on the observable histograms are given for CP5-CR1

**Fig. 25:**
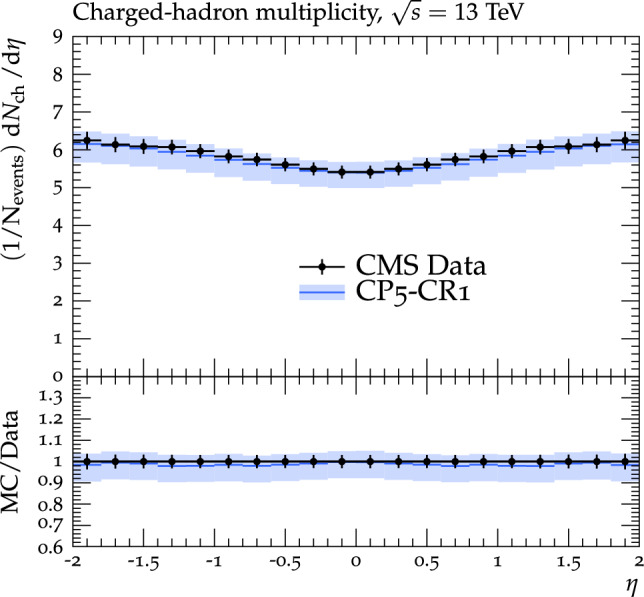
The pseudorapidity of charged hadrons, $\text{ d }N_{\textrm{ch}}/\text{d }\eta$, measured by the CMS experiment at $\sqrt{s}=13\,\text {Te\hspace{-.08em}V}$ [22]. The prediction of the CP5-CR1 tune is compared with data. The coloured band represents the tune uncertainties

**Fig. 26:**
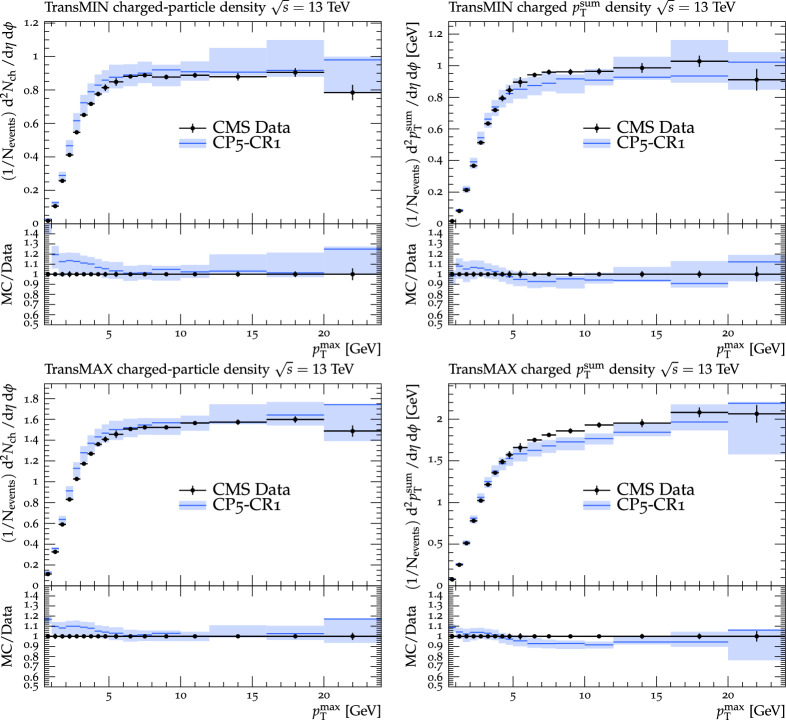
The charged-particle (left) and $p_{\textrm{T}} ^{\text {sum}}$ densities (right) in the transMIN (upper) and transMAX (lower) regions as functions of the $p_{\textrm{T}}$ of the leading charged particle, $p_{\textrm{T}} ^{\text {max}}$, measured by the CMS experiment at $\sqrt{s}=13\,\text {Te\hspace{-.08em}V}$ [17]. The predictions of the tunes CP5-CR1 are compared with data. The coloured band represents the tune uncertainties

**Fig. 27:**
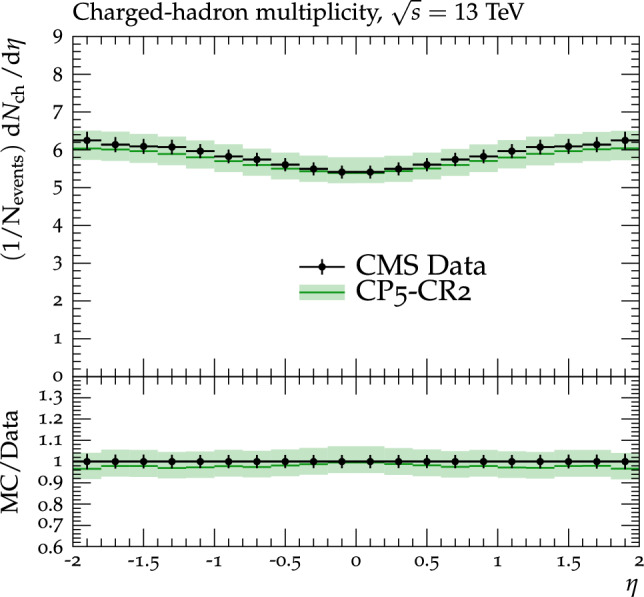
The pseudorapidity of charged hadrons, $\text{ d }N_{\textrm{ch}}/\text{d }\eta$, measured by the CMS experiment at $\sqrt{s}=13\,\text {Te\hspace{-.08em}V}$ [22]. The prediction of the CP5-CR2 tune is compared with data. The coloured band represents the tune uncertainties

**Fig. 28:**
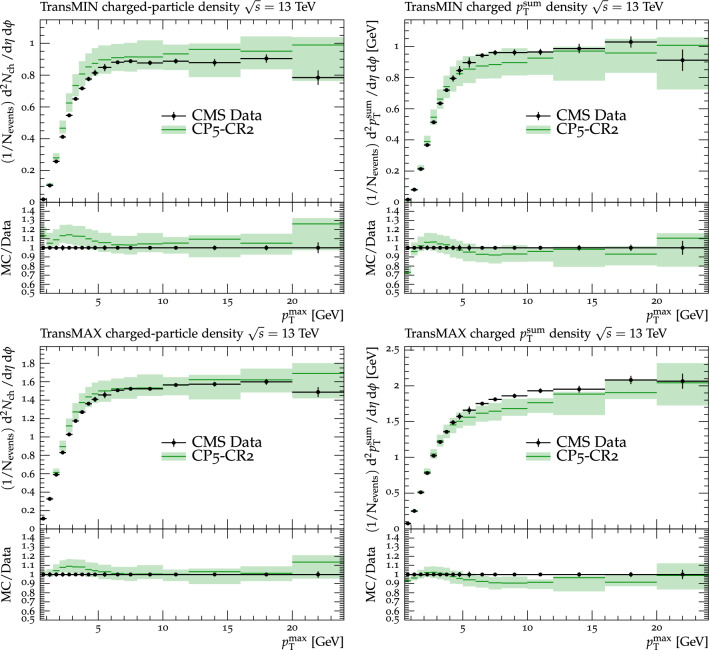
The charged-particle (left) and $p_{\textrm{T}} ^{\text {sum}}$ densities (right) in the transMIN (upper) and transMAX (lower) regions as functions of the $p_{\textrm{T}}$ of the leading charged particle, $p_{\textrm{T}} ^{\text {max}}$, measured by the CMS experiment at $\sqrt{s}=13\,\text {Te\hspace{-.08em}V}$ [17]. The predictions of the tunes CP5-CR2 are compared with data. The coloured band represents the tune uncertainties

**Fig. 29:**
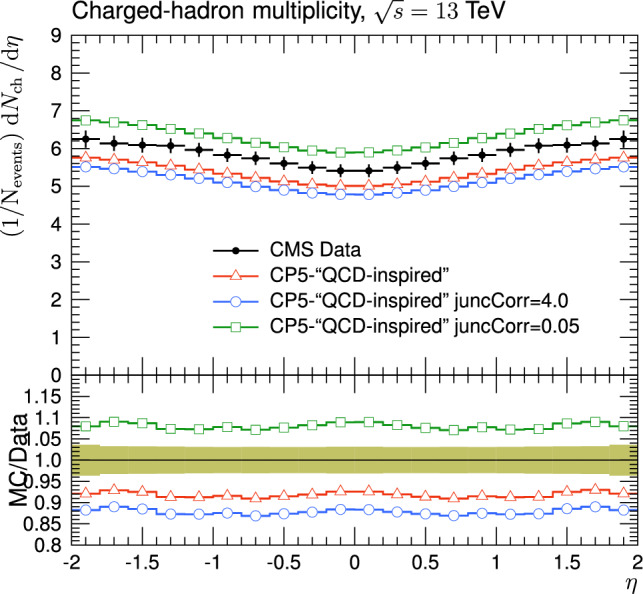
The pseudorapidity of charged hadrons, $\text{ d }N_{\textrm{ch}}/\text{d }\eta$, measured in $|\eta |<2$ by the CMS experiment at $\sqrt{s}=13\,\text {Te\hspace{-.08em}V}$ [23]. The red line shows the prediction of CP5-“QCD-inspired,” where the default value of the ColourReconnection:junctionCorrection parameter is 1.2. The predictions shown with the blue and green lines use 4.0 and 0.05 for the ColourReconnection:junctionCorrection parameter, respectively. These comparisons demonstrate the sensitivity of the ColourReconnection:junctionCorrection parameter to this observable

**Fig. 30:**
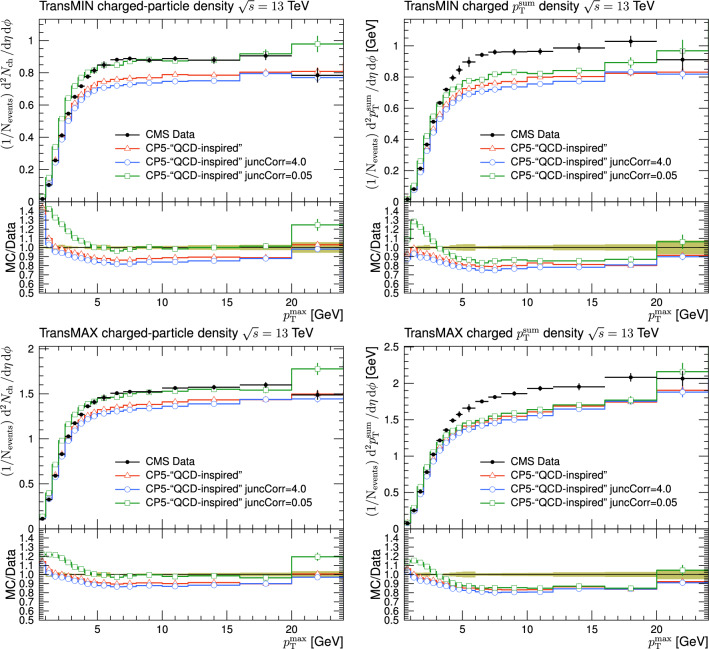
The charged-particle (left) and $p_{\textrm{T}} ^{\text {sum}}$ densities (right) in the transMIN (upper) and transMAX (lower) regions as functions of the $p_{\textrm{T}}$ of the leading charged particle, $p_{\textrm{T}} ^{\text {max}}$, measured by the CMS experiment at $\sqrt{s}=13\,\text {Te\hspace{-.08em}V}$ [17]. The red line shows the prediction of CP5-“QCD-inspired,” where the default value of the ColourReconnection:junctionCorrection parameter is 1.2. The predictions shown with the blue and green lines use 4.0 and 0.05 for the ColourReconnection:junctionCorrection parameter, respectively. These comparisons demonstrate the sensitivity of the ColourReconnection:junctionCorrection parameter to these observables

**Fig. 31:**
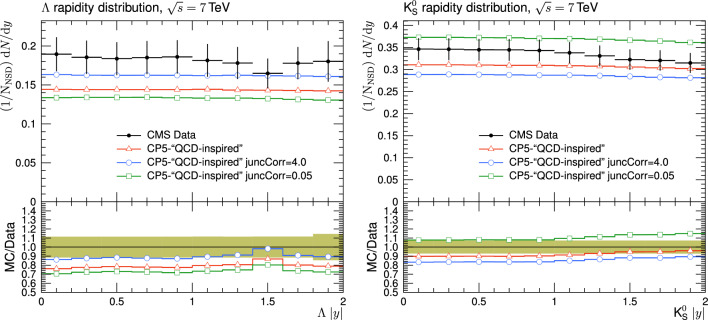
The strange particle production, $\varLambda$ baryons (left) and $\text {K}_{\textrm{S}}^{0}$ mesons (right), as a function of rapidity, measured by the CMS experiment at $\sqrt{s}=7\,\text {Te\hspace{-.08em}V}$ [16]. The red line shows the prediction of CP5-“QCD-inspired,” where the default value of the ColourReconnection:junctionCorrection parameter is 1.2. The predictions shown with the blue and green lines use 4.0 and 0.05 for the ColourReconnection:junctionCorrection parameter, respectively. These comparisons demonstrate the sensitivity of the ColourReconnection:junctionCorrection parameter to these observables

## Data Availability

This manuscript has no associated data or the data will not be deposited. [Authors’ comment: Release and preservation of data used by the CMS Collaboration as the basis for publications is guided by the CMS policy as stated in https://cms-docdb.cern.ch/cgi-bin/PublicDocDB/RetrieveFile?docid=6032 &filename=CMSDataPolicyV1.2.pdf &version=2 CMS data preservation, re-use and open access policy.]

## Colour reconnection tunes with a leading-order PDF set

The list of input Rivet routines used as inputs for the fits, as well as the centre-of-mass energy values, the $\eta$ ranges, the names of the distributions, the *x*-axis ranges, and the *R* values of the distributions are displayed in Table 6 for the tunes CP1-CR1 and CP1-CR2, and in Table 7 for the tunes CP2-CR1 and CP2-CR2. The baseline tunes CP1 and CP2 use the NNPDF31_lo_as_0130 [26] PDF set, with an $\alpha _\textrm{S} (m_\text {Z})$ value of 0.130 for ISR, FSR, and MPI, and the MPI-based CR model. The parameters of the tunes are documented in Ref. [13] and displayed in Tables 8 and 9. The parameters obtained from the CP1-CR1, CP1-CR2, CP2-CR1, and CP2-CR2 fits, as well as the value of the goodness of the fits are displayed in Tables 8 and 9. The predictions of these new CR tunes for particle multiplicities are shown in Figs. 22, and 23. The CR tunes based on CP1 and CP2 describe MB and UE data as well as the CR tunes based on CP5, except for the different trend observed with CP1-CR1 in the particle multiplicity distributions.

## Parameter ranges and uncertainties in the tunes

The parameter ranges are chosen such that the sampled MC space does not destroy the definition of a particular observable in the fits. In Fig. 24, some sample histograms showing the range of variation available on the observable histograms are given for CP5-CR1. The results are similar for other observables used in the fits as well as for CP5-CR2.

The CP5-CR1 and CP5-CR2 tunes were developed to evaluate the uncertainty in CP5 that results from different color reconnection models. The uncertainties in the parameters for these tunes were estimated using eigentunes provided by Professor. Eigentunes represent variations of the tuned parameters in the parameter space along the maximally independent directions. The magnitude of the variation corresponds to a change in the $\chi ^{*2}$ ($\varDelta \chi ^{*2}$) equal to the $\chi ^{*2}$ of the fit. The choice of $\varDelta \chi ^{*2}$, which is recommended by the Professor Collaboration, is based on empirical grounds since modifying it in equation Eq. (4) does not yield a statistically meaningful variation. Such a change, $\varDelta \chi ^{*2} = \chi ^{*2}$, is considered reasonable for reflecting the combined statistical and systematic uncertainty in the model parameters, and results in variations similar in magnitude to the uncertainties in the fitted data points. However, this approach may result in uncertainties that do not fully encompass the data in every bin. If the uncertainties in the fitted data points are uncorrelated, their magnitudes will depend on the bin widths. For the data used in the fit, the uncertainties are mostly correlated between bins. However, for UE observables with high $p_{\textrm{T}} ^{\text {max}}$ ($p_{\textrm{T}} ^{\text {max}} \gtrsim 10\,\text {Ge\hspace{-.08em}V}$) statistical uncertainties, which are uncorrelated between bins, dominate. This creates some dependence of the eigentunes on the bin widths of the data used in the fit, and leads to uncertainties in the tunes that are much larger and more asymmetric than those on the data points when all eigentunes are added in quadrature.

The number of eigentunes is equal to twice the number of free parameters used in the fit. For the QCD-inspired and gluon-move models, there are 14 and 12 eigentunes, respectively. However, using all 12 or 14 eigentunes to calculate the tune uncertainty for a given observable is computationally inefficient. Therefore, the “up” and “down” tune settings are calculated by comparing the positive and negative differences between each eigentune and the central prediction of the nominal tune for each bin of the observable. The upper and lower bounds of the uncertainty in each bin are defined by adding the positive differences in quadrature and taking the square root, and similarly for the negative differences. These “up” and “down” variations are then fit using the same procedure as in Sect. 3 to obtain new parameter sets that can be used to estimate the uncertainties in the nominal tune.

The predictions of the CP5-CR1 and CP5-CR2 tunes are compared with observables at 13$\,\text {Te\hspace{-.08em}V}$ in Figs. 25, 26, 27, 28. The shaded bands in these figures correspond to the envelope of the predictions of the eigentunes of each tune. The parameters of the “up” and “down” tunes for CP5-CR1 and CP5-CR2 are given in Tables 10 and 11, respectively.

**Table 10:** Parameters of the “up” and “down” variations of the CP5-CR1 tune

	CP5-CR1	
	Down	Up
MultipartonInteractions:pT0Ref [$\text {Ge\hspace{-.08em}V}$ ]	1.568	1.328
MultipartonInteractions:ecmPow	0.011	0.045
MultipartonInteractions:coreRadius	0.466	0.643
MultipartonInteractions:coreFraction	0.516	0.579
ColourReconnection:junctionCorrection	0.219	0.261
ColourReconnection:timeDilationPar	9.328	11.95
ColourReconnection:m0	1.786	1.529

**Table 11:** Parameters of the “up” and “down” variations of the CP5-CR2 tune

	CP5-CR2	
	Down	Up
MultipartonInteractions:pT0Ref [$\text {Ge\hspace{-.08em}V}$ ]	1.565	1.361
MultipartonInteractions:ecmPow	0.057	0.075
MultipartonInteractions:coreRadius	0.576	0.760
MultipartonInteractions:coreFraction	0.704	0.623
ColourReconnection:m2lambda	3.202	2.837
ColourReconnection:fracGluon	0.979	0.988

## The ColourReconnection:junctionCorrection parameter

The QCD-inspired model implemented in pythia  8 allows for the creation of string junctions when three color lines meet at a single point. The presence of these junctions can affect the number of particles produced in a collision and result in the production of additional gluons and quark–antiquark pairs. The junctionCorrection parameter in pythia  8 controls the strength of this effect and can impact various observables, including the charged particle pseudorapidity distribution.

The model predictions, with their default parameter settings in pythia 8.226 and CP5, are given in Fig. 29 for the $\text{ d }N_{\textrm{ch}}/\text{d }\eta$ distribution measured by the CMS experiment at 13$\,\text {Te\hspace{-.08em}V}$  [22], and in Fig. 30 for $N_{\textrm{ch}}$ and $p_{\textrm{T}} ^{\text {sum}}$ densities measured by CMS at 13$\,\text {Te\hspace{-.08em}V}$  [17] in the transMIN and transMAX regions. The predictions for CP5-“QCD-inspired” were obtained by replacing the MPI-based CR model in CP5 with the QCD-inspired model, where the default value of the junctionCorrection parameter is 1.2. The other predictions presented in the figures were obtained by setting the junctionCorrection parameter to 4.0 and to 0.05, respectively. These values were chosen arbitrarily to test how the prediction changes when a relatively high or low value is set for the junctionCorrection parameter. These comparisons demonstrate the sensitivity of the junctionCorrection parameter to these observables. According to Ref. [14], the junctionCorrection parameter is most sensitive to the baryon/meson ratio in pp collisions. The sensitivity of the junctionCorrection parameter to the production of $\varLambda$ baryons and $\text {K}_{\textrm{S}}^{0}$ mesons, measured by the CMS experiment at $\sqrt{s}=7\,\text {Te\hspace{-.08em}V}$ [16], is shown in Fig. 31.

We also derived a new version of CP5-CR1 by including the rapidity distributions of $\varLambda$ baryons and $\text {K}_{\textrm{S}}^{0}$ mesons, as well as some recent baryon and meson measurements from ALICE and LHCb experiments [37, 38]. The new tune, named CP5-CR2-v2, resulted in a significant improvement in the description of the $\varLambda$ rapidity distribution and reasonable agreement with the data for $\varLambda$/$\text {K}_{\textrm{S}}^{0}$, but it was not able to reproduce the $\text{ d }N_{\textrm{ch}}/\text{d }\eta$ at 13 TeV. The values of the parameters obtained in the fits of the CP5-CR1-v2 are presented in Table 12. The remaining parameters of pythia  8 are kept the same as in the CP5 tune.

**Table 12:** The values of the parameters obtained in the fits of the CP5-CR1-v2

pythia 8 parameterCP5-CR1-v2	
MultipartonInteractions:pT0Ref	1.260
MultipartonInteractions:ecmPow	0.042
MultipartonInteractions:coreRadius	0.750
MultipartonInteractions:coreFraction	0.567
ColourReconnection:m0	1.888
ColourReconnection:junctionCorrection	1.427
ColourReconnection:timeDilationPar	4.990

## References

[1] T. Sjöstrand et al., An introduction to PYTHIA 8.2. Comput. Phys. Commun. **191**, 159 (2015). 10.1016/j.cpc.2015.01.024. arXiv:1410.3012

[2] Corke R, Sjöstrand T (2011). Multiparton interactions with an x-dependent proton size. JHEP.

[3] Buckley A (2011). General-purpose event generators for LHC physics. Phys. Rep..

[4] Gustafson G (1986). Dual description of a confined color field. Phys. Lett. B.

[5] Particle Data Group Collaboration, Review of Particle Physics. PTEP **2020**(8), 083C01 (2020). 10.1093/ptep/ptaa104

[6] Sjöstrand T, van Zijl M (1987). A multiple interaction model for the event structure in hadron collisions. Phys. Rev. D.

[7] T. Sjöstrand, Colour reconnection and its effects on precise measurements at the LHC (2013). arXiv:1310.8073

[8] CMS Collaboration, Study of the underlying event in top quark pair production in pp collisions at 13 TeV. Eur. Phys. J. C **79**, 123 (2019). 10.1140/epjc/s10052-019-6620-z. arXiv:1807.02810 10.1140/epjc/s10052-019-6620-z PMC6380196 30863200

[9] Argyropoulos S, Sjöstrand T (2014). Effects of color reconnection on $$\rm {t\bar{t}}$$ final states at the LHC. JHEP.

[10] CMS Collaboration, Measurement of the top quark mass with lepton+jets final states using pp collisions at $$\sqrt{s}=13$$ TeV. Eur. Phys. J. C **78**, 891 (2018). 10.1140/epjc/s10052-018-6332-9. arXiv:1805.01428 10.1140/epjc/s10052-018-6332-9 PMC6394251 30881206

[11] Andersson B, Gustafson G, Ingelman G, Sjostrand T (1983). Parton fragmentation and string dynamics. Phys. Rep..

[12] CMS Collaboration, Event generator tunes obtained from underlying event and multiparton scattering measurements. Eur. Phys. J. C **76**, 155 (2016). 10.1140/epjc/s10052-016-3988-x. arXiv:1512.00815 10.1140/epjc/s10052-016-3988-x PMC4946872 27471433

[13] CMS Collaboration, Extraction and validation of a new set of CMS PYTHIA8 tunes from underlying-event measurements. Eur. Phys. J. C **80**, 4 (2020). 10.1140/epjc/s10052-019-7499-4. arXiv:1903.12179 10.1140/epjc/s10052-019-7499-4 PMC6944267 31976986

[14] Christiansen JR, Skands PZ (2015). String formation beyond leading colour. JHEP.

[15] Sjöstrand T, Skands PZ (2004). Multiple interactions and the structure of beam remnants. JHEP.

[16] CMS Collaboration, Strange particle production in pp collisions at $$\sqrt{s}=0.9$$ and 7 TeV. JHEP **05**, 064 (2011). 10.1007/JHEP05(2011)064. arXiv:1102.4282

[17] CMS Collaboration, Underlying event measurements with leading particles and jets in pp collisions at $$\sqrt{s}=13$$ TeV. CMS Physics Analysis Summary CMS-PAS-FSQ-15-007 (2015)

[18] ATLAS Collaboration, Measurement of charged-particle distributions sensitive to the underlying event in $$\sqrt{s}=13$$ TeV proton-proton collisions with the ATLAS detector at the LHC. JHEP **03**, 157 (2017). 10.1007/JHEP03(2017)157. arXiv:1701.05390

[19] CMS Collaboration, Measurement of the underlying event activity in pp collisions at the LHC at 7 TeV and comparison with 0.9 TeV. CMS Physics Analysis Summary CMS-PAS-FSQ-12-020 (2012)

[20] ATLAS Collaboration, Measurement of underlying event characteristics using charged particles in pp collisions at $$\sqrt{s}=900$$ GeV and 7 TeV with the ATLAS detector. Phys. Rev. D **83**, 112001 (2011). 10.1103/PhysRevD.83.112001. arXiv:1012.0791

[21] CDF Collaboration, Study of the energy dependence of the underlying event in proton-antiproton collisions. Phys. Rev. D **92**, 092009 (2015). 10.1103/PhysRevD.92.092009. arXiv:1508.05340

[22] CMS Collaboration, Pseudorapidity distribution of charged hadrons in proton-proton collisions at $$\sqrt{s}=13$$ TeV. Phys. Lett. B **751**, 143 (2015). 10.1016/j.physletb.2015.10.004. arXiv:1507.05915

[23] CMS Collaboration, Measurement of pseudorapidity distributions of charged particles in proton-proton collisions at $$\sqrt{s}=13$$ TeV by the CMS experiment. CMS Physics Analysis Summary CMS-PAS-FSQ-15-008 (2016)

[24] Buckley A (2013). Rivet user manual. Comput. Phys. Commun..

[25] Buckley A (2010). Systematic event generator tuning for the LHC. Eur. Phys. J. C.

[26] NNPDF Collaboration, Parton distributions from high-precision collider data. Eur. Phys. J. C **77**, 663 (2017). 10.1140/epjc/s10052-017-5199-5. arXiv:1706.00428 10.1140/epjc/s10052-017-5199-5 PMC6956957 31997920

[27] CMS Collaboration, Shape, transverse size, and charged hadron multiplicity of jets in pp collisions at 7 TeV. JHEP **06**, 160 (2012). 10.1007/JHEP06(2012)160. arXiv:1204.3170

[28] ATLAS Collaboration, Measurement of the jet fragmentation function and transverse profile in proton-proton collisions at a center-of-mass energy of 7 TeV with the ATLAS detector. Eur. Phys. J. C **71**, 1795 (2011). 10.1140/epjc/s10052-011-1795-y. arXiv:1109.5816

[29] ATLAS Collaboration, Measurement of the charged-particle multiplicity inside jets from $$\sqrt{s}=8$$ TeV pp collisions with the ATLAS detector. Eur. Phys. J. C **76**, 322 (2016). 10.1140/epjc/s10052-016-4126-5. arXiv:1602.00988 10.1140/epjc/s10052-016-4126-5 PMC5321259 28280438

[30] CMS and TOTEM Collaborations, Measurement of single-diffractive dijet production in proton-proton collisions at $$\sqrt{s}=8$$ TeV with the CMS and TOTEM experiments. Eur. Phys. J. C **80**, 1164 (2020). 10.1140/epjc/s10052-020-08562-y. arXiv:2002.12146 [Erratum: 10.1140/epjc/s10052-021-08863-w] 10.1140/epjc/s10052-020-08562-y PMC7746569 33362286

[31] CMS Collaboration, Pseudorapidity distributions of charged particles in pp collisions at $$\sqrt{s}=7$$ TeV with at least one central charged particle. CMS Physics Analysis Summary CMS-PAS-QCD-10-024 (2010)

[32] CMS, TOTEM Collaboration, Measurement of pseudorapidity distributions of charged particles in proton-proton collisions at $$\sqrt{s}$$ = 8 TeV by the CMS and TOTEM experiments. Eur. Phys. J. C **74**(10), 3053 (2014). 10.1140/epjc/s10052-014-3053-6. arXiv:1405.0722

[33] TOTEM Collaboration, Measurement of the forward charged particle pseudorapidity density in $$pp$$ collisions at $$\sqrt{s} = 7$$ TeV with the TOTEM experiment. Europhys. Lett. **98**(3), 31002 (2012). 10.1209/0295-5075/98/31002. arXiv:1205.4105

[34] CMS Collaboration, Measurement of the energy density as a function of pseudorapidity in proton-proton collisions at $$\sqrt{s}=13$$ TeV. Eur. Phys. J. C **79**, 391 (2019). 10.1140/epjc/s10052-019-6861-x. arXiv:1812.04095

[35] C. Bierlich, G. Gustafson, L. Lönnblad, A shoving model for collectivity in hadronic collisions (2016). arXiv:1612.05132

[36] C. Bierlich, Rope hadronization and strange particle production. Eur. Phys. J. Web Conf. **171**, 14003 (2018). 10.1051/epjconf/201817114003. arXiv:1710.04464

[37] ALICE Collaboration, Measurement of prompt D$$^{0}$$, $$\Lambda _{c}^{+}$$, and $$\Sigma _{c}^{0,++}$$(2455) production in proton-proton collisions at $$\sqrt{s}=13$$ TeV. Phys. Rev. Lett. ** 128**, 012001 (2022). 10.1103/PhysRevLett.128.012001. arXiv:2106.08278 10.1103/PhysRevLett.128.012001 35061479

[38] LHCb Collaboration, Measurement of $$b$$ hadron fractions in 13 TeV pp collisions. Phys. Rev. D **100**, 031102 (2019). 10.1103/PhysRevD.100.031102. arXiv:1902.06794

[39] CMS Collaboration, Measurement of charged pion, kaon, and proton production in proton-proton collisions at $$\sqrt{s}=13$$ TeV. Phys. Rev. D **96**, 112003 (2017). 10.1103/PhysRevD.96.112003. arXiv:1706.10194

[40] CMS Collaboration, Study of the inclusive production of charged pions, kaons, and protons in pp collisions at $$\sqrt{s}=0.9$$, 2.76, and 7 TeV. Eur. Phys. J. C **72**, 2164 (2012). 10.1140/epjc/s10052-012-2164-1. arXiv:1207.4724

[41] CMS Collaboration, Study of the production of charged pions, kaons, and protons in pPb collisions at $$\sqrt{s_{NN}}=5.02$$ TeV. Eur. Phys. J. C **74**, 2847 (2014). 10.1140/epjc/s10052-014-2847-x. arXiv:1307.3442 10.1140/epjc/s10052-014-2847-x PMC4370925 25814892

[42] S.D. Drell, T.-M. Yan, Massive lepton pair production in hadron-hadron collisions at high-energies. Phys. Rev. Lett. **25**, 316 (1970). 10.1103/PhysRevLett.25.316 [Erratum: Phys. Rev. Lett. 25, 902 (1970)]

[43] Christenson JH (1970). Observation of massive muon pairs in hadron collisions. Phys. Rev. Lett..

[44] CMS Collaboration, Measurement of the underlying event activity in inclusive Z boson production in proton-proton collisions at $$\sqrt{s}=13$$ TeV. JHEP **07**, 032 (2018). 10.1007/JHEP07(2018)032. arXiv:1711.04299

[45] Alwall J (2014). The automated computation of tree-level and next-to-leading order differential cross sections, and their matching to parton shower simulations. JHEP.

[46] Frederix R, Frixione S (2012). Merging meets matching in MC@NLO. JHEP.

[47] Bierlich C, Gustafson G, Lönnblad L, Tarasov A (2015). Effects of overlapping strings in pp collisions. JHEP.

[48] C. Bierlich, J.R. Christiansen, Effects of color reconnection on hadron flavor observables. Phys. Rev. D **92**, 094010 (2015). 10.1103/PhysRevD.92.094010. arXiv:1507.02091

[49] C. Bierlich, G. Gustafson, L. Lönnblad, Collectivity without plasma in hadronic collisions. Phys. Lett. B **779**, 58 (2018). 10.1016/j.physletb.2018.01.069. arXiv:1710.09725

[50] Abramovsky VA, Gedalin EV, Gurvich EG, Kancheli OV (1988). Long range azimuthal correlations in multiple production processes at high-energies. JETP Lett..

[51] I. Altsybeev, Mean transverse momenta correlations in hadron-hadron collisions in MC toy model with repulsing strings. AIP Conf. Proc. **1701**, 100002 (2016). 10.1063/1.4938711. arXiv:1502.03608

[52] Cea P, Cosmai L, Cuteri F, Papa A (2014). Flux tubes in the SU(3) vacuum: London penetration depth and coherence length. Phys. Rev. D.

[53] CMS Collaboration, Measurement of jet substructure observables in $${{\rm t}}\overline{{\rm t}}$$ events from proton-proton collisions at $$\sqrt{s}=$$ 13 TeV. Phys. Rev. D **98**, 092014 (2018). 10.1103/PhysRevD.98.092014. arXiv:1808.07340

[54] Cacciari M, Salam GP, Soyez G (2008). The anti-$${k_{\rm T }}$$ jet clustering algorithm. JHEP.

[55] Cacciari M, Salam GP, Soyez G (2012). FastJet user manual. Eur. Phys. J. C.

[56] Dasgupta M, Fregoso A, Marzani S, Salam GP (2013). Towards an understanding of jet substructure. JHEP.

[57] Larkoski AJ, Marzani S, Soyez G, Thaler J (2014). Soft drop. JHEP.

[58] ATLAS Collaboration, Measurement of colour flow with the jet pull angle in $${\rm t\bar{t}}$$ events using the ATLAS detector at $$\sqrt{s}=8$$ TeV. Phys. Lett. B ** 750**, 475 (2015). 10.1016/j.physletb.2015.09.051. arXiv:1506.05629

[59] Gallicchio J, Schwartz MD (2010). Seeing in color: jet superstructure. Phys. Rev. Lett..

[60] Frixione S, Nason P, Ridolfi G (2007). A positive-weight next-to-leading-order Monte Carlo for heavy flavour hadroproduction. JHEP.

[61] ATLAS, CDF, CMS, and D0 Collaborations, First combination of Tevatron and LHC measurements of the top-quark mass, Technical Reports ATLAS-CONF-2014-008, CDF-NOTE-11071, CMS-PAS-TOP-13-014, D0-NOTE-6416, FERMILAB-TM-2582-E (2014). arXiv:1403.4427

[62] ATLAS Collaboration, Measurement of the top-quark mass in the fully hadronic decay channel from ATLAS data at $$\sqrt{s}=7$$ TeV. Eur. Phys. J. C **75**, 158 (2015). 10.1140/epjc/s10052-015-3373-1. arXiv:1409.0832 10.1140/epjc/s10052-015-3373-1 PMC4446089 26041974

[63] ATLAS Collaboration, Measurement of the top quark mass in the $${\rm t\bar{t}}\rightarrow \text{lepton+jets} $$ and $${\rm t\bar{t}}\rightarrow \text{ dilepton } $$ channels using $$\sqrt{s}=7$$ tev ATLAS data. Eur. Phys. J. C **75**, 330 (2015). 10.1140/epjc/s10052-015-3544-0. arXiv:1503.05427 10.1140/epjc/s10052-015-3544-0 PMC4509706 26213487

[64] ATLAS Collaboration, Measurement of the top quark mass in the $${\rm t\bar{t}}\rightarrow $$ dilepton channel from $$\sqrt{s}=8$$ TeV ATLAS data. Phys. Lett. B **761**, 350 (2016). 10.1016/j.physletb.2016.08.042. arXiv:1606.02179

[65] ATLAS Collaboration, Top-quark mass measurement in the all-hadronic $${\rm t\overline{t} }$$ decay channel at $$\sqrt{s}=8$$ TeV with the ATLAS detector. JHEP **09**, 118 (2017). 10.1007/JHEP09(2017)118. arXiv:1702.07546

[66] ATLAS Collaboration, Measurement of the top quark mass in the $${\rm t\bar{t}}\rightarrow $$ lepton+jets channel from $$\sqrt{s}=8$$ TeV ATLAS data and combination with previous results. Eur. Phys. J. C **79**, 290 (2019). 10.1140/epjc/s10052-019-6757-9. arXiv:1810.01772

[67] CMS Collaboration, Measurement of the top quark mass using proton-proton data at $$\sqrt{s}=7$$ and 8 TeV. Phys. Rev. D **93**, 072004 (2016). 10.1103/PhysRevD.93.072004. arXiv:1509.04044

[68] CMS Collaboration, Measurement of the top-quark mass in $${\rm t}{\bar{\text{ t }}}$$ events with lepton+jets final states in pp collisions at $$\sqrt{s}=7$$ TeV. JHEP **12**, 105 (2012). 10.1007/JHEP12(2012)105. arXiv:1209.2319

[69] CMS Collaboration, Measurement of the top-quark mass in $${\rm t}{\bar{\text{ t }}}$$ events with dilepton final states in pp collisions at $$\sqrt{s}=7$$ TeV. Eur. Phys. J. C **72**, 2202 (2012). 10.1140/epjc/s10052-012-2202-z. arXiv:1209.2393

[70] CMS Collaboration, Measurement of the top-quark mass in all-jets $${\rm t}{\bar{\text{ t }}}$$ events in pp collisions at $$\sqrt{s}=7$$ TeV. Eur. Phys. J. C **74**, 2758 (2014). 10.1140/epjc/s10052-014-2758-x. arXiv:1307.4617 10.1140/epjc/s10052-014-2758-x PMC4370827 25814885

[71] CMS Collaboration, Measurement of the top quark mass using single top quark events in proton-proton collisions at $$\sqrt{s}=8$$ TeV. Eur. Phys. J. C **77**, 354 (2017). 10.1140/epjc/s10052-017-4912-8. arXiv:1703.02530 10.1140/epjc/s10052-017-4912-8 PMC5586548 28943789

[72] CMS Collaboration, Measurement of the $${{\rm t}}\overline{\rm t}$$ production cross section, the top quark mass, and the strong coupling constant using dilepton events in pp collisions at $$\sqrt{s}=13$$ TeV. Eur. Phys. J. C **79**, 368 (2019). 10.1140/epjc/s10052-019-6863-8. arXiv:1812.10505 10.1140/epjc/s10052-019-6863-8 PMC6507419 31148943

[73] CMS Collaboration, Measurement of the top quark mass in the all-jets final state at $$\sqrt{s}=13$$ TeV and combination with the lepton+jets channel. Eur. Phys. J. C **79**(313) (2019). 10.1140/epjc/s10052-019-6788-2. arXiv:1812.10534 10.1140/epjc/s10052-019-6788-2 PMC6454813 31031568

[74] Skands PZ (2010). Tuning Monte Carlo generators: the Perugia tunes. Phys. Rev. D.

[75] CMS Collaboration, Investigations of the impact of the parton shower tuning in PYTHIA8 in the modelling of $${{\rm t}}\overline{{\rm t}}$$ at $$\sqrt{s}=8$$ and 13 TeV. CMS Physics Analysis Summary CMS-PAS-TOP-16-021 (2016)

[76] CMS Collaboration, Measurement of the top quark mass using events with a single reconstructed top quark in pp collisions at $$\sqrt{s}=13$$ TeV. JHEP **12**(161) (2021). 10.1007/JHEP12(2021)161. arXiv:2108.10407

